# SEXUAL SPECIES ARE SEPARATED BY LARGER GENETIC GAPS THAN ASEXUAL SPECIES IN ROTIFERS

**DOI:** 10.1111/evo.12483

**Published:** 2014-07-25

**Authors:** Cuong Q Tang, Ulrike Obertegger, Diego Fontaneto, Timothy G Barraclough

**Affiliations:** 1 Department of Life Sciences, Imperial College London, AscotBerkshire, SL5 7PY, United Kingdom; 3 Department of Sustainable Agro-ecosystems and Bioresources, Research and Innovation Centre, Fondazione Edmund Mach (FEM)Via E. Mach 1, 38010 San Michele all’Adige, Italy; 4 Institute of Ecosystem Study, National Research Council28922 Verbania Pallanza, Italy

**Keywords:** Biodiversity, diversification rate, ecological speciation, GMYC, species delimitation

## Abstract

Why organisms diversify into discrete species instead of showing a continuum of genotypic and phenotypic forms is an important yet rarely studied question in speciation biology. Does species discreteness come from adaptation to fill discrete niches or from interspecific gaps generated by reproductive isolation? We investigate the importance of reproductive isolation by comparing genetic discreteness, in terms of intra- and interspecific variation, between facultatively sexual monogonont rotifers and obligately asexual bdelloid rotifers. We calculated the age (phylogenetic distance) and average pairwise genetic distance (raw distance) within and among evolutionarily significant units of diversity in six bdelloid clades and seven monogonont clades sampled for 4211 individuals in total. We find that monogonont species are more discrete than bdelloid species with respect to divergence between species but exhibit similar levels of intraspecific variation (species cohesiveness). This pattern arises because bdelloids have diversified into discrete genetic clusters at a faster net rate than monogononts. Although sampling biases or differences in ecology that are independent of sexuality might also affect these patterns, the results are consistent with the hypothesis that bdelloids diversified at a faster rate into less discrete species because their diversification does not depend on the evolution of reproductive isolation.

Genetic, morphological, and behavioral evidence have accumulated to show that species are real entities maintained by ecological differences and reproductive isolation (Schluter 2001; Coyne and Orr 2004; Puritz et al. 2012). Species are a fundamental unit of biology, and the existence of discontinuous groups of organisms, as opposed to a continuum of genotypes and phenotypes, is regarded as a ubiquitous phenomenon of life (Rieseberg et al. 2006). Why this discreteness exists at all is a neglected question and “perhaps the most important question about speciation” (Coyne and Orr 2004, p. 49).

Maynard Smith and Száthmary (1995) proposed two nonmutually exclusive hypotheses regarding the existence of discrete species: (1) Species exist because they fill distinct ecological niches. Differences in resource use impose divergent selection pressures on organisms and therefore distinct genotypic and phenotypic solutions evolve (reviewed in Schluter 2001). Organisms adapting to one niche are less suited to a second, and intermediate genotypes are poorly adapted to either niche. (2) Species exist as a consequence of sexual reproduction. Given enough time, reproductive isolation is an inevitable by-product of genetic divergence through selection or drift (Fisher 1930; Dobzhansky 1937; Rice and Hostert 1993). Reproductive isolation breaks up the continuum of genotypic and phenotypic diversity, and prevents the formation of hybrid forms (i.e., hybrid inferiority—Burke and Arnold 2001), whereas sexual reproduction maintains genetic coherence within species.

In sexual organisms, the predictions of these two hypotheses are closely entwined (Schluter 2001) because divergent adaptation to disparate niches (1) may result in the formation of isolating barriers (2). In asexual organisms, adaptation to different niches and genetic drift in geographic isolation can occur (1), but reproductive isolation plays no role above the level of individuals because reproductive barriers are already in place. Comparing diversification patterns between the two reproductive modes will enable us to test the two hypotheses regarding why diversity is discontinuous (Maynard Smith and Száthmary 1995; Barraclough and Nee 2001; Barraclough and Herniou 2003; Coyne and Orr 2004).

The two existing comparisons of species discreteness between asexual and sexual clades (i.e., Holman 1987; Fontaneto et al. 2007a) focused on morphological traits. These studies could not satisfactorily determine why discrete entities exist in nature: they lacked a broad sampling regime (Coyne and Orr 2004), they adopted incomparable species concepts historically used for sexual and asexual taxa (de Queiroz 2005), they were qualitative, and they were confounded by different amounts of taxonomic effort between the groups (Fontaneto et al. 2007a). We suggest that a quantitative comparison of patterns of discreteness in genotypes among comparable units of asexual and sexual diversity would make for a more powerful assessment of species discreteness (Barraclough et al. 2003).

Irrespective of their reproductive mode, organisms are expected to diverge into genetic clusters when faced with forces such as geographical isolation or divergent selection, which promote independent evolution between subpopulations (Barraclough et al. 2003). Across a clade, this should result in clusters of closely related individuals separated by longer stem branches (Fig.1). In asexuals, independent evolution occurs because new mutant genotypes in one subpopulation cannot spread and outcompete those in other subpopulations (Templeton 1989; Cohan 2001). By corollary, discrete genetic clusters within a higher clade can be used as a comparable measure of independently evolving groups that represent separate arenas for mutation, selection, and drift (Fisher 1930). These genetic clusters are broadly equivalent to species as defined by the evolutionary (Simpson 1951) or general lineage species concepts (De Queiroz 2007), and constitute entities for which the degree of clustering can be compared between sexual and asexual clades. Although these genetic clusters have been invoked as species in a range of organisms (including asexuals, e.g., Birky et al. 2005; Heethoff et al. 2009; Schön et al. 2012), there has been no statistical comparison of patterns of clustering between related sexual and asexual clades.

**Figure 1:**
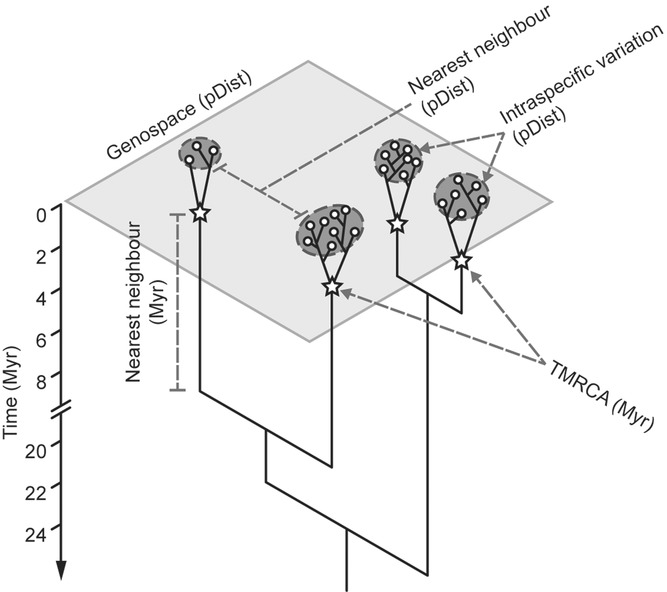
Schematic representation of genospace and how genetic variation corresponds to gene trees. Intraspecific variation can be measured by genetic distance, as delineated by the dark gray dotted ellipses, or time to the most recent common ancestor (TMRCA), as indicated by the stars. Similarly, interspecific variation can be measured using raw genetic distances or phylogenetic distances (long vertical branches). The size of the dotted circle and branch length is relative to the size of the intra- and interspecific variation, respectively. Here phylogenetic distance corresponds to time (Myr) as the trees are chronograms.

Here, we use a densely sampled dataset to survey clustering of a mitochondrial barcoding marker, cytochrome *c* oxidase subunit I (COI), in representative sexual and asexual clades of Rotifera. We delimit genetic clusters, which we refer to as “species” as defined above, using the Generalized Mixed Yule Coalescent model (GMYC—Pons et al. 2006; Fujisawa and Barraclough 2013—see Barraclough et al. 2003 and Fontaneto et al. 2007b for a discussion of whether clusters should be regarded as populations, species, or some other unit of diversity). The method delimits statistically significant genetic clusters indicative of independently evolving lineages (Birky and Barraclough 2009) and provides comparable units that circumvent taxonomic issues that confound Rotifera taxonomy (e.g., Suatoni et al. 2006; Fontaneto et al. 2009). Putative rotifer species delimited using this method have been shown to be both morphologically and ecologically divergent (e.g., Birky et al. 2005; Fontaneto et al. 2007b).

The discreteness of species depends both on intra- and interspecific variation. These in turn are affected by population genetic mechanisms and patterns of net diversification (speciation [λ] minus extinction [μ]), respectively, as well as by sampling. We separate out the following questions

(1) *Are species in asexual clades more or less cohesive than those in sexual clades?* Intraspecific variation, measured as average pairwise genetic distances (π) or the time to most recent common ancestor (TMRCA), reflects the cohesiveness of species. Cohesiveness will depend on the effective population size (*N_e_*) as well as the demographic and selective history of the marker gene (Rosenberg and Nordborg 2002; Charlesworth et al. 2003). Several processes could cause systematic differences in intraspecific variation, potentially in different directions, between sexuals and asexuals. Even if census population sizes (*N*) were similar in sexuals and asexuals, and all other confounding factors were equal, asexual species should have double the effective population size for a mitochondrial marker (mtDNA). This is because all parents in the asexual species pass on mtDNA rather than just half the parents (Lynch and Hill 1986); therefore for asexuals, species clusters will be less genetically cohesive. However, selection might oppose this prediction. In asexual populations, the entire genome is inherited as a single unit unaffected by recombination. Any selective sweep should reduce variation across the entire genome, whereas in sexuals selective sweeps only reduce variation in loci linked to beneficial mutations (Barraclough et al. 2007; Rice and Friberg 2009; Swanstrom et al. 2011). If asexual populations are afflicted by recurring selective sweeps, one would expect lower average genetic variation at marker genes than in sexual populations, even at neutral loci and sites. The balance of these different processes would determine any systematic differences in mtDNA variation between sexuals and asexuals. Finally, as well as these direct effects of reproductive mode, there might be other systematic differences in demography or ecology that could lead to differences in average levels of intraspecific genetic variation (Barraclough et al. 2007).

(2) *Are species in asexual clades more or less divergent from their closest related species than in sexual clades?* Average levels of interspecific divergence depend on the net rate of diversification. Somewhat paradoxically, faster net rates of diversification will tend to reduce the discreteness of species because species will be on average less divergent from their nearest related species. A clade with greater species richness will tend to have shorter distances between species than a clade with fewer species of the same crown age. Reproductive mode could affect diversification in either direction: if speciation is limited by the rate at which reproductive isolation evolves (Felsenstein 1981; Rice and Hostert 1993), then one might expect asexuals to diversify more than sexuals because their diversification does not depend on the evolution of reproductive isolation (Barraclough and Herniou 2003). In contrast, if the rate of adaptation to new ecological niches limits speciation, then one might expect sexuals to diversify more because the greater efficiency of natural selection attributed to recombination (Weismann 1889; Burt 2000; Becks and Agrawal 2012) should allow them to adapt faster to new niches. Preliminary findings from Fontaneto et al. (2012b) indicate that asexual bdelloid rotifers have a faster net diversification rate than their sexual sister clade. Here we extend their analyses: we include four additional genera, more sequences, provide better age estimates, and compare net diversification rates statistically with likelihood ratio tests.

(3) *Do confounding factors affect the comparison of sexuals and asexuals?* The pattern of discreteness in gene trees might be affected by other factors affecting the level of both intra- and interspecific sampling. For example, if sexual and asexual clades differ in their geographical distributions (i.e., more local endemics in sexuals compared to asexuals), then differences in genetic patterns might reflect sampling bias associated with dispersal ability, dormancy, or generation time independent from their reproductive mode.

Rotifera is an ideal phylum to address these questions because it encompasses clades (classes) with a variety of reproductive modes that have survived long enough for speciation (Coyne and Orr 1998; Burt 2000; Butlin 2002). Bdelloid rotifers are obligate asexuals (Mark Welch and Meselson 2009; Birky 2010; Flot et al. 2013), which fossil evidence suggests have persisted without sex for at least 35 million years (Poinar and Ricci 1992). Monogonont rotifers, their potential sister clade (Fontaneto and Jondelius 2011), are cyclical parthenogens with a frequency of recombination high enough to treat them as effectively sexual for macroevolutionary purposes (cf. Tsai et al. 2008).

One complication for the study of diversification patterns is that the two classes vary in their ecology as well as in their sexuality. Differences in dispersal ability, for example, might affect their observed genetic patterns. Bdelloids are aquatic limnoterrestrial microinvertebrates (i.e., inhabit terrestrial environments with an aqueous matrix), and most species can survive desiccation by contracting into a tun; this characteristic attributes them a high capacity for dispersal and subsequent colonization and establishment in novel ephemeral environments (Jenkins and Underwood 1998; Wilson and Sherman 2013). Monogononts occupy a variety of aquatic habitats encompassing a range of salinity but, compared to bdelloids, are less likely to inhabit ephemeral environments as they are less tolerant of desiccation (Ricci 2001). Nonetheless, in bdelloids there is a range in desiccation tolerance (Ricci 1998) with several species restricted to the same habitats as monogononts: by including habitat type as a covariate in our comparisons, we can attempt to disentangle the effect of ecology from reproductive mode (although we cannot rule out other ecological characteristics that might differ between the clades and influence diversification patterns).

We tested for differences in species discreteness between asexual bdelloid and sexual monogonont rotifers, in terms of intra- and interspecific variation (Fig.1), using GMYC species delimited from COI sequence data. We also performed macroevolutionary analyses to compare the net rate of diversification and changes in rate over time (Nee et al. 1994; McPeek 2008). Conclusions from this system will add empirical genetic evidence to existing theoretical (Maynard Smith and Száthmary 1995; Coyne and Orr 2004) and morphological studies (Holman 1987; Fontaneto et al. 2007a) concerning why species are discrete.

## Materials and Methods

### OBTAINING COMPARABLE UNITS OF DIVERSITY

Comparable units of diversity were delimited using the GMYC model with ultrametric phylogenies as input. Because downstream processes required estimates of divergence times, phylogenies were time-calibrated and anchored by substitution rate among the 13 datasets (described in more detail below). In the absence of fossil data and accepted calibration points or substitution rates for Rotifera, a backbone phylogeny was generated to obtain internal calibration nodes. The backbone phylogeny was generated using a concatenated alignment of 18S rDNA and COI mtDNA sequences from a subset of taxa representative of the sampled genera. Using this backbone phylogeny, a combined time-calibrated phylogeny of the specimens was reconstructed using COI and subsequently separated into individual gene trees for each of the 13 species complexes and genera. Detailed methods are as follows.

#### Data collection

COI sequence data were mined from GenBank and supplemented with targeted sequencing. A total of 4211 COI sequences (3659 GenBank; 552 sequenced [Table S1]) were collated from 13 monophyletic groups (herein referred to as datasets, each of which had a minimum 38 sequences) corresponding to genera or complexes of cryptic species (six bdelloid and seven monogonont species complexes or genera; Table S2). The sequences were split into these datasets because we are interested in processes acting at lower taxonomic levels and we lack comprehensive sampling at higher levels or in other genera/species complexes. Additional populations of the genera *Ascomorpha*, *Keratella*, and *Polyarthra* were sequenced following protocols similar to Obertegger et al. (2012). For the backbone phylogeny, 19 18S sequences were downloaded from GenBank and paired with, where possible, COI sequences from the same individual, but otherwise the same species or genera (Table S2). Most of these 18S sequences were produced for a previous study (Tang et al. 2012), the details of which are reported in the Supporting Information. Although these data came from multiple studies, the sampling regime used is standard among rotiferologists (Diego Fontaneto was involved in the collection of 74.5% of the samples; Table S3). Further details of the methods used for specimen collection, sequencing, and concatenation can be found in the Supporting Information.

Differences in dispersal ability associated with habitat preferences might affect the genetic structure of the dataset and, subsequently, measures of discreteness and population genetics. This was factored into the analyses by annotating the sequences by habitat type—limnoterrestrial (limited to bdelloids) or aquatic habitats (typical of monogononts but also of several bdelloid clades; Table S3).

#### Phylogenetic analyses

**Figure 2:**
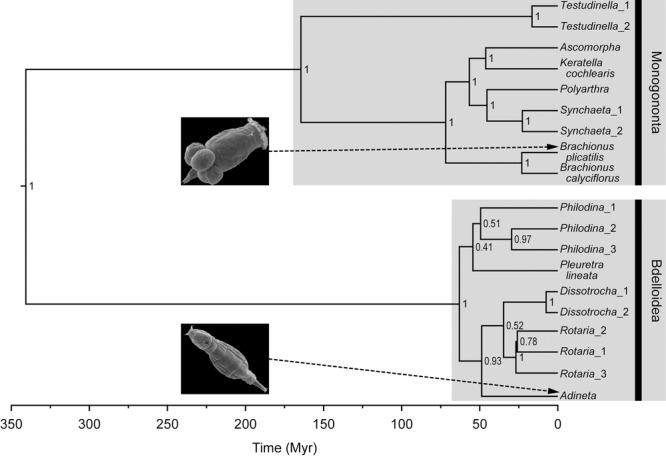
BEAST chronogram for a subset of Bdelloidea and Monogononta inferred using a concatenated alignment of both COI mtDNA and 18S rDNA. The substitution model GTR + Γ + I was found to be the best fit for both COI and 18S. A lognormal relaxed clock with separate substitution rates for COI (1.76% Myr^−1^) and 18S (0.02% Myr^−1^) were used. The posterior probability values are shown as clade support at the nodes. Images of *Brachionus manjavacas* (of the *B. plicatilis* species complex) and *Adineta tuberculosa* were taken by Diego Fontaneto and Giulio Melone.

Phylogenetic methods are visualized in Figure S1. For the backbone phylogeny, all of the 19 COI and 18S sequences were aligned using MAFFT (Katoh et al. 2009) within Geneious Pro version 5.4.2 (Drummond et al. 2006) and checked by eye. A concatenated alignment of 18S and COI was used to reconstruct a time-calibrated phylogeny with BEAST version 1.7.5 (Drummond and Rambaut 2007). The parameters comprised a GTR + Γ + I substitution model (defined using Akaike information criteria in jModelTest 2—Darriba et al. 2012), a relaxed lognormal clock, a birth–death prior (Gernhard 2008), a random starting tree, 100,000,000 generations, and sampling every 1000 generations. Separate calibration clocks for COI (1.76% Myr^−1^; tested for the GTR + Γ + I model in aquatic invertebrates—Wilke et al. 2009) and 18S (0.02% Myr^−1^; as suggested by Ochman and Wilson 1987; Bargues et al. 2000) were used. These calibrations were used in the absence of published Rotifera specific rates; however, we acknowledge that this external rate may lead to the underestimation of intraspecific rates (Ho et al. 2008). The Markov chain Monte Carlo (MCMC) sample was checked for convergence in Tracer version 1.5 (Rambaut and Drummond 2007), and the trees were combined into a maximum credibility tree while keeping the target node heights with a 10% burn-in in TreeAnnotator version 1.7.5 (Fig.2).

To reduce the impact of clade-specific rate heterogeneity, the large COI alignment was analyzed in two combined analyses (instead of 13 individual ones) using the node ages (including the 95% highest posterior densities [HPD] confidence intervals) and sequences from the backbone tree. As dichotomy is a prerequisite for the downstream processes, alignments were collapsed into unique haplotypes using DnaSP version 5 (Librado and Rozas 2009). The COI haplotype alignment was split into two by separating out sister clades (see Supporting Information). Ultrametric trees were generated from these two alignments using the protocol outlined above for the backbone phylogeny except that the node ages of each divergence were specified with soft boundaries corresponding to the 95% HPD confidence intervals. Three independent runs were performed and combined using LogCombiner version 1.7.5 and subsequently summed using TreeAnnotator. These two large combined gene trees were then separated into the 13 individual species complexes and genera gene trees. For each tree, the effect of rate heterogeneity on branch lengths (File S1) and the effect of a combined analysis on diversity estimation were validated (File S2). All phylogenetic analyses were performed on the CIPRES Science Gateway (Miller et al. 2010).

#### Generalized Mixed Yule Coalescent model

The GMYC was used to delimit genetic clusters indicative of independently evolving groups akin to species (Pons et al. 2006; Fujisawa and Barraclough 2013). The GMYC tests for significant shifts in branching rate in an ultrametric tree, which represents a switch from interspecific evolutionary processes to intraspecific population level processes. This is expected if the dataset comprises multiple individuals from a set of independently evolving entities (Birky and Barraclough 2009). A model encompassing two different branching rates is fitted and assessed against the data, and a χ^2^ test is performed to gauge the significance of the GMYC model fit against the null hypothesis (i.e., a single coalescent with one branching rate). If the χ^2^ test is significant, the threshold is used to delimit species on the gene tree. This method has been used to identify independently evolving bdelloid (e.g., Fontaneto et al. 2007b, 2012; Birky and Barraclough 2009) and monogonont rotifers (e.g., Obertegger et al. 2012; Leasi et al. 2013; Malekzadeh-Viayeh et al. 2014), and produces clusters that are congruent with other species delimitation methods (Tang et al. 2012). The analysis was performed with the splits 1.0–11 package (Ezard et al. 2009—available from https://r-forge.r-project.org/projects/splits) in R 2.15.2 (R Core Team 2012). The effect of sample size on GMYC supports values and how that differs between bdelloid and monogonont datasets was assessed (File S3).

### PATTERNS OF GENETIC DISCRETENESS

#### Discreteness measures

The discreteness of the genetic clusters was assessed in terms of intra- and interspecific phylogenetic distances (branch lengths equivalent to age on a time-calibrated tree) and raw genetic distances (Fig.1). Two alternative metrics of intraspecific variation were calculated: the TMRCA and the average raw nucleotide diversity within a cluster (π; Nei and Li 1979). These two measures are concerned with different aspects of the data and are affected differently by variable sample sizes: TMRCA is more directly relevant for species clustering but is strongly affected by small sample sizes leading to underestimation, while π accounts for sample size. TMRCA was measured using the basal node age of each GMYC species on the ultrametric tree using the ape 3.0.5 package (Paradis et al. 2004) in R. Interspecific genetic divergence was calculated as the minimum phylogenetic distance (divergence time) and the minimum raw genetic distance to a heterospecific. Minimum phylogenetic distance between GMYC species was obtained from edge lengths on the trees, and this total branch length was halved to obtain divergence time. Raw intra- and interspecific pairwise distances were calculated in MEGA version 5 (Tamura et al. 2011).

#### Population genetic signatures

Neutrality tests were conducted for each GMYC species in DnaSP, and their signatures compared between bdelloids and monogononts to test whether differences in the frequency of demographic and selection processes affect intraspecific variation. It is important to note that we cannot firmly distinguish selection and demographic processes from single locus data alone, but we can test whether there is a systematic difference in the pattern of genetic variation consistent with our predictions based on selection and demography. Five related population genetic neutrality signatures were estimated: D* (Fu and Li 1993), F* (Fu and Li 1993), *F*_S_ (Fu 1997), Tajima's *D* (Tajima 1989), and *R*_2_ (Ramos-Onsins and Rozas 2002). These tests are closely related but differ in their ability to detect shifts from neutrality, namely bottlenecks, selective sweeps, hitchhiking, and opposing signatures of selection (Ramos-Onsins and Rozas 2002). For each of the test statistics, a value close to zero indicates neutrality, a positive value indicates either balancing selection at the locus or a recent population decrease, whereas a negative value indicates population expansion, genetic hitchhiking, or purifying selection at the locus.

### PATTERNS OF NET DIVERSIFICATION

Investigating the net diversification rate (i.e., speciation rate [λ] minus extinction rate [μ]) and the constancy of net diversification over time (i.e., departure from a constant diversification rate model) requires ultrametric trees with branch lengths and tips corresponding to time and species, respectively. Therefore, for each dataset the ultrametric tree was pruned so that each tip represented a GMYC species. The retained tip was the one with the longest sequence length.

#### Net diversification rate

Net diversification rate was independently estimated for bdelloids and monogononts using a modified version of the ape *birthdeath* function; with the modified version, the λ and μ of pooled data (e.g., bdelloids, monogononts, or Rotifera) can be assessed simultaneously (Supporting Information). A log-likelihood statistic was used to validate the separate parameterization of λ and μ for bdelloids and monogononts (four parameters) over a “global” model with a single λ and μ estimated across all of the data (two parameters).

#### Constancy of net diversification

The constancy of net diversification of each dataset was tested using the γ statistic of Pybus and Harvey (2000) within the laser 2.3 package (Rabosky 2006) in R. The analysis followed Fontaneto et al. (2012) but includes additional rotifer taxa. The γ statistic tests the constancy of per-lineage net diversification rates over time by comparing relative positions of internal nodes within a phylogeny to expected positions generated by a constant-rate model. Positive γ values signify either recent increases in net diversification rate or high background extinction rates (Barraclough and Nee 2001). Negative γ values indicate either early net diversification followed by deceleration (expected with density-dependent net diversification such as ecological niche filling—McPeek 2008) or incomplete species sampling (Barraclough and Nee 2001; Rabosky and Lovette 2008). We account for the effect of incomplete sampling in two ways: (1) an a priori correction was performed using a missing species simulator (CorSiM—Cusimano et al. 2012) based on a constant rate birth–death model (within TreePar 2.5—Stadler 2011) and (2) a post hoc correction performed using a Monte Carlo constant rates test (MCCR within laser—Pybus and Harvey 2000). Further details can be found in the Supporting Information.

### DATA ANALYSIS

Statistical analyses were performed in R to identify whether reproductive mode explains differences in species discreteness, sample sizes per species, and patterns of net diversification. To identify whether species discreteness measures are affected by reproductive mode (asexuality vs. sexuality) or habitat type (limnoterrestrial vs. aquatic), general linear mixed effects models (LMEMs) with a Gaussian error structure and an identity link function were implemented in the lme4 0.999999.0 package (Bates and Sarkar 2007). Response variables included (1) π, (2) TMRCA, the nearest heterospecific neighbor in terms of (3) genetic distance and (4) phylogenetic distance, and (5) various population genetic signatures (D*, F*, *F*_S_, Tajima's *D*, and *R*_2_). Reproductive mode and habitat type were included into the model as fixed effects. To account for potential disproportionate influence of few taxa (i.e., taxonomic pseudoreplication), morphospecies nested within genus and number of sequences per GMYC species were blocked out as random effects. The nested random effects (i.e., morphospecies within genus) made LMEMs appropriate. MCMC *P* values (pMCMC) with HPD confidence intervals for the parameters of each model were estimated using 10,000 samples within the languageR 1.4 package (Baayen et al. 2008).

The difference in the number of sequences and unique haplotypes per GMYC cluster, the proportion of singleton taxa per dataset, and the constancy of net diversification between bdelloids and monogononts, estimated independently for γ-corrected a priori and post hoc, were assessed using Wilcoxon rank sum tests. This test was appropriate due to the lack of random effects and because each dataset has a single measure for each of those variables.

Retrospective power analyses were performed to determine whether any lack of significance was due to inadequate sample size or insufficient power. Power is the proportion of times the null hypothesis is rejected when it is false; this was calculated from sample sizes, significance level (α), and effect sizes (Cohen's *d*) using the pwr 1.1.1 package (Champely and Champely 2007). Cohen's *d* effect size provides an indication of the strength of the focal relationship, with higher values indicative of a stronger relationship; these were calculated by dividing the group means (i.e., asexual or sexual) by the pooled standard deviation (Cohen 1992).

## Results

The 13 datasets contained between 38 and 1541 sequences and yielded 5–120 GMYC species. The ratio of species estimated using the GMYC compared to traditional taxonomy ranged from 2:1 to 77:1 (on average 16 times higher; Table S2). On average, bdelloid species had fewer sequences (5.63 ± 1.56 and 16.4 ± 3.32, bdelloid and monogonont, respectively) and unique haplotypes per GMYC cluster (1.75 ± 0.18 and 4.42 ± 1.1, respectively) than the monogonont species (Wilcox_sequences_: *W* = 3, *P* = 0.0082; Wilcox_haplotypes_: *W* = 1, *P* = 0.0053, respectively). As a result, the transition from inter- to intraspecific branching used by the GMYC to detect clusters was qualitatively steeper in monogononts than in bdelloids (average GMYC *P* values: 0.0081 vs. 0.014; Table S2) and indicative of a more saturated sample of haplotypes (File S3). Furthermore, the proportion of singletons was higher in the bdelloid clades (63.4 ± 4.05% in bdelloids; 40.97 ± 5.17% in monogononts; Wilcox_singletons_: *W* = 144, *P* = 0.0022). Singletons can either consist of multiple clonal samples (collapsed into a single haplotype) or a single-sample specimen; the proportion of single-sample specimens was similar between bdelloids (37%) and monogononts (39%).

### DISCRETENESS MEASURES

**Figure 3:**
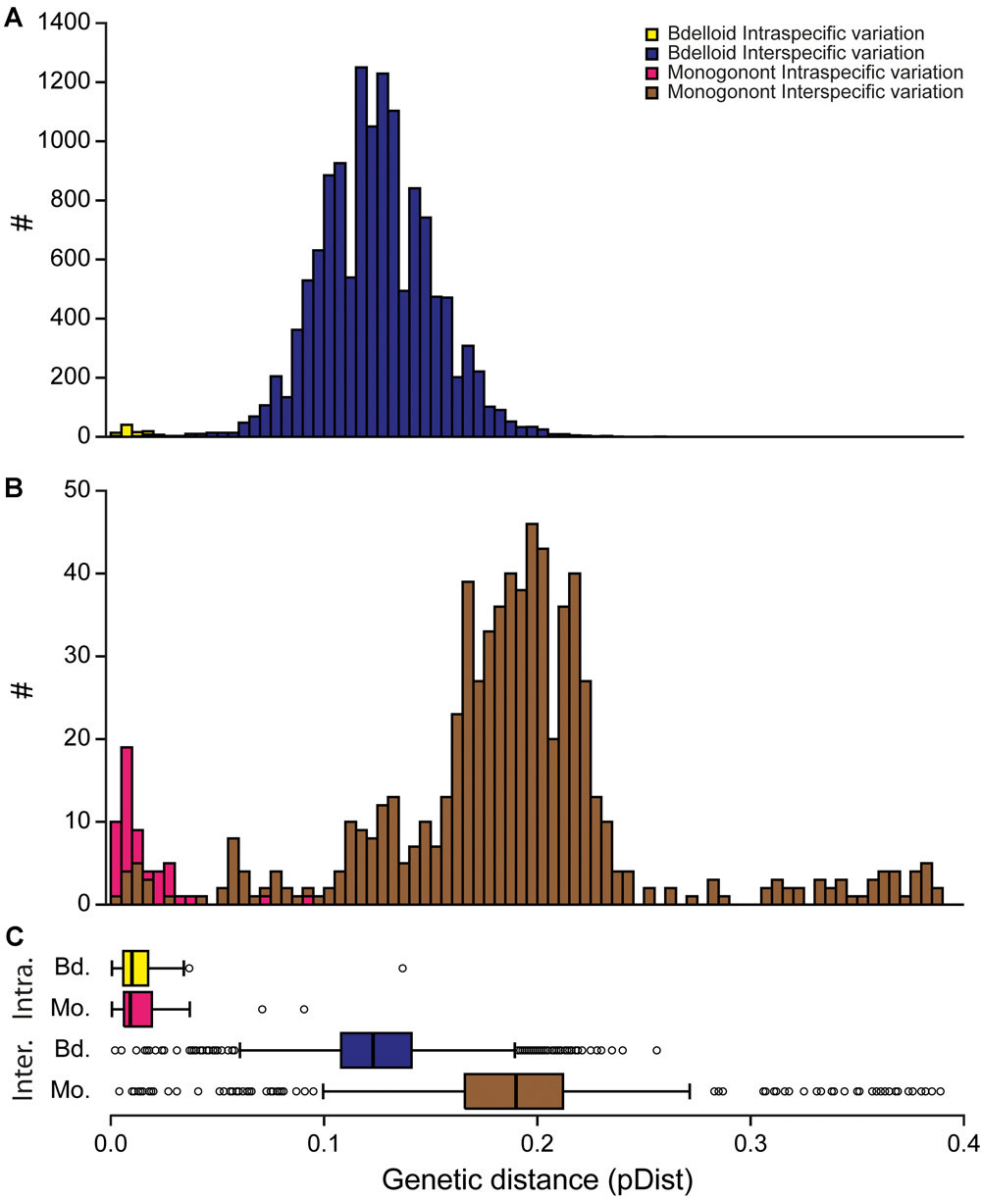
Distribution of all the intra- and interspecific raw pairwise genetic distances for bdelloid and monogonont rotifers and the differences in the two groups for intra- and interspecific genetic distance. The distribution of genetic distances for bdelloid (A) and monogonont (B) GMYC species delimited using Bayesian gene trees is shown. The box plots highlight the differences between bdelloid and monogonont rotifers for intra-and interspecific genetic distance (C). The small open circles represent outliers.

**Figure 4:**
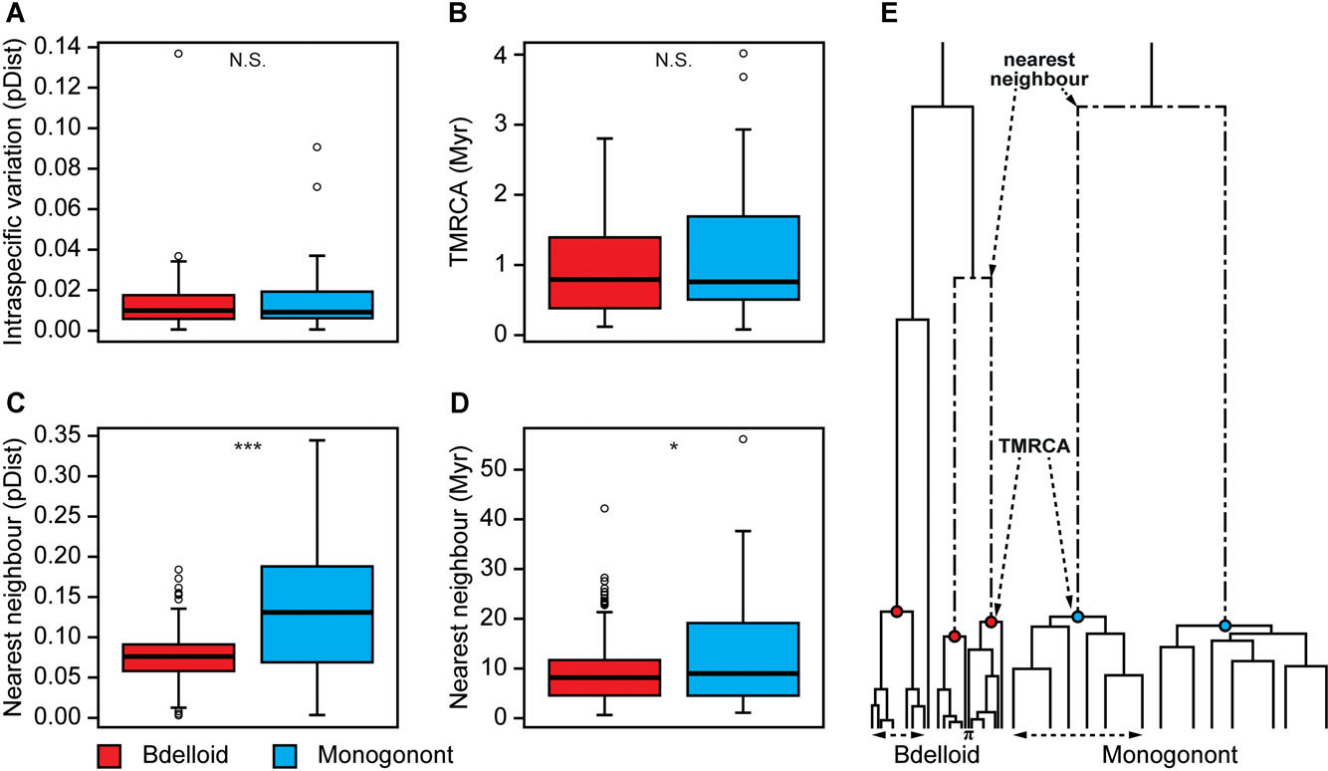
Box plots representing the discreteness of bdelloid (light gray; red online) and monogonont (dark gray; blue online) rotifer GMYC species in relation to intraspecific genetic distance (π [pDist]; A), time to the most recent common ancestor (TMRCA [Myr]; B), genetic distance to the nearest heterospecific neighbor (pDist; C), and phylogenetic distance to the nearest heterospecific neighbor (Myr; D). (E) Schematic of the typical bdelloid and monogonont gene trees; species are represented by nodes at the point of coalescence. The dashed arrows highlight aspects of the phylogeny used to measure nearest neighbor (D), TMRCA (B), and pi (Π; A). For the box plots, measurements were averaged across bdelloid and monogonont Bayesian datasets. Open circles represent outlier values. The significance of the difference between bdelloid and monogonont datasets for the various measures is shown at the top of each box (N.S. = *P* > 0.05; *P < 0.05; **P < 0.01; ***P < 0.001).

Interspecific divergence (raw and phylogenetic distance) was significantly greater in monogononts than in bdelloids (Table1; Figs.3, 4); in contrast, intraspecific variation (π or TMRCA) was similar between the two classes. The pattern of clustering was consequently more discrete in monogonont rotifers. These differences in discreteness were not attributed to habitat type (aquatic vs. limnoterrestrial; Table1) and remained significant when the number of haplotypes per GMYC species, dataset, and morphospecies were blocked out as random effects.

**Table 1:** Results of general linear mixed effects models (LMEMs) for the discreteness of species (intraspecific variation [π: pDist], time to most recent common ancestor [TMRCA: Myr], nearest neighbor [pDist], and nearest neighbor [Myr], analyzed separately)

Response	Explanatory (fixed/*random*)	MCMC_mean_ /*Variance*	HPD(±)/*SD*	LR χ^2^	*P*
Intraspecific variation (pDist)	**Asexual aquatic (intercept)**	**0.013**	**0.0057, 0.022**	**−**	**0.0016**
	**Sexual**	**0.0012**	**−0.0089, 0.01**	**−**	**0.79**
	**Limnoterrestrial**	**−0.0004**	**−0.0095, 0.0082**	**−**	**0.95**
	*Morphospecies identity*	*0*	*0*	*0*	*0*
	*Sample size*	*6.26 × 10^−6^*	*0.0025*	−*1.18*	*0*
	*Dataset*	*3.16 × 10^−5^*	*0.0056*	−*4*	*0*
	*Residual*	*0.0002*	*0.014*		
TMRCA (patristic)	**Asexual aquatic (intercept)**	**1.13**	**0.61, 1.64**	**−**	**0.0001**
	**Sexual**	**0.16**	**−0.41, 0.71**	**−**	**0.56**
	**Limnoterrestrial**	**0.33**	**−0.15, 0.76**	**−**	**0.16**
	*Morphospecies identity*	*4.21 × 10^−17^*	*6.49 × 10^−9^*	*0*	*0*
	*Sample size*	*0.15*	*0.39*	−*18.12*	*0*
	*Dataset*	*0.23*	*0.48*	−*2.16*	*0*
	*Residual*	*0.38*	*0.62*		
Nearest neighbor (pDist)	**Asexual aquatic (intercept)**	**0.076**	**0.048, 0.1**	**−**	**0.0001**
	**Sexual**	**0.069**	**0.033, 0.1**	**−**	**0.0001**
	**Limnoterrestrial**	**−0.0031**	**−0.024, 0.016**	**−**	**0.76**
	*Morphospecies identity*	*0.00065*	*0.026*	−*26.48*	*0*
	*Sample size*	*0*	*0*	*0*	*0*
	*Dataset*	*0.0015*	*0.038*	−*1.42*	*0*
	*Residual*	*0.0012*	*0.035*		
Nearest neighbor (patristic)	**Asexual aquatic (intercept)**	**8.27**	**3.94, 11.94**	**−**	**0.001**
	**Sexual**	**5.86**	**0.72, 11.02**	**−**	**0.023**
	**Limnoterrestrial**	**0.32**	**−2.77, 3.4**	**−**	**0.83**
	*Morphospecies identity*	*17.83*	*4.22*	−*16.4*	*0*
	*Sample size*	*0*	*0*	*0*	*0*
	*Dataset*	*22.36*	*4.73*	−*1.2*	*0*
	*Residual*	*34.24*	*5.85*		

Reproductive mode and habitat type were included as fixed effects (bold), whereas morphospecies identity, sample size (no. of haplotypes per GMYC species), and the focal dataset were included as random effects (italics). *P* values of fixed effects on the HPD intervals obtained from Markov Chain Monte Carlo (MCMC) sampling and *P* values of random effects are based on likelihood ratio tests (LR χ^2^).

### POPULATION GENETIC SIGNATURES

**Figure 5:**
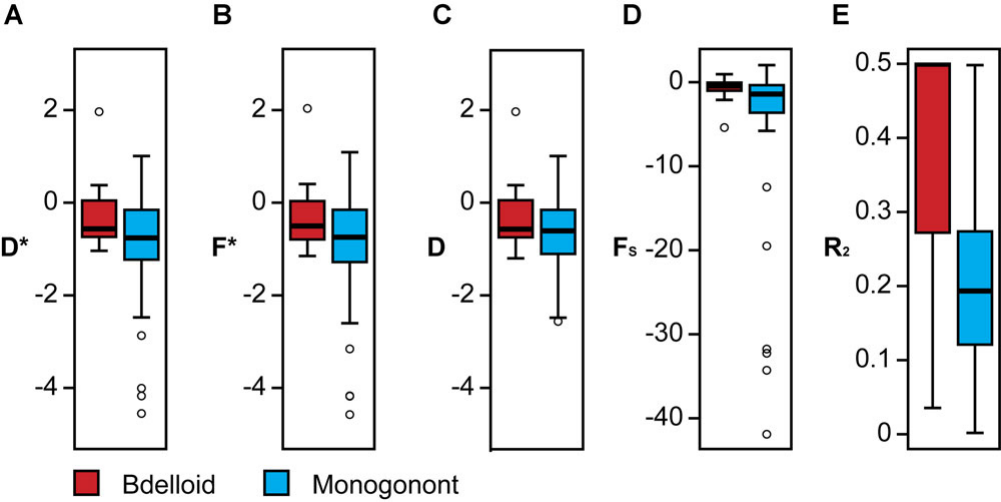
Box plots showing the distribution of five population genetics signatures across clusters within both bdelloid (light gray; red online) and monogonont (dark gray; blue online) rotifers. Boxes indicate the extent of the first and third quartiles. Whiskers indicate the most extreme datapoints within 1.5 times the interquartile distance from the box and open circles represent outlier values. (A) D*; (B) F*; (C) *F*_S_; (D) Tajima's *D*; (E) *R*_2_.

None of the five population genetic signatures differed significantly between bdelloid and monogonont rotifers or habitat types (Table2; Fig.5), although monogononts exhibited qualitatively more negative estimates for each of the population genetic signatures (Table2).

**Table 2:** Results of general linear mixed effects models (LMEMs) assessing whether results of the neutrality tests (D^*^, F^*^, *F*_S_, *D*, and *R*_2_, analyzed separately) were explained by reproductive mode and/or habitat type

Response	Explanatory (fixed/*random*)	MCMC_mean_ /*Variance*	HPD(±) /*SD*	LR χ^2^	*P*
D^*^	**Asexual aquatic (intercept)**	**−0.27**	**−1.41, 0.95**	**-**	**0.63**
	**Sexual**	**−0.64**	**−1.75, 0.51**	**-**	**0.25**
	**Limnoterrestrial**	**−0.63**	**−1.86, 0.52**	**-**	**0.28**
	*Morphospecies identity*	*0*	*7.13 × 10^−7^*	*0*	*0*
	*Sample size*	*1.68*	*1.3*	−*13.05*	*0*
	*Dataset*	*0.12*	*0.35*	−*0.084*	*0*
	*Residual*	*0.48*	*0.69*		
F^*^	**Asexual aquatic (intercept)**	**−0.27**	**−1.47, 0.96**	**-**	**0.65**
	**Sexual**	**−0.68**	**−1.86, 0.48**	**-**	**0.24**
	**Limnoterrestrial**	**−0.64**	**−1.87, 0.62**	**-**	**0.3**
	*Morphospecies identity*	*2.80 × 10^−11^*	*5.29 × 10^−6^*	*0*	*0*
	*Sample size*	*1.68*	*1.3*	−*11.68*	*0*
	*Dataset*	*0.14*	*0.37*	−*0.056*	*0*
	*Residual*	*0.54*	*0.74*		
*F*_S_	**Asexual aquatic (intercept)**	**−6.66**	**−14.09, 0.8**	**-**	**0.088**
	**Sexual**	**−2.94**	**−9.96, 3.65**	**-**	**0.39**
	**Limnoterrestrial**	**−0.41**	**−8.23, 6.65**	**-**	**0.91**
	*Morphospecies identity*	*0*	*0*	*0*	*0*
	*Sample size*	*228.38*	*15.11*	−*158.42*	*0*
	*Dataset*	*0.48*	*0.69*	−*2.58*	*0*
	*Residual*	*0.72*	*0.85*		
*D*	**Asexual aquatic (intercept)**	**0.011**	**−0.9, 0.84**	**-**	**0.97**
	**Sexual**	**−0.59**	**−1.47, 0.35**	**-**	**0.19**
	**Limnoterrestrial**	**−0.66**	**−1.64, 0.3**	**-**	**0.16**
	*Morphospecies identity*	*3.21 × 10^−28^*	*1.79 × 10^−14^*	*0*	*0*
	*Sample size*	*0*	*0*	*0*	*0*
	*Dataset*	*0.24*	*0.49*	−*0.22*	*0*
	*Residual*	*0.56*	*0.75*		
*R*_2_	**Asexual aquatic (intercept)**	**0.37**	**0.18, 0.56**	**-**	**0.001**
	**Sexual**	**−0.17**	**−0.36, 0.029**	**-**	**0.089**
	**Limnoterrestrial**	**−0.1**	**−0.27, 0.069**	**-**	**0.25**
	*Morphospecies identity*	*0*	*0*	*0*	*0*
	*Sample size*	*0.015*	*0.12*	−*8.27*	*0*
	*Dataset*	*2.22 × 10^−12^*	*1.49 × 10^−6^*	*0*	*0*
	*Residual*	*0.14*	*0.38*		

Reproductive mode and habitat type were included as fixed effects (bold), whereas morphospecies identity, sample size (no. of haplotypes per GMYC species), and the focal dataset were included as random effects (italics). *P* values of fixed effects on the HPD intervals obtained from Markov Chain Monte Carlo (MCMC) sampling and *P* values of random effects are based on likelihood ratio tests (LR χ^2^).

### PATTERNS OF NET DIVERSIFICATION

**Figure 6:**
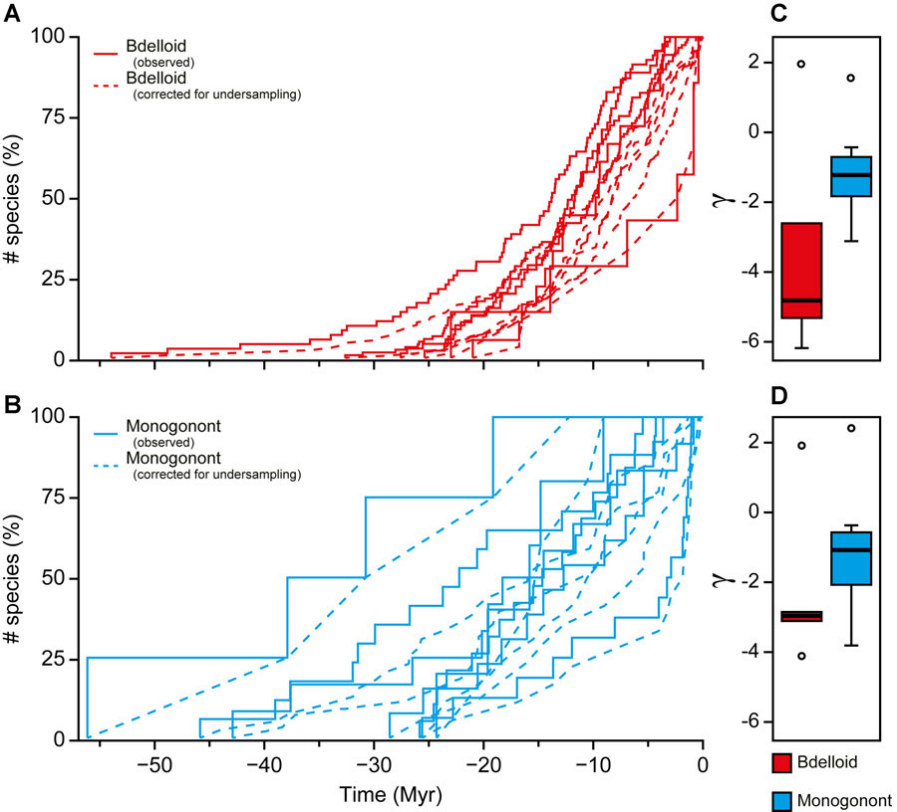
Lineage through time plots of bdelloid (light gray; red online) and monogonont rotifers (dark gray; blue online) on absolute time scales with differences in shifts in diversification rate (γ) shown. Lineage through time plots (bdelloid (A) and monogonont (B)) are based on pruned Bayesian trees, each tip represents only one terminal per species as identified by the GMYC. Lineage through time plots of phylogenies corrected for undersampling using CorSiM are also shown (dotted lines). Differences between bdelloids and monogononts in the distribution of the raw (C) and corrected values for the γ statistic (D) are shown. Open circles represent outlier values.

Phylogenetic analyses of this sample indicated that bdelloids and monogononts are both monophyletic. The crown age of the bdelloids was found to be 58.16 Myr (HPD 95%—34.84–99.99; Fig.2), a finding congruent with the available fossil evidence. Bdelloids had a significantly faster net diversification rate (0.072 ± 0.003) than monogononts (0.048 ± 0.004; Table3). Separate parameterization for both bdelloid and monogonont clades produced a better model fit than modeling all the data together (χ^2^ = 11.26, *P* = 0.018; Table3). All the bdelloid clades (except *Pleuretra*) exhibited decelerating net diversification rates with negative values for the γ statistic; these γ values remained significantly negative despite incomplete sampling (corrected for using either CorSiM or the MCCR test; Fig.6; Table S2). In contrast, only one monogonont dataset (*Brachionus plicatilis*) remained significantly negative when sampling biases were corrected for (Table S2). Monogonont clades typically exhibited more constant net diversification rates compared to bdelloid clades, this was indicated by more positive γ values (−1.13 ± 0.55 vs. −3.63 ± 1.22; Table4; Fig.6). The difference in rate slowdowns between monogononts and bdelloids was not significant, this is likely due to the insufficient power at this significance level (0.05), effect size (determined by Cohen's *d*: 0.98), and sample size (power analysis: effect size = 1.58, power = 0.36).

**Table 3:** Estimation of net diversification rates (speciation [λ] minus extinction [μ]) from separately parameterized pooled bdelloid, monogonont, and total datasets

Phylogenetic method	Dataset	Diversification rate (λ − μ)	Standard error	No. of tips	Deviance	Log-likelihood	*P* compared to “All”
BEAST	Bdelloid	0.072	0.003	334	168.70	−84.35	0
BEAST	Monogonont	0.048	0.004	93	329.06	−164.53	0
BEAST	All rotifers	0.066	0.002	427	509.02	−254.51	NA

**Table 4:** γ Statistic for both bdelloid and monogonont rotifers and significance of their comparison

	Bdelloid γ_mean_	Bdelloid γ_SE_	Monogonont γ_mean_	Monogonont γ_SE_	*W*	*P*
γ (MCCR)	−3.63	1.22	−1.13	0.55	8	0.073
γ (CorSiM)	−2.22	0.86	−1.01	0.72	10	0.14

Incomplete sampling was addressed using MCCR tests and CorSiM.

## Discussion

Both bdelloid and monogonont rotifers display significant clustering of mitochondrial DNA variation, as expected if both groups had diversified into multiple, independently evolving species. The shift from slow to fast branching rates used to delimit GMYC genetic clusters, however, is steeper in monogononts than in bdelloids. Monogonont clusters are separated by significantly larger distances than bdelloid clusters, and tend to contain more sequences per cluster for equivalently sampled clades: a higher proportion of the sampled sequences group together into clusters. We discuss how this observed difference in discreteness could be ascribed to differences in reproductive mode, ecology, or sampling, and formulate a framework with which these hypotheses could be separated.

The higher discreteness observed in monogononts relative to bdelloids is not due to differences in intraspecific variation. Although there were several theoretical reasons to predict systematic differences (e.g., because bdelloids might have double the effective population size of mtDNA or stronger effects of recurring selective sweeps), average measures of intraspecific variation and neutrality were similar in bdelloids and monogononts. Strong differences in population genetic signatures between the clades might have been obscured by the opposing effects of alternative mechanisms. Future surveys with additional markers would help to investigate these effects further.

Instead, the greater discreteness of monogonont species, compared to bdelloid species, was due to wider genetic gaps between clusters. This pattern is associated with faster net diversification rates in bdelloids than in monogononts, because faster rates lead to species that tend to be more closely related to their nearest relatives. One possible explanation is that, as predicted in the Introduction, bdelloids are better able to take advantage of ecological opportunities for speciation because their diversification does not depend on the evolution of reproductive isolation that would otherwise breakdown the early stages of speciation (Barraclough et al. 2003). Conversely, slower diversification rates in monogononts would be expected if the need to evolve reproductive isolation limits speciation in sexuals (Felsenstein 1985).

There could be other confounding differences between bdelloids and monogononts that explain the observed patterns. One is that greater interspecific distances might be an artifact of differences in sampling. The monogonont sample might have more missing species, which would lead to artificially larger interspecific distances. For example, if monogonont species are more locally endemic than bdelloid species, then geographically restricted sampling might miss more species. We used three different approaches to assess the effect of sampling as a confounding factor: factoring out habitat type, constancy of net diversification rate, and jackknife analyses of GMYC entities. Both bdelloid and monogonont rotifers can disperse by water, wind, or as commensals, and thus have a high dispersal capacity (Wilson and Sherman 2013; Walsh et al. 2014) and broad distributions (Gómez et al. 2002; Segers 2007; Fontaneto et al. 2008; Malekzadeh-Viayeh et al. 2014). Monogonont rotifers, however, exhibit geographical structuring owing to strong founder effects, locally adapted genotypes, and large resting egg banks that buffer against subsequent invasion (monopolization hypothesis—De Meester et al. 2002). Bdelloids do not have large resting egg banks and might therefore have lower endemism owing to their ability to survive desiccation as adults (Ricci 2001) and thus colonize a wide distribution of habitats. However, aquatic bdelloids are much less capable of surviving desiccation (Ricci 1998) and therefore should exhibit similar dispersal patterns to the monogononts they cohabit with. We used habitat type as a proxy for dispersal and colonization capability to address this possibility, reasoning that species of deeper water bodies are less prone to passive dispersal via wind and have lower desiccation tolerance (as shown for certain bdelloid species—Ricci 1998). Interspecific variation was explained by reproductive mode rather than habitat type indicating that differences in clustering between bdelloids and monogononts are not explained by differences in habitat type. It remains possible, however, that other facets of rotifer biology, which affect either true diversification patterns or species sampling, cannot be accounted for by this coarse measure.

As an independent assessment of species sampling, we compared the shapes of species trees. Undersampling of species tends to lead to an apparent slowdown in the net diversification rate toward the present in species trees (Barraclough and Nee 2001) and lower observed net diversification rate (Pybus and Harvey 2000; Ricklefs 2007). In fact, bdelloid but not monogonont trees exhibited deceleration in net diversification rate, which is indicative of either actual diversity-dependent diversification or incompleteness of sampling (Pybus and Harvey 2000; Rabosky and Lovette 2008). If our bdelloid dataset contained a smaller species sample than the monogonont one, then supplementary sampling would further increase bdelloids’ net diversification rates and strengthen our findings.

The final feature relevant to sampling is that there are fewer monogonont clusters but they contain on average three times as many sampled individuals than in bdelloid clusters. This difference indicates that there is a tendency for total genetic diversity to quickly saturate in monogonont samples (as shown by Swanstrom et al. 2011): additional sequences supplement closely related genotypes within species clusters, while maintaining the interspecific gaps. In contrast, further sampling of bdelloids is more likely to continue the discovery of additional divergent haplotypes, acting to fill interspecific gaps. The jackknifing of the bdelloid and monogonont datasets (File S3) resulted in a steeper deterioration in GMYC model fits for bdelloids than for monogononts. This indicates that removing bdelloid sequences tends to underrepresent the coalescent part of the tree and thus reduce the GMYC model fit. In contrast, removal of 50% of the monogonont sequences has less effect on the model fits. Monogonont diversity in these samples was therefore more saturated than the bdelloids, the corollary being that increased monogonont sampling will tend to supplement existing haplotypes and therefore not affect the interspecific gaps or overall diversification rate.

Although we believe that sampling does not explain the observed differences between bdelloids and monogononts, we cannot firmly rule out that possibility. For example, our analyses could not assess whether potential incomplete sampling was randomly dispersed with respect to species. The lack of geographically restricted clades for focused sampling and current underestimation of diversity levels in both clades (e.g., Tang et al. 2012) means that it is hard to envisage a targeted sampling scheme that could improve greatly on our opportunistic one. One alternative would be to focus on co-occurring species in local habitat patches (e.g., ponds) to compare patterns of discreteness of sympatric forms. For example, using environmental metabarcode approaches could allow surveys across multiple habitat patches (e.g., Robeson et al. 2011). Furthermore, our study looks at a single measure of genetic variation, namely variation in one mitochondrial marker. A single marker metasurvey was necessary to encompass the breadth of individual and taxon sampling needed for this study, but cannot capture the entire speciation process and changes in biologically interesting traits (Rokas et al. 2003). For this, an integrative, multilocus, or candidate gene approach is necessary (see Blair and Murphy 2011). Variation among rotifer species for jaw morphology, for example, has been attributed to food particle size preference (monogononts—Ciros-Perez 2001; hypothesized in bdelloids—Melone and Fontaneto 2005). However, even with recent technological advances in sequencing, identifying the genes underlying these traits would remain a challenging task across the scale of samples included here.

Returning to the original question of whether asexual species are as discrete as sexual species: if discrete niches in the environment explained species, we would expect the same level of discreteness in both sexuals and asexuals assuming both were able to adapt to those niches (Barraclough et al. 2003), and this is not observed. Hypothetically, if the environment were continuous and there was no geographical isolation, selection would not create genetic gaps in asexuals, rather there would be increasing divergence as lineages adapted to increasingly divided partitioning of resources (Roughgarden 1979). In sexuals, however, recombination would act as a cohesive force: organisms with similar genotypes are reproductively compatible and only rarely do mechanisms arise to permit reproductive isolation. Of course isolation by distance cannot be ignored and might well be different between bdelloids and monogononts. However, the ubiquity and intercontinental dispersal of both monogonont (Malekzadeh-Viayeh et al. 2014) and bdelloid (Fontaneto et al. 2008) haplotypes makes isolation by distance an unlikely primarily mechanism for the difference in discreteness observed between bdelloid and monogonont species.

Our analysis provides the first genetic evidence that monogonont rotifer species are more discrete than bdelloid rotifer species. Multilocus sequencing of cohabiting bdelloid and monogonont specimens would enable one to narrow down this pattern to either differences in reproductive mode or ecology. Additional targeted sequencing of bdelloid specimens would also help in identifying whether the perceived patterns are due to a lower representativeness of the bdelloid datasets. We posit that difference in sampling is an unlikely explanation given the global sample of bdelloid taxa present here but this needs to be explicitly tested. If we can confirm that the difference in reproductive mode is key to species discreteness, then the results will indicate that while asexuals do speciate into distinct clusters, potentially mediated by adaptation to distinct niches and/or geographical isolation, reproductive isolation is a stronger driver for species discreteness.

## Supporting Information

Table S1 . Specimen collection information and accessions numbers for the sequences generated for this study. **Table S2**. Summary information for each of the 13 datasets, including number of sequences, number of unique haplotypes, estimated diversity, constancy of diversification statistics, estimated ages, and accessions. **Table S3**. Intra- and interspecific diversity measures (genetic and phylogenetic distances) for each of the delimited GMYC entities. **Table S4**. Datasets were split up by sister clades as determined using the backbone phylogeny (Fig.2) and outgroup taxa were added to balance the sequence numbers for each alignment. **Table S5**. Output from GLMM analysis of GMYC model fit (*P* value) differences between bdelloid and monogonont rotifers and varying degrees of jackknifing. **Figure S1**. Phylogenetic methods workflow. **Figure S2**. Minimum interspecific genetic distance (raw pDistance) against minimum phylogenetic distance to the nearest neighbor (Myr). **Figure S3**. Species richness of each of the 13 rotifer datasets analyzed by GMYC but with different input ultrametric trees. **Figure S4**. Significance of the GMYC model fit when bdelloid (red) and monogonont (blue) trees are jackknifed by 20%, 25%, 33%, and 50%. **File S1**. Are the ultrametric trees robust to rate heterogeneity? **File S2**. How does phylogenetic reconstruction method affect species delimitation? **File S3**. Is sampling effort differentially affecting bdelloid and monogonont diversity estimates?
