# Supplementary material for: SEXUAL SPECIES ARE SEPARATED BY LARGER GENETIC GAPS THAN ASEXUAL SPECIES IN ROTIFERS
Source: Evolution. 2014 Jul 25;68(10):2901–16. doi: 10.1111/evo.12483 (PMC4262011; doi:10.1111/evo.12483)
Supplement: Table S1 — . Specimen collection information and accessions numbers for the sequences generated for this study. Table S2. Summary information for each of the 13 datasets, including number of sequences, number of unique haplotypes, estimated diversity, constancy of diversification statistics, estimated ages, and accessions. Table S3. Intra- and interspecific diversity measures (genetic and phylogenetic distances) for each of the delimited GMYC entities. Table S4. Datasets were split up by sister clades as determined using the backbone phylogeny (Fig.2) and outgroup taxa were added to balance the sequence numbers for each alignment. Table S5. Output from GLMM analysis of GMYC model fit (P value) differences between bdelloid and monogonont rotifers and varying degrees of jackknifing. Figure S1. Phylogenetic methods workflow. Figure S2. Minimum interspecific genetic distance (raw pDistance) against minimum phylogenetic distance to the nearest neighbor (Myr). Figure S3. Species richness of each of the 13 rotifer datasets analyzed by GMYC but with different input ultrametric trees. Figure S4. Significance of the GMYC model fit when bdelloid (red) and monogonont (blue) trees are jackknifed by 20%, 25%, 33%, and 50%. File S1. Are the ultrametric trees robust to rate heterogeneity? File S2. How does phylogenetic reconstruction method affect species delimitation? File S3. Is sampling effort differentially affecting bdelloid and monogonont diversity estimates? [file evo0068-2901-SD1.zip › evo12483-sup-0001-figureS1.pdf]

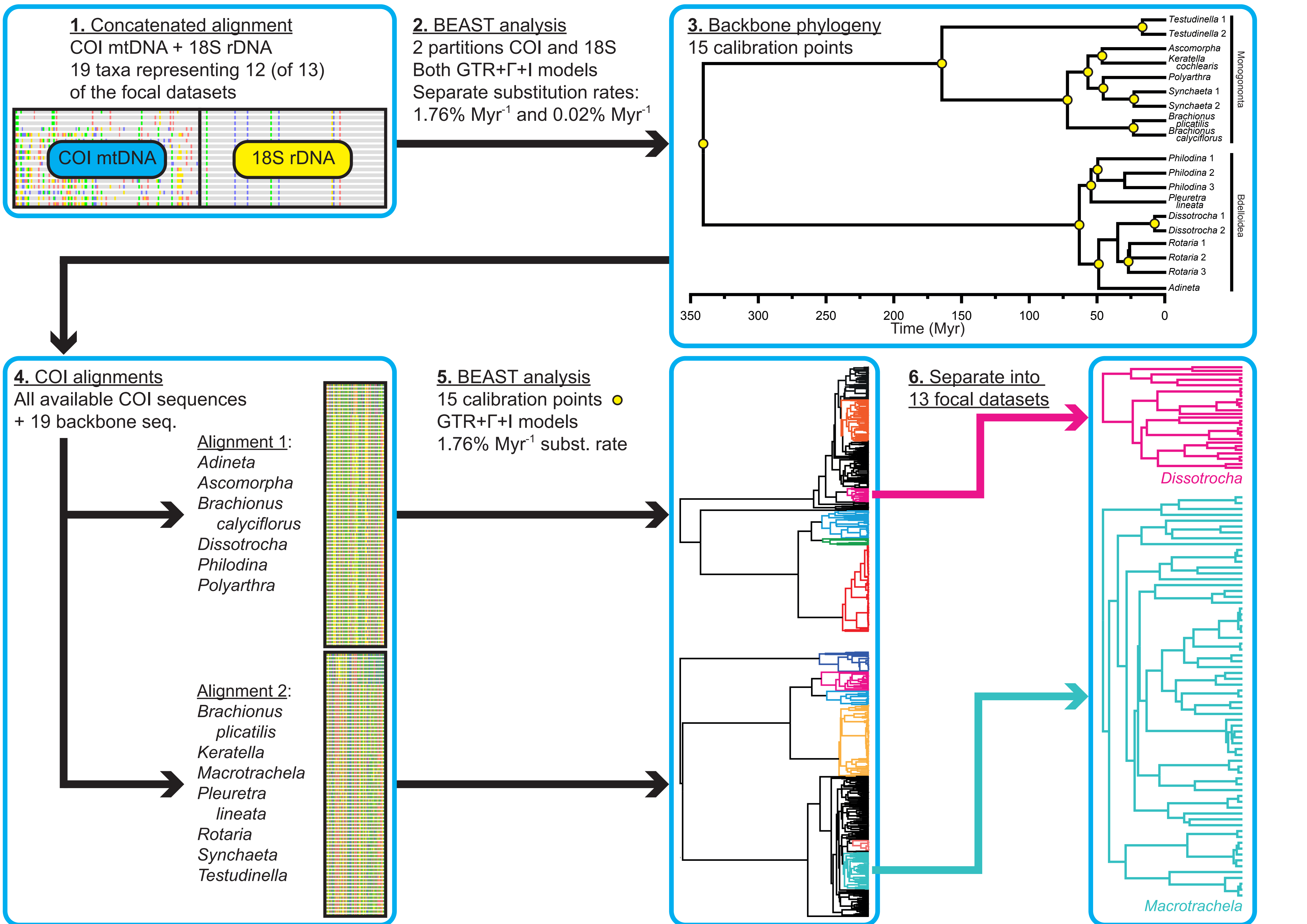

1. Concatenated alignment
COI mtDNA + 18S rDNA
19 taxa representing 12 (of 13)
of the focal datasets
COI mtDNA
18S rDNA
2. BEAST analysis
2 partitions COI and 18S
Both GTR+Γ+I models
Separate substitution rates:
1.76% Myr-1 and 0.02% Myr-1
3. Backbone phylogeny
15 calibration points
Testudinella 1
Testudinella 2
Ascomorpha
Keratella cochlearis
Polyarthra
Synchaeta 1
Synchaeta 2
Brachionus plicatilis
Brachionus calyciflorus
Philodina 1
Philodina 2
Philodina 3
Pleuretra lineata
Dissotrocha 1
Dissotrocha 2
Rotaria 1
Rotaria 2
Rotaria 3
Adineta
Monogononta
Bdelloidea
350
300
250
200
150
100
50
0
Time (Myr)
4. COI alignments
All available COI sequences
+ 19 backbone seq.
Alignment 1:
Adineta
Ascomorpha
Brachionus calyciflorus
Dissotrocha
Philodina
Polyarthra
Alignment 2:
Brachionus plicatilis
Keratella
Macrotrachela
Pleuretra lineata
Rotaria
Synchaeta
Testudinella
5. BEAST analysis
15 calibration points
GTR+Γ+I models
1.76% Myr-1 subst. rate
6. Separate into
13 focal datasets
Dissotrocha
Macrotrachela
