# Supplementary material for: SEXUAL SPECIES ARE SEPARATED BY LARGER GENETIC GAPS THAN ASEXUAL SPECIES IN ROTIFERS
Source: Evolution. 2014 Jul 25;68(10):2901–16. doi: 10.1111/evo.12483 (PMC4262011; doi:10.1111/evo.12483)
Supplement: Table S1 — . Specimen collection information and accessions numbers for the sequences generated for this study. Table S2. Summary information for each of the 13 datasets, including number of sequences, number of unique haplotypes, estimated diversity, constancy of diversification statistics, estimated ages, and accessions. Table S3. Intra- and interspecific diversity measures (genetic and phylogenetic distances) for each of the delimited GMYC entities. Table S4. Datasets were split up by sister clades as determined using the backbone phylogeny (Fig.2) and outgroup taxa were added to balance the sequence numbers for each alignment. Table S5. Output from GLMM analysis of GMYC model fit (P value) differences between bdelloid and monogonont rotifers and varying degrees of jackknifing. Figure S1. Phylogenetic methods workflow. Figure S2. Minimum interspecific genetic distance (raw pDistance) against minimum phylogenetic distance to the nearest neighbor (Myr). Figure S3. Species richness of each of the 13 rotifer datasets analyzed by GMYC but with different input ultrametric trees. Figure S4. Significance of the GMYC model fit when bdelloid (red) and monogonont (blue) trees are jackknifed by 20%, 25%, 33%, and 50%. File S1. Are the ultrametric trees robust to rate heterogeneity? File S2. How does phylogenetic reconstruction method affect species delimitation? File S3. Is sampling effort differentially affecting bdelloid and monogonont diversity estimates? [file evo0068-2901-SD1.zip › evo12483-sup-0005-SupMat.pdf]

Supplementary Materials

SPECIMEN COLLECTION, SEQUENCING AND CONCATENATION

Collection of **Ascomorpha, Keratella** *and* **Polyartha** *specimens*

Ascomorpha, *Keratella*, and *Polyarthra* populations were sampled between 2010 and 2012 from 42 water bodies using a 50μm mesh net. For each water body, specimens were isolated into a single sample and identified according to Koste (1978) and *Polyarthra* specific material from Shiel and Koste (1993). Full details of the sampling conditions (coordinates, depth, and date) and GenBank accession numbers are in Table S1.

COI sequencing of Ascomorpha, Keratella and Polyartha specimens

DNA from each single animal was extracted in 35μL of Chelex (InstaGene Matrix; BioRad). Cytochrome *c* oxidase subunit I (COI) was PCR amplified using Folmer primers (Folmer et al. 1994) LCOI (5′-GGT CAA CAA ATC ATA AAG ATA TTGG-3′) and HCOI (5′-TAA ACT TCA GGG TGA CCA AAAAAT CA-3′). Cycle conditions comprised initial denaturation at 94°C for 3 min, followed by 40 cycles of 94°C for 30 sec, 48°C for 1 min, and 72°C for 1 min, and a final extension step of 72°C for 7 min. PCR amplicons were purified using ExoSAP-IT (USB Corporation) and were sequenced using the same PCR primers and ABI BigDye 3.1 chemistry on an ABI 3730XL sequencer (Applied Biosystems) at the Edmund Mach Foundation (Research and Innovation Centre). All sequences were deposited in GenBank (accessions numbers KC618769 - KC619309). These sequences were assembled, checked and edited by eye using Geneious Pro v5.4.2 (Drummond et al. 2006).

18S sequencing

Amplification of 18S rDNA was performed using a nested PCR approach as outlined by Tang et al. (2012). The total fragment was amplified using 18SFnew (5’-AGA TTA AGC CAT

GCA TGT CT-3’) and 9R (5’-GAT CCT TCC GCA GGT TCA CCT AC-3’) using the following cycle conditions: initial denaturation at 95°C for 5 min, following by 38 cycles of 95°C for 1 min, 55°C for 1 min, and 72°C for 2 min, and a final extension at 72°C for 10 min. Three overlapping fragments of 18S were amplified using the previous amplicon as a template and the following primer pairs: 18SFnew (5’-AGA TTA AGC CAT GCA TGT CT-3’) – 4R (5’-GAA TTA CCG CGG CTG CTG G-3’), 18SCT1 (5’- TGG AGG GCA AGT CTG GTG CCA GC-3’) – 18Sbi (5’- GAG TCT CGT TCG TTA TCG GA-3’), and 18Sa2.0 (5’-ATG GTT GCA AAG CTG AAA C-3’) – 9R (5’-GAT CCT TCC GCA GGT TCA CCT AC-3’). Primers were either those of Giribet et al. (1996), or redesigned from five full length rotifer sequences (~1,800bp) obtained from GenBank (AF154566, AF154567, AY218118, AJ487049, DQ089733). These five sequences were aligned and priming sites were designed around regions that were conserved between bdelloid and monogonont sequences.

All PCR amplicons were purified and concentrated using Illustra GFX PCR DNA and Gel band purification kit as per the manufacturer’s protocol with a final volume ranging from 10µl to 20µl depending on the concentration of the amplicons. Purified products were sequenced using purified amplicons, 1:10 of the PCR primers and the ABI Big Dye Terminator v1.1 kit and run on an ABI 3770 automated sequencer. Sequences were checked and edited using Geneious Pro. Direct sequencing of 18S yielded only single copies with no evidence of divergent ribosomal copies. The orthology of 18S was further validated by searching the *Adineta vaga* genome (Flot et al. 2013) for 18S copies. BLAT (*Adineta* genome equivalent to BLAST) was used to search for the *Adineta* 18S sequence (KF561095), the resulting scaffolds (>500bp) were less than 1% divergent from each other. Furthermore, searching the genome for the other eighteen 18S sequences (used for the backbone phylogeny) returned the same set of scaffolds as for the *Adineta* (KF561095) search. These lines of evidence indicate that 18S has a single copy in *Adineta vaga* and that this particular copy is orthologous across the samples (bdelloid and monogonont) used here.

18S and COI concatenation

A backbone phylogeny was generated using a concatenated alignment COI and 18S sequences (Table S2). Sequences were chosen to represent as much of our focal data sets as possible from the available

resources (GenBank). For 18S, there was a limited collection of appropriately sized, annotated, and identified sequences available; therefore only 12 of the 13 data sets are represented and only 19 sequences in total were used (no 18S datum was available for *Macrotrachela*). The sequences used for the backbone phylogeny are shown in Table S2. Most of the COI and 18S sequences were concatenated from the same individuals (15 out of 19), but where this was not possible, sequences from the same morphospecies or genus were selected for concatenation. *Ascomorpha*, *Keratella*, and *Polyarthra* COI and 18S sequences belonged to specimens from the same morphospecies (Table S2), and for *Pleuretra*, a COI sequence from *P. lineata* (FJ426426) was concatenated with an 18S sequence from *P. hystrix* (JX494746). The COI and 18S sequences were aligned separately using MAFFT (Katoh et al. 2009) with the default settings and checked and edited in Geneious Pro. Non-alignable or substantially shorter sequences (<50% of the average alignment length) were discarded. These alignments had no indels or stop codons.

How are the two analyses split up?

The backbone phylogeny indicated that the following taxon pairs were sister clades: (*Ascomorpha*, *Keratella cochlearis*), (*Polyarthra*, *Synchaeta*), (*Brachionus calyciflorus*, *Brachionus plicatilis*), (*Philodina*, *Pleuretra lineata*), and (*Dissotrocha*, *Rotaria*). These lineages were split up into two alignments (Table S4) to maintain similar age distributions in each analysis. *Testudinella* and *Adineta* (which formed outgroups) and *Macrotrachela* (missing) were divided to maintain equivalent sequence numbers in each alignment.

The effect of incomplete sampling on constancy of net diversification rates

Incomplete sampling will result in γ becoming increasingly negative with more missing taxa because old nodes are disproportionately sampled over younger ones (Pybus and Harvey 2000). Incomplete sampling was corrected for both before and after the analyses. *A priori* correction was performed using a missing species simulator (by feeding in branches/splits, equivalent to species) based on a constant rate birth-death model (CorSiM - Cusimano et al. 2012). Missing species were simulated 1,000 times using the TreePar 2.5 package (Stadler 2011) in R, which requires estimates of net diversification rates under a constant birth-death model (estimated using ape) and a measure of

expected diversity (estimated using the Chao estimator - Chao 1984). The *post hoc* correction was performed using a Monte Carlo constant rates test (MCCR within laser - Pybus and Harvey 2000). The MCCR test is based on a null distribution of γ using pruned trees simulated 1,000 times under a constant rate with the same number of tips corresponding to the expected diversity (also estimated by Chao).

SCRIPT FOR MULTIBIRTHDEATH FUNCTION

```
library(ape)
#multibirthdeath function
multi.birthdeath<-function(phy){
if(!inherits(phy[[1]],"phylo"))
stop("object\"phy\"isnotofclass\"phylo\"")
dev<-function(a,r){
if(r<0||a>1)
return(1e+100)
lik<-0
N.sum<-0
for(iin(1:length(phy))){
N<-length(phy[[i]]$tip.label)
N.sum<-N.sum+N
x<-c(NA,branching.times(phy[[i]]))
lik<-lik-2*(lfactorial(N-1)+(N-2)*log(r)+r*sum(x[3:N])+N*log(1-a)-
2*sum(log(exp(r*x[2:N])-a)))}
N.sum<<-N.sum
return(lik)}
out<-nlm(function(p)dev(p[1],p[2]),c(0.1,0.2),hessian=TRUE)
```

```
if(out$estimate[1]<0){
out<-nlm(function(p)dev(0,p),0.2,hessian=TRUE)
para<-c(0,out$estimate)
inv.hessian<-try(solve(out$hessian))
se<-if(class(inv.hessian)=="try-error")
NA
elsesqrt(diag(inv.hessian))
se<-c(0,se)}
else{
para<-out$estimate
inv.hessian<-try(solve(out$hessian))
se<-if(class(inv.hessian)=="try-error")
c(NA,NA)
elsesqrt(diag(inv.hessian))}
Dev<-out$minimum
foo<-function(which,s){
i<-0.1
if(which==1){
p<-para[1]+s*i
bar<-function()dev(p,para[2])}
```

```
else{
p<-para[2]+s*i
bar<-function()dev(para[1],p)}
while(i>1e-09){
while(bar()<Dev+3.84)p<-p+s*i
p<-p-s*i
i<-i/10}
p}
CI<-mapply(foo,c(1,2,1,2),c(-1,-1,1,1))
dim(CI)<-c(2,2)
names(para)<-names(se)<-rownames(CI)<-c("d/b","b-d")
colnames(CI)<-c("lo","up")
obj<-list(tree=deparse(substitute(phy)),N=N.sum,dev=Dev,
para=para,se=se,CI=CI)
class(obj)<-"birthdeath"
obj}
#Bdelloid, Monogonont and Rotifera trees were concatenated into a
single multiphylo object
bdelloid.trees<-
c(adin.prune,diss.prune,macr.prune,phil.prune,pleu.prune,rota.prune)
```

```
monogonont.trees<-
c(asco.prune,brac.prune,brap.prune,kera.prune,poly.prune,sync.prune,
test.prune)
all.trees<-c(bdelloid.trees,monogonont.trees)
#run code
multi.birthdeath(bdelloid.trees)
multi.birthdeath(monogonont.trees)
multi.birthdeath(all.trees)
#A likelihood ratio test was used to compare the fit of a nested
#model (bdelloids or monogononts) to the global model (Rotifera),
#where X is the log likelihood of the nested model and Y is the log
#likelihood of global model
1-pchisq(2*(X-(Y)),2)
```

SUPPLEMENTARY FILE S1

Are the ultrametric trees robust to rate heterogeneity?

Cladogenesis rates may be affected by differences in rate heterogeneity. Rate heterogeneity within a gene tree will result in branch length variation and potentially the artificial stretching of branches. Genetic distances among sequences should correlate strongly with branch lengths on a gene tree, but this relationship may be imperfect when trees are made ultrametric. Rate variation in one part of the dataset may lead to over-smoothing and stretching of other less diverse parts of the final gene tree; this stretching leads to biases in the GMYC analysis, which is concerned primarily with branching rates. Moreover, different clades may have different levels of rate heterogeneity, and strong departures from uniformity across the tree could result in artificially stretched branch lengths (i.e. artificially old tips). These artificially stretched tips may result in poor GMYC ESU estimation. We assessed the effect of rate heterogeneity on artificial branch stretching by comparing the raw minimum genetic distance and the minimum phylogenetic distance (on an ultrametric tree) between GMYC entities. A positive linear correlation between the two measures would indicate a clock-like rate, while a lack of correlation would indicate a departure from a molecular clock or artefacts from the method of phylogenetic reconstruction. This diagnostic was used iteratively to develop the phylogenetic analyses described above.

The main difference in discreteness between bdelloids and monogononts was not an artefact of the effect of smoothing on the greater substitution rate heterogeneity observed in bdelloids than monogononts, as the correlation between raw genetic and phylogenetic distance was equivalent between the two clades (LM: $F_{1,425}=273.3$, $P<0.001$; Fig. S2).

SUPPLEMENTARY FILE S2

How does phylogenetic reconstruction method affect species delimitation?

To assess the robustness of the trees reconstructed by two large combined analyses in terms of topology and rate smoothing, the number of GMYC clusters delimited from these trees was compared to ones reconstructed independently from the other data sets (i.e. 13 separate analyses instead of two) using both maximum likelihood (ML) and Bayesian inference (BI) techniques. ML trees were reconstructed using RAxML webservers (Stamatakis et al. 2008) with a Gamma model of rate heterogeneity, maximum likelihood model search, and an estimated proportion of invariable sites. The r8s software (Sanderson 2003) was used to perform nonparametric rate smoothing (NPRS). This method smooths rate changes among lineages while penalising fast rate changes from mother to daughter. The level of smoothing was optimised using a cross validation procedure.

Two separate BEAST runs, strict and relaxed clock models, were performed for each clade. BEAST input files were generated using BEAUti v1.7.5, each search ran with a substitution rate of 1.76% per million years (the most accepted rate for most invertebrates - Wilke et al. 2009) under either an uncorrelated lognormal relaxed molecular clock or a strict molecular clock and a birth-death (Gernhard 2008) tree prior. Each data set was run for at least 30,000,000 generations sampling every 1,000 steps on the CIPRES Science Gateway server (Miller et al. 2010). All of the other options were kept as the BEAUti default settings. The effective sample size (ESS) of each run was determined using Tracer v1.5 (Rambaut and Drummond 2007) and only trees with an ESS of at least 200 were kept (as recommended in the BEAST documentation). TreeAnnotator v1.6.1 was used to summarise the trees with a 10% burnin to give a 50% majority-rule consensus ‘maximum clade credibility tree’ with target node heights.

The number of evolutionary significant units (GMYC species) was obtained from each tree using the GMYC (described in the main text) and compared to those produced using the larger analyses from the combined alignments (described in the main text).

There was no relationship between the method of phylogenetic tree inference (ML/BI) and the number of GMYC species delimited (t_{12}=-0.17, *P*=0.87; Fig. S3) and no significant difference between the three Bayesian methods (i.e. [1] analysed together in large matrices, [2] individually with a lognormal relaxed clock, and [3] with a strict clock; $F_{2,36}$=0.022, *P*=0.98; Fig. S2), therefore only the results from the larger primary analyses will be shown.

SUPPLEMENTARY FILE S3

Is sampling effort differentially affecting bdelloid and monogonont diversity estimates?

The transition from Yule to Coalescent branching rates in the tree is used by the GMYC to detect species. Increased sampling can either supplement existing haplotypes or fill in gaps present in the sample; supplementation will increase the support for the coalescent part of the tree, while filling in the gaps will increase the representation of the Yule section. Higher support for the GMYC is indicative of a strong transition between the processes, this happens when the species richness in the sample is saturated (Yule process) and additional samples supplement the populations (Coalescent process). Adding new samples to a poorly sampled tree, with a low representation of the species richness, will tend to find more novelty and so will reduce the significance of a branching rate transition by supporting the Yule side of the tree. Supplementing pre-existing haplotypes is more likely than discovering novel haplotypes, and so increased sampling will tend to improve the support of GMYC units, but the slope of this improvement (associated with sampling effort) will indicate how well sampled the diversity in the data set is.

The effect of sampling effort on how the GMYC model fits to the data and how this differs between bdelloid and monogonont rotifers was tested by jack-knifing gene trees and delimiting species from them using the GMYC model. Each tree was jack-knifed by a fifth, a quarter, a third and a half 100 times, and for each of these trees the GMYC model with a single threshold was performed. In total 5,200 GMYC analyses were performed (400 replicates for each clade). A generalised linear mixed effects model with a penalised quasi-likelihood and a quasibinomial error structure was used with GMYC model significance (using the P value of the likelihood ratio test) as the explanatory variable and group (bdelloid vs. monogonont) and proportion of pruned tips (20%, 25%, 33%, and 50%) as the response variables. Data set was blocked out as a random variable.

The average GMYC model significance was qualitatively higher for 100% sampled (i.e. unpruned) monogonont data sets compared to bdelloid data sets (GMYC P values: 0.0081 vs. 0.014; Table S2), and this remained true for each of the jack-knifed proportions (Fig. S4). Jack-knife analyses indicate

that the GMYC (*P* values) is more stable in monogonont samples than bdelloids. The slope of the decrease in the GMYC model fit with increasing levels of jack-knifing is steeper in bdelloids than it is for monogononts (Table S5; Fig. S4), which indicates that diversity in these monogonont samples is closer to saturation than in the bdelloids.

Sampling will be related to GMYC support, but the slope at which support improves with increased sampling will reflect the likelihood that increased sampling supplements existing haplotypes (Coalescent) as opposed to being novel (Yule). The slope at which monogonont GMYC support increases is shallower than that of the bdelloids, this indicates that additional samples are more likely to supplement pre-existing haplotypes, thereby supporting the Coalescent side of the tree. This indicates that monogononts diversity in these data sets it more representative than the bdelloids, and that including additional sequences tends to supplement closely related genotypes to species clusters, while maintaining, rather than filling, interspecific gaps (as would be expected of the bdelloid data sets).

REFERENCES

Chao, A. 1984. Nonparametric estimation of the number of classes in a population. Scand. J. Stat. 11:265–270.

Cusimano, N., T. Stadler, and S. S. Renner. 2012. A new method for handling missing species in diversification analysis applicable to randomly or nonrandomly sampled phylogenies. Syst. Biol. 61:785–92.

Drummond, A. J., M. Kearse, J. Heled, R. Moir, T. Thierer, B. Ashton, A. Wilson, and S. Stones-Havas. 2006. Geneious v5.4.2.

Flot, J.-F., B. Hespeels, X. Li, B. Noel, I. R. Arkhipova, E. G. J. Danchin, A. Hejnol, B. Henrissat, R. Koszul, J.-M. Aury, V. Barbe, R.-M. Barthélémy, J. Bast, G. a Bazykin, O. Chabrol, A. Couloux, M. Da Rocha, C. Da Silva, E. A. Gladyshev, P. Gouret, O. Hallatschek, B. Hecox-Lea, K. Labadie, B. Lejeune, O. Piskurek, J. Poulain, F. Rodriguez, J. F. Ryan, O. a Vakhrusheva, E. Wajnberg, B. Wirth, I. Yushenova, M. Kellis, A. S. Kondrashov, D. B. Mark Welch, P. Pontarotti, J. Weissenbach, P. Wincker, O. Jaillon, and K. Van Doninck. 2013. Genomic evidence for ameiotic evolution in the bdelloid rotifer *Adineta vaga*. Nature 500:453–457.

Folmer, O., M. Black, W. Hoeh, R. Lutz, and R. Vrijenhoek. 1994. DNA primers for amplification of mitochondrial cytochrome *c* oxidase subunit I from diverse metazoan invertebrates. Mol. Mar. Biol. Biotechnol. 3:294–9.

Gernhard, T. 2008. The conditioned reconstructed process. J. Theor. Biol. 253:769–78.

Giribet, G., S. Carracnza, J. Baguñà, M. Riutort, and C. Ribera. 1996. First Molecular Evidence Arthropoda Clade for the Existence of a Tardigrada. Mol. Biol. Evol. 13:76–84.

Koste, W. 1978. Rotatoria. Die Rädertiere Mitteleuropas. II Tafelband. Gebrüder Borntraeger, Berlin.

Miller, M. A., W. Pfeiffer, and T. Schwartz. 2010. Creating the CIPRES Science Gateway for inference of large phylogenetic trees. Proc. Gatew. Comput. Environ. Work. 1–8. Ieee.

Pybus, O. G., and P. H. Harvey. 2000. Testing macro-evolutionary models using incomplete molecular phylogenies. Proc. R. Soc. London B 267:2267–72.

Rambaut, A., and A. J. Drummond. 2007. Tracer v1.4.

Sanderson, M. J. 2003. r8s: Inferring absolute rates of molecular evolution and divergence times in the absence of a molecular clock. Bioinformatics 19:301–302.

Shiel, R. J., and W. Koste. 1993. Rotifera from Australian inland waters. IX. Gastropodidae, Synchaetidae, Asplanchnidae (Rotifera: Monogononta). Trans. R. Soc. South Aust. 113:111–139.

Stadler, T. 2011. TreePar in R - Estimating diversification rates in phylogenies.

Tang, C. Q., F. Leasi, U. Obertegger, A. Kieneke, T. G. Barraclough, and D. Fontaneto. 2012. The widely used small subunit 18S rDNA molecule greatly underestimates true diversity in biodiversity surveys of the meiofauna. Proc. Natl. Acad. Sci. U. S. A. 109:16208–16212.

Wilke, T., R. Schultheiß, and C. Albrecht. 2009. As time goes by : A simple fool's guide to molecular clock approaches in invertebrates. Am. Malacol. Bull. 27:25–45.
