# Supplementary material for: SEXUAL SPECIES ARE SEPARATED BY LARGER GENETIC GAPS THAN ASEXUAL SPECIES IN ROTIFERS
Source: Evolution. 2014 Jul 25;68(10):2901–16. doi: 10.1111/evo.12483 (PMC4262011; doi:10.1111/evo.12483)
Supplement: Table S1 — . Specimen collection information and accessions numbers for the sequences generated for this study. Table S2. Summary information for each of the 13 datasets, including number of sequences, number of unique haplotypes, estimated diversity, constancy of diversification statistics, estimated ages, and accessions. Table S3. Intra- and interspecific diversity measures (genetic and phylogenetic distances) for each of the delimited GMYC entities. Table S4. Datasets were split up by sister clades as determined using the backbone phylogeny (Fig.2) and outgroup taxa were added to balance the sequence numbers for each alignment. Table S5. Output from GLMM analysis of GMYC model fit (P value) differences between bdelloid and monogonont rotifers and varying degrees of jackknifing. Figure S1. Phylogenetic methods workflow. Figure S2. Minimum interspecific genetic distance (raw pDistance) against minimum phylogenetic distance to the nearest neighbor (Myr). Figure S3. Species richness of each of the 13 rotifer datasets analyzed by GMYC but with different input ultrametric trees. Figure S4. Significance of the GMYC model fit when bdelloid (red) and monogonont (blue) trees are jackknifed by 20%, 25%, 33%, and 50%. File S1. Are the ultrametric trees robust to rate heterogeneity? File S2. How does phylogenetic reconstruction method affect species delimitation? File S3. Is sampling effort differentially affecting bdelloid and monogonont diversity estimates? [file evo0068-2901-SD1.zip › evo12483-sup-0006-tableS1.pdf]

Table S1. Specimen collection information and accessions numbers for the sequences generated for this study.

ID	Genus	Species	Lake	Sampling Date	Depth	Latitude	Longitude	Net Mesh	GenBank
4.9	*Ascomorpha*	*ecaudis*	Roncone	30/11/2010	water column	45.985407820	10.674848557	50µm	KC618777
71.1	*Ascomorpha*	*ecaudis*	Raiermoos	25/03/2012	water column	46.746227574	11.664562225	50µm	KC618769
71.2	*Ascomorpha*	*ecaudis*	Raiermoos	25/03/2012	water column	46.746227574	11.664562225	50µm	KC618770
71.3	*Ascomorpha*	*ecaudis*	Raiermoos	25/03/2012	water column	46.746227574	11.664562225	50µm	KC618771
71.4	*Ascomorpha*	*ecaudis*	Raiermoos	25/03/2012	water column	46.746227574	11.664562225	50µm	KC618772
71.5	*Ascomorpha*	*ecaudis*	Raiermoos	25/03/2012	water column	46.746227574	11.664562225	50µm	KC618773
71.6	*Ascomorpha*	*ecaudis*	Raiermoos	25/03/2012	water column	46.746227574	11.664562225	50µm	KC618774
71.7	*Ascomorpha*	*ecaudis*	Raiermoos	25/03/2012	water column	46.746227574	11.664562225	50µm	KC618775
71.8	*Ascomorpha*	*ecaudis*	Raiermoos	25/03/2012	water column	46.746227574	11.664562225	50µm	KC618776
113.2	*Ascomorpha*	*ecaudis*	TovelA15m	05/06/2012	15m	46.261402873	10.951116085	50µm	KC618778
113.4	*Ascomorpha*	*ecaudis*	TovelA15m	05/06/2012	15m	46.261402873	10.951116085	50µm	KC618779
113.5	*Ascomorpha*	*ecaudis*	TovelA15m	05/06/2012	15m	46.261402873	10.951116085	50µm	KC618780
113.6	*Ascomorpha*	*ecaudis*	TovelA15m	05/06/2012	15m	46.261402873	10.951116085	50µm	KC618781
127.1	*Ascomorpha*	*ecaudis*	ColBricon	28/06/2012	water column	46.281626903	11.765413284	50µm	KC618782
127.3	*Ascomorpha*	*ecaudis*	ColBricon	28/06/2012	water column	46.281626903	11.765413284	50µm	KC618783
127.4	*Ascomorpha*	*ecaudis*	ColBricon	28/06/2012	water column	46.281626903	11.765413284	50µm	KC618784
127.5	*Ascomorpha*	*ecaudis*	ColBricon	28/06/2012	water column	46.281626903	11.765413284	50µm	KC618785
127.6	*Ascomorpha*	*ecaudis*	ColBricon	28/06/2012	water column	46.281626903	11.765413284	50µm	KC618786
135.1	*Ascomorpha*	*ecaudis*	TovelA15m	04/07/2012	15m	46.261402873	10.951116085	50µm	KC618787
176.1	*Ascomorpha*	*ecaudis*	Terlago	08/08/2012	water column	46.126110152	11.058747768	50µm	KC618788
176.4	*Ascomorpha*	*ecaudis*	Terlago	08/08/2012	water column	46.126110152	11.058747768	50µm	KC618789
182.1	*Ascomorpha*	*ovalis*	Tenno	20/08/2012	water column	45.939690782	10.812950134	50µm	KC618790
182.2	*Ascomorpha*	*ovalis*	Tenno	20/08/2012	water column	45.939690782	10.812950134	50µm	KC618791
182.3	*Ascomorpha*	*ovalis*	Tenno	20/08/2012	water column	45.939690782	10.812950134	50µm	KC618792
182.4	*Ascomorpha*	*ovalis*	Tenno	20/08/2012	water column	45.939690782	10.812950134	50µm	KC618793
182.5	*Ascomorpha*	*ovalis*	Tenno	20/08/2012	water column	45.939690782	10.812950134	50µm	KC618794
182.6	*Ascomorpha*	*ovalis*	Tenno	20/08/2012	water column	45.939690782	10.812950134	50µm	KC618795
182.7	*Ascomorpha*	*ovalis*	Tenno	20/08/2012	water column	45.939690782	10.812950134	50µm	KC618796
182.8	*Ascomorpha*	*ovalis*	Tenno	20/08/2012	water column	45.939690782	10.812950134	50µm	KC618797
185.1	*Ascomorpha*	*ovalis*	Lases	20/08/2012	water column	46.141782738	11.219186783	50µm	KC618798
185.2	*Ascomorpha*	*ovalis*	Lases	20/08/2012	water column	46.141782738	11.219186783	50µm	KC618799
185.3	*Ascomorpha*	*ovalis*	Lases	20/08/2012	water column	46.141782738	11.219186783	50µm	KC618800
185.4	*Ascomorpha*	*ovalis*	Lases	20/08/2012	water column	46.141782738	11.219186783	50µm	KC618801
185.5	*Ascomorpha*	*ovalis*	Lases	20/08/2012	water column	46.141782738	11.219186783	50µm	KC618802
185.6	*Ascomorpha*	*ovalis*	Lases	20/08/2012	water column	46.141782738	11.219186783	50µm	KC618803
185.7	*Ascomorpha*	*ovalis*	Lases	20/08/2012	water column	46.141782738	11.219186783	50µm	KC618804
185.8	*Ascomorpha*	*ovalis*	Lases	20/08/2012	water column	46.141782738	11.219186783	50µm	KC618805
218.4	*Ascomorpha*	*ecaudis*	TovelA15m	02/10/2012	15m	46.261402873	10.951116085	50µm	KC618806
3.1	*Keratella*	*cochlearis*	Terlago	30/11/2010	water column	46.126110152	11.058747768	50µm	KC618807
3.2	*Keratella*	*cochlearis*	Terlago	30/11/2010	water column	46.126110152	11.058747768	50µm	KC618808
3.4	*Keratella*	*cochlearis*	Terlago	30/11/2010	water column	46.126110152	11.058747768	50µm	KC618809
3.5	*Keratella*	*cochlearis*	Terlago	30/11/2010	water column	46.126110152	11.058747768	50µm	KC618810
3.6	*Keratella*	*cochlearis*	Terlago	30/11/2010	water column	46.126110152	11.058747768	50µm	KC618811
7.1	*Keratella*	*cochlearis*	Levico	20/12/2010	water column	46.013072	11.286349	50µm	KC618812
7.2	*Keratella*	*cochlearis*	Levico	20/12/2010	water column	46.013072	11.286349	50µm	KC618813
7.3	*Keratella*	*cochlearis*	Levico	20/12/2010	water column	46.013072	11.286349	50µm	KC618814
7.5	*Keratella*	*cochlearis*	Levico	20/12/2010	water column	46.013072	11.286349	50µm	KC618815
7.6	*Keratella*	*cochlearis*	Levico	20/12/2010	water column	46.013072	11.286349	50µm	KC618816
7.7	*Keratella*	*cochlearis*	Levico	20/12/2010	water column	46.013072	11.286349	50µm	KC618817
7.8	*Keratella*	*cochlearis*	Levico	20/12/2010	water column	46.013072	11.286349	50µm	KC618818
5.5	*Keratella*	*cochlearis*	Lavarone	30/11/2010	water column	45.937333058	11.253111362	50µm	KC618819
5.6	*Keratella*	*cochlearis*	Lavarone	30/11/2010	water column	45.937333058	11.253111362	50µm	KC618820
5.8	*Keratella*	*cochlearis*	Lavarone	30/11/2010	water column	45.937333058	11.253111362	50µm	KC618821
9.3	*Keratella*	*cochlearis*	Raiermoos	20/06/2010	water column	46.746271685	11.664648056	50µm	KC618822
9.4	*Keratella*	*cochlearis*	Raiermoos	20/06/2010	water column	46.746271685	11.664648056	50µm	KC618823
9.5	*Keratella*	*cochlearis*	Raiermoos	20/06/2010	water column	46.746271685	11.664648056	50µm	KC618824
9.6	*Keratella*	*cochlearis*	Raiermoos	20/06/2010	water column	46.746271685	11.664648056	50µm	KC618825
14.1	*Keratella*	*cochlearis*	Crespeina	29/08/2010	water column	46.568272851	11.825087070	50µm	KC618826
14.2	*Keratella*	*cochlearis*	Crespeina	29/08/2010	water column	46.568272851	11.825087070	50µm	KC618827
14.3	*Keratella*	*cochlearis*	Crespeina	29/08/2010	water column	46.568272851	11.825087070	50µm	KC618828
14.4	*Keratella*	*cochlearis*	Crespeina	29/08/2010	water column	46.568272851	11.825087070	50µm	KC618830
14.5	*Keratella*	*cochlearis*	Crespeina	29/08/2010	water column	46.568272851	11.825087070	50µm	KC618829
14.6	*Keratella*	*cochlearis*	Crespeina	29/08/2010	water column	46.568272851	11.825087070	50µm	KC618831
14.7	*Keratella*	*cochlearis*	Crespeina	29/08/2010	water column	46.568272851	11.825087070	50µm	KC618832
14.8	*Keratella*	*cochlearis*	Crespeina	29/08/2010	water column	46.568272851	11.825087070	50µm	KC618833
15.1	*Keratella*	*cochlearis*	Antholz	10/08/2010	water column	46.883045666	12.164440155	50µm	KC618834
15.4	*Keratella*	*cochlearis*	Antholz	10/08/2010	water column	46.883045666	12.164440155	50µm	KC618835
16.18	*Keratella*	*cochlearis*	Levico	18/01/2011	water column	46.013072	11.286349	50µm	KC618836
16.23	*Keratella*	*cochlearis*	Levico	18/01/2011	water column	46.013072	11.286349	50µm	KC618837
18.1	*Keratella*	*cochlearis*	Crespeina	29/08/2010	water column	46.568272851	11.825087070	50µm	KC618838
18.2	*Keratella*	*cochlearis*	Crespeina	29/08/2010	water column	46.568272851	11.825087070	50µm	KC618839
18.3	*Keratella*	*cochlearis*	Crespeina	29/08/2010	water column	46.568272851	11.825087070	50µm	KC618840
18.4	*Keratella*	*cochlearis*	Crespeina	29/08/2010	water column	46.568272851	11.825087070	50µm	KC618841
18.5	*Keratella*	*cochlearis*	Crespeina	29/08/2010	water column	46.568272851	11.825087070	50µm	KC618842
18.6	*Keratella*	*cochlearis*	Crespeina	29/08/2010	water column	46.568272851	11.825087070	50µm	KC618843
18.7	*Keratella*	*cochlearis*	Crespeina	29/08/2010	water column	46.568272851	11.825087070	50µm	KC618844
18.8	*Keratella*	*cochlearis*	Crespeina	29/08/2010	water column	46.568272851	11.825087070	50µm	KC618845
54.1	*Keratella*	*cochlearis*	Serraia	22/09/2011	water column	46.136370698	11.251115799	50µm	KC618846
54.2	*Keratella*	*cochlearis*	Serraia	22/09/2011	water column	46.136370698	11.251115799	50µm	KC618847
54.3	*Keratella*	*cochlearis*	Serraia	22/09/2011	water column	46.136370698	11.251115799	50µm	KC618848
54.4	*Keratella*	*cochlearis*	Serraia	22/09/2011	water column	46.136370698	11.251115799	50µm	KC618849
55.2	*Keratella*	*cochlearis*	VoelserWeiher	26/09/2011	water column	46.524163	11.523762	50µm	KC618852
55.3	*Keratella*	*cochlearis*	VoelserWeiher	26/09/2011	water column	46.524163	11.523762	50µm	KC618853
55.4	*Keratella*	*cochlearis*	VoelserWeiher	26/09/2011	water column	46.524163	11.523762	50µm	KC618854
55.5	*Keratella*	*cochlearis*	VoelserWeiher	26/09/2011	water column	46.524163	11.523762	50µm	KC618855

55.7	*Keratella*	*cochlearis*	VoelserWeiher	26/09/2011	water column	46.524163	11.523762	50µm	KC618856
55.10	*Keratella*	*cochlearis*	VoelserWeiher	26/09/2011	water column	46.524163	11.523762	50µm	KC618850
55.12	*Keratella*	*cochlearis*	VoelserWeiher	26/09/2011	water column	46.524163	11.523762	50µm	KC618851
138.1	*Keratella*	*cochlearis*	TovelA15m	04/07/2012	15m	46.261402873	10.951116085	50µm	KC618857
138.4	*Keratella*	*cochlearis*	TovelA15m	04/07/2012	15m	46.261402873	10.951116085	50µm	KC618858
138.6	*Keratella*	*cochlearis*	TovelA15m	04/07/2012	15m	46.261402873	10.951116085	50µm	KC618859
138.7	*Keratella*	*cochlearis*	TovelA15m	04/07/2012	15m	46.261402873	10.951116085	50µm	KC618860
138.8	*Keratella*	*cochlearis*	TovelA15m	04/07/2012	15m	46.261402873	10.951116085	50µm	KC618861
115.2	*Keratella*	*cochlearis*	TovelA15m	05/06/2012	15m	46.261402873	10.951116085	50µm	KC618862
115.3	*Keratella*	*cochlearis*	TovelA15m	05/06/2012	15m	46.261402873	10.951116085	50µm	KC618863
115.6	*Keratella*	*cochlearis*	TovelA15m	05/06/2012	15m	46.261402873	10.951116085	50µm	KC618864
115.7	*Keratella*	*cochlearis*	TovelA15m	05/06/2012	15m	46.261402873	10.951116085	50µm	KC618865
102PD1TovelA4	*Polyarthra*	*dolichoptera*	Tovel	02/05/2012	5m	46.261402873	10.951116085	50µm	KC618984
102PD2TovelA4	*Polyarthra*	*dolichoptera*	Tovel	02/05/2012	5m	46.261402873	10.951116085	50µm	KC618982
102PD3TovelA4	*Polyarthra*	*dolichoptera*	Tovel	02/05/2012	5m	46.261402873	10.951116085	50µm	KC618983
102PD4TovelA4	*Polyarthra*	*dolichoptera*	Tovel	02/05/2012	5m	46.261402873	10.951116085	50µm	KC618945
102PD5TovelA4	*Polyarthra*	*dolichoptera*	Tovel	02/05/2012	5m	46.261402873	10.951116085	50µm	KC618946
102PD6TovelA4	*Polyarthra*	*dolichoptera*	Tovel	02/05/2012	5m	46.261402873	10.951116085	50µm	KC618950
102PD7TovelA4	*Polyarthra*	*dolichoptera*	Tovel	02/05/2012	5m	46.261402873	10.951116085	50µm	KC618949
102PD8TovelA4	*Polyarthra*	*dolichoptera*	Tovel	02/05/2012	5m	46.261402873	10.951116085	50µm	KC618985
104PD1TovelA15	*Polyarthra*	*dolichoptera*	Tovel	02/05/2012	15m	46.261402873	10.951116085	50µm	KC618987
104PD3TovelA15	*Polyarthra*	*dolichoptera*	Tovel	02/05/2012	15m	46.261402873	10.951116085	50µm	KC618981
104PD4TovelA15	*Polyarthra*	*dolichoptera*	Tovel	02/05/2012	15m	46.261402873	10.951116085	50µm	KC618972
104PD5TovelA15	*Polyarthra*	*dolichoptera*	Tovel	02/05/2012	15m	46.261402873	10.951116085	50µm	KC618973
104PD6TovelA15	*Polyarthra*	*dolichoptera*	Tovel	02/05/2012	15m	46.261402873	10.951116085	50µm	KC618895
104PD7TovelA15	*Polyarthra*	*dolichoptera*	Tovel	02/05/2012	15m	46.261402873	10.951116085	50µm	KC618947
104PD8TovelA15	*Polyarthra*	*dolichoptera*	Tovel	02/05/2012	15m	46.261402873	10.951116085	50µm	KC618986
111PD2Tovela4m	*Polyarthra*	*dolichoptera*	Tovel	05/06/2012	5m	46.261402873	10.951116085	50µm	KC618989
111PD3Tovela4m	*Polyarthra*	*dolichoptera*	Tovel	05/06/2012	5m	46.261402873	10.951116085	50µm	KC618991
111PD4Tovela4m	*Polyarthra*	*dolichoptera*	Tovel	05/06/2012	5m	46.261402873	10.951116085	50µm	KC618977
111PD6Tovela4m	*Polyarthra*	*dolichoptera*	Tovel	05/06/2012	5m	46.261402873	10.951116085	50µm	KC618979
111PD7Tovela4m	*Polyarthra*	*dolichoptera*	Tovel	05/06/2012	5m	46.261402873	10.951116085	50µm	KC618993
112PD1Tovela15m	*Polyarthra*	*dolichoptera*	Tovel	05/06/2012	15m	46.261402873	10.951116085	50µm	KC618980
112PD2Tovela15	*Polyarthra*	*dolichoptera*	Tovel	05/06/2012	15m	46.261402873	10.951116085	50µm	KC618975
112PD3Tovela15m	*Polyarthra*	*dolichoptera*	Tovel	05/06/2012	15m	46.261402873	10.951116085	50µm	KC618990
112PD4Tovela15m	*Polyarthra*	*dolichoptera*	Tovel	05/06/2012	15m	46.261402873	10.951116085	50µm	KC618992
112PD5Tovela15m	*Polyarthra*	*dolichoptera*	Tovel	05/06/2012	15m	46.261402873	10.951116085	50µm	KC618995
112PD6Tovela15m	*Polyarthra*	*dolichoptera*	Tovel	05/06/2012	15m	46.261402873	10.951116085	50µm	KC618994
112PD7Tovela15m	*Polyarthra*	*dolichoptera*	Tovel	05/06/2012	15m	46.261402873	10.951116085	50µm	KC618978
121PD2Passen	*Polyarthra*	*dolichoptera*	Passensee	27/06/2012	water column	46.888882456	11.769962311	50µm	KC619036
121PD3Passen	*Polyarthra*	*dolichoptera*	Passensee	27/06/2012	water column	46.888882456	11.769962311	50µm	KC619031
121PD4Passen	*Polyarthra*	*dolichoptera*	Passensee	27/06/2012	water column	46.888882456	11.769962311	50µm	KC619037
121PD5Passen	*Polyarthra*	*dolichoptera*	Passensee	27/06/2012	water column	46.888882456	11.769962311	50µm	KC619038
121PD6Passen	*Polyarthra*	*dolichoptera*	Passensee	27/06/2012	water column	46.888882456	11.769962311	50µm	KC619032
121PD7Passen	*Polyarthra*	*dolichoptera*	Passensee	27/06/2012	water column	46.888882456	11.769962311	50µm	KC619039
121PD8Passen	*Polyarthra*	*dolichoptera*	Passensee	27/06/2012	water column	46.888882456	11.769962311	50µm	KC619033
123PD3WilderS	*Polyarthra*	*dolichoptera*	WilderSee	10/10/2010	water column	46.898208270	11.588859500	50µm	KC619040
123PD4WilderS	*Polyarthra*	*dolichoptera*	WilderSee	10/10/2010	water column	46.898208270	11.588859500	50µm	KC619044
123PD8WilderS	*Polyarthra*	*dolichoptera*	WilderSee	10/10/2010	water column	46.898208270	11.588859500	50µm	KC619035
124PD3Timmels	*Polyarthra*	*dolichoptera*	TimmelsSchwarzsee	22/08/2010	water column	46.928286160	11.164855957	50µm	KC619041
124PD5Timmels	*Polyarthra*	*dolichoptera*	TimmelsSchwarzsee	22/08/2010	water column	46.928286160	11.164855957	50µm	KC619034
124PD6Timmels	*Polyarthra*	*dolichoptera*	TimmelsSchwarzsee	22/08/2010	water column	46.928286160	11.164855957	50µm	KC619043
124PD8Timmels	*Polyarthra*	*dolichoptera*	TimmelsSchwarzsee	22/08/2010	water column	46.928286160	11.164855957	50µm	KC619042
136PD6Tovel15m	*Polyarthra*	*dolichoptera*	Tovel	04/07/2012	water column	46.261402873	10.951116085	50µm	KC619045
136PD7Tovel15m	*Polyarthra*	*dolichoptera*	Tovel	04/07/2012	water column	46.261402873	10.951116085	50µm	KC619046
136PD8Tovel15m	*Polyarthra*	*dolichoptera*	Tovel	04/07/2012	water column	46.261402873	10.951116085	50µm	KC619047
137PD2TovelB	*Polyarthra*	*dolichoptera*	Tovel	04/07/2012	water column	46.257211776	10.944786072	50µm	KC619048
137PD3TovelB	*Polyarthra*	*dolichoptera*	Tovel	04/07/2012	water column	46.257211776	10.944786072	50µm	KC619049
137PD4TovelB	*Polyarthra*	*dolichoptera*	Tovel	04/07/2012	water column	46.257211776	10.944786072	50µm	KC619050
137PD5TovelB	*Polyarthra*	*dolichoptera*	Tovel	04/07/2012	water column	46.257211776	10.944786072	50µm	KC619051
137PD6TovelB	*Polyarthra*	*dolichoptera*	Tovel	04/07/2012	water column	46.257211776	10.944786072	50µm	KC619052
137PD8TovelB	*Polyarthra*	*dolichoptera*	Tovel	04/07/2012	water column	46.257211776	10.944786072	50µm	KC619053
139PD1Prags	*Polyarthra*	*dolichoptera*	PragserWildsee	08/10/2010	water column	46.698023000	12.086592000	50µm	KC619054
139PD2Prags	*Polyarthra*	*dolichoptera*	PragserWildsee	08/10/2010	water column	46.698023000	12.086592000	50µm	KC619055
139PD3Prags	*Polyarthra*	*dolichoptera*	PragserWildsee	08/10/2010	water column	46.698023000	12.086592000	50µm	KC619056
139PD4Prags	*Polyarthra*	*dolichoptera*	PragserWildsee	08/10/2010	water column	46.698023000	12.086592000	50µm	KC619057
139PD6Prags	*Polyarthra*	*dolichoptera*	PragserWildsee	08/10/2010	water column	46.698023000	12.086592000	50µm	KC619058
141PD4Napfen	*Polyarthra*	*dolichoptera*	Napfenlacke	08/07/2012	water column	46.973364000	12.122544000	50µm	KC619059
141PD5Napfen	*Polyarthra*	*dolichoptera*	Napfenlacke	08/07/2012	water column	46.973364000	12.122544000	50µm	KC619060
141PD6Napfen	*Polyarthra*	*dolichoptera*	Napfenlacke	08/07/2012	water column	46.973364000	12.122544000	50µm	KC619061
142PD6Tovel	*Polyarthra*	*dolichoptera*	Tovel	01/07/2009	water column	46.261402873	10.951116085	50µm	KC619026
143PD3Kaltern	*Polyarthra*	*dolichoptera*	Kaltern	28/07/2010	water column	46.38057016	11.2651062	50µm	KC619062
143PD4Kaltern	*Polyarthra*	*dolichoptera*	Kaltern	28/07/2010	water column	46.38057016	11.2651062	50µm	KC619063
143PD8Kaltern	*Polyarthra*	*dolichoptera*	Kaltern	28/07/2010	water column	46.38057016	11.2651062	50µm	KC619064
144PD1Madrano	*Polyarthra*	*dolichoptera*	Madrano	28/07/2010	water column	46.08582275	11.21888638	50µm	KC619065
144PD2Madrano	*Polyarthra*	*dolichoptera*	Madrano	28/07/2010	water column	46.08582275	11.21888638	50µm	KC619066
144PD3Madrano	*Polyarthra*	*dolichoptera*	Madrano	28/07/2010	water column	46.08582275	11.21888638	50µm	KC619067
144PD4Madrano	*Polyarthra*	*dolichoptera*	Madrano	28/07/2010	water column	46.08582275	11.21888638	50µm	KC619068
144PD5Madrano	*Polyarthra*	*dolichoptera*	Madrano	28/07/2010	water column	46.08582275	11.21888638	50µm	KC619069
144PD7Madrano	*Polyarthra*	*dolichoptera*	Madrano	28/07/2010	water column	46.08582275	11.21888638	50µm	KC619070
144PD8Madrano	*Polyarthra*	*dolichoptera*	Madrano	28/07/2010	water column	46.08582275	11.21888638	50µm	KC619071
148PD2Bodensee	*Polyarthra*	*dolichoptera*	Bodensee	18/07/2012	water column	47.49657475	9.465301037	50µm	KC619072
148PD3Bodensee	*Polyarthra*	*dolichoptera*	Bodensee	18/07/2012	water column	47.49657475	9.465301037	50µm	KC619073
148PD4Bodensee	*Polyarthra*	*dolichoptera*	Bodensee	18/07/2012	water column	47.49657475	9.465301037	50µm	KC619074
148PD5Bodensee	*Polyarthra*	*dolichoptera*	Bodensee	18/07/2012	water column	47.49657475	9.465301037	50µm	KC619075
148PD8Bodensee	*Polyarthra*	*dolichoptera*	Bodensee	18/07/2012	water column	47.49657475	9.465301037	50µm	KC619076
152PD1Haidersee	*Polyarthra*	*dolichoptera*	Haidersee	19/07/2012	water column	46.75938557	10.53503036	50µm	KC619077

152PD3Haidersee	*Polyarthra*	*dolichoptera*	Haidersee	19/07/2012	water column	46.75938557	10.53503036	50µm	KC619078
152PD4Haidersee	*Polyarthra*	*dolichoptera*	Haidersee	19/07/2012	water column	46.75938557	10.53503036	50µm	KC619079
152PD5Haidersee	*Polyarthra*	*dolichoptera*	Haidersee	19/07/2012	water column	46.75938557	10.53503036	50µm	KC619080
152PD6Haidersee	*Polyarthra*	*dolichoptera*	Haidersee	19/07/2012	water column	46.75938557	10.53503036	50µm	KC619081
152PD7Haidersee	*Polyarthra*	*dolichoptera*	Haidersee	19/07/2012	water column	46.75938557	10.53503036	50µm	KC619082
152PD7Tovel	*Polyarthra*	*dolichoptera*	Tovel	01/07/2009	water column	46.261402873	10.951116085	50µm	KC619027
152PD8Haidersee	*Polyarthra*	*dolichoptera*	Haidersee	19/07/2012	water column	46.75938557	10.53503036	50µm	KC619083
153PU7Donau	*Polyarthra*	*dolichoptera/vulgaris*	Donau	02/07/2012	water column	46.01111111	18.76694444	50µm	KC619084
153PU8Donau	*Polyarthra*	*vulgaris*	Donau	02/07/2012	water column	46.01111111	18.76694444	50µm	KC619085
154PD1Cembra	*Polyarthra*	*dolichoptera*	Donau	02/07/2012	water column	46.01111111	18.76694444	50µm	KC619086
154PD2Cembra	*Polyarthra*	*dolichoptera*	Cembra	28/07/2010	water column	46.19608182	11.20837212	50µm	KC619087
154PD3Cembra	*Polyarthra*	*dolichoptera*	Cembra	28/07/2010	water column	46.19608182	11.20837212	50µm	KC619088
154PD4Cembra	*Polyarthra*	*dolichoptera*	Cembra	28/07/2010	water column	46.19608182	11.20837212	50µm	KC619089
154PD7Cembra	*Polyarthra*	*dolichoptera*	Cembra	28/07/2010	water column	46.19608182	11.20837212	50µm	KC619090
154PD8Cembra	*Polyarthra*	*dolichoptera*	Cembra	28/07/2010	water column	46.19608182	11.20837212	50µm	KC619091
156PD1Caldonazzo	*Polyarthra*	*dolichoptera*	Caldonazzo	23/07/2010	water column	46.013296807	11.251716614	50µm	KC619092
156PD2Caldonazzo	*Polyarthra*	*dolichoptera*	Caldonazzo	23/07/2010	water column	46.013296807	11.251716614	50µm	KC619093
156PD3Caldonazzo	*Polyarthra*	*dolichoptera*	Caldonazzo	23/07/2010	water column	46.013296807	11.251716614	50µm	KC619094
158PD1Griessbachsee	*Polyarthra*	*dolichoptera*	Griessbachsee	18/07/2010	water column	47.05269804	12.04719543	50µm	KC619095
158PD2Griessbachsee	*Polyarthra*	*dolichoptera*	Griessbachsee	18/07/2010	water column	47.05269804	12.04719543	50µm	KC619096
159PD1Antholz	*Polyarthra*	*dolichoptera*	Antholz	10/08/2010	water column	46.8853042	12.16529846	50µm	KC619097
159PD2Antholz	*Polyarthra*	*dolichoptera*	Antholz	10/08/2010	water column	46.8853042	12.16529846	50µm	KC619098
159PD3Antholz	*Polyarthra*	*dolichoptera*	Antholz	10/08/2010	water column	46.8853042	12.16529846	50µm	KC619099
159PD4Antholz	*Polyarthra*	*dolichoptera*	Antholz	10/08/2010	water column	46.8853042	12.16529846	50µm	KC619100
159PD7Antholz	*Polyarthra*	*dolichoptera*	Antholz	10/08/2010	water column	46.8853042	12.16529846	50µm	KC619101
159PD8Antholz	*Polyarthra*	*dolichoptera*	Antholz	10/08/2010	water column	46.8853042	12.16529846	50µm	KC619102
160PD2Crespeina	*Polyarthra*	*dolichoptera*	Crespeina	29/08/2010	water column	46.56856789	11.82497978	50µm	KC619103
160PD3Crespeina	*Polyarthra*	*dolichoptera*	Crespeina	29/08/2010	water column	46.56856789	11.82497978	50µm	KC619104
160PD4Crespeina	*Polyarthra*	*dolichoptera*	Crespeina	28/08/2010	water column	46.56856789	11.82497978	50µm	KC619105
160PD5Crespeina	*Polyarthra*	*dolichoptera*	Crespeina	29/08/2010	water column	46.56856789	11.82497978	50µm	KC619106
160PD8Crespeina	*Polyarthra*	*dolichoptera*	Crespeina	29/08/2010	water column	46.56856789	11.82497978	50µm	KC619107
161PD4Durnholz	*Polyarthra*	*dolichoptera*	Durnholz	10/08/2010	water column	46.8853042	12.16529846	50µm	KC619108
161PD5Durnholz	*Polyarthra*	*dolichoptera*	Durnholz	10/08/2010	water column	46.8853042	12.16529846	50µm	KC619109
161PD6Durnholz	*Polyarthra*	*dolichoptera*	Durnholz	10/08/2010	water column	46.8853042	12.16529846	50µm	KC619110
162PD1Valle	*Polyarthra*	*dolichoptera*	Valle	28/07/2010	water column	46.12328445	11.21652603	50µm	KC619111
163PD2Fennberg	*Polyarthra*	*dolichoptera*	Fennberg	07/08/2010	water column	46.27453785	11.18476868	50µm	KC619112
163PD7Fennberg	*Polyarthra*	*dolichoptera*	Fennberg	07/08/2010	water column	46.27453785	11.18476868	50µm	KC619113
163PD8Fennberg	*Polyarthra*	*dolichoptera*	Fennberg	07/08/2010	water column	46.27453785	11.18476868	50µm	KC619114
164PD2Tenno	*Polyarthra*	*dolichoptera*	Tenno	04/08/2010	water column	45.93688538	10.81466675	50µm	KC619115
164PD4Tenno	*Polyarthra*	*dolichoptera*	Tenno	04/08/2010	water column	45.93688538	10.81466675	50µm	KC619116
164PD5Tenno	*Polyarthra*	*dolichoptera*	Tenno	04/08/2010	water column	45.93688538	10.81466675	50µm	KC619117
165PD4Ampola	*Polyarthra*	*vulgaris*	Ampola	06/08/2010	water column	45.87064857	10.65244675	50µm	KC619118
165PD8Ampola	*Polyarthra*	*vulgaris*	Ampola	06/08/2010	water column	45.87064857	10.65244675	50µm	KC619119
168PD1Tovel4m	*Polyarthra*	*dolichoptera*	Tovel	06/08/2012	5m	46.261402873	10.951116085	50µm	KC619120
168PD2Tovel4m	*Polyarthra*	*dolichoptera*	Tovel	06/08/2012	5m	46.261402873	10.951116085	50µm	KC619121
168PD3Tovel4m	*Polyarthra*	*dolichoptera*	Tovel	06/08/2012	5m	46.261402873	10.951116085	50µm	KC619122
168PD4Tovel4m	*Polyarthra*	*dolichoptera*	Tovel	06/08/2012	5m	46.261402873	10.951116085	50µm	KC619123
168PD5Tovel4m	*Polyarthra*	*dolichoptera*	Tovel	06/08/2012	5m	46.261402873	10.951116085	50µm	KC619124
168PD6Tovel4m	*Polyarthra*	*dolichoptera*	Tovel	06/08/2012	5m	46.261402873	10.951116085	50µm	KC619125
168PD7Tovel4m	*Polyarthra*	*dolichoptera*	Tovel	06/08/2012	5m	46.261402873	10.951116085	50µm	KC619126
168PD8Tovel4m	*Polyarthra*	*dolichoptera*	Tovel	06/08/2012	5m	46.261402873	10.951116085	50µm	KC619127
170PD2TovelB	*Polyarthra*	*dolichoptera*	Tovel	06/08/2012	water column	46.257211776	10.944786072	50µm	KC619128
170PD3TovelB	*Polyarthra*	*dolichoptera*	Tovel	06/08/2012	water column	46.257211776	10.944786072	50µm	KC619129
170PD4TovelB	*Polyarthra*	*dolichoptera*	Tovel	06/08/2012	water column	46.257211776	10.944786072	50µm	KC619130
170PD5TovelB	*Polyarthra*	*dolichoptera*	Tovel	06/08/2012	water column	46.257211776	10.944786072	50µm	KC619131
170PD6TovelB	*Polyarthra*	*dolichoptera*	Tovel	06/08/2012	water column	46.257211776	10.944786072	50µm	KC619132
170PD7TovelB	*Polyarthra*	*dolichoptera*	Tovel	06/08/2012	water column	46.257211776	10.944786072	50µm	KC619133
170PD8TovelB	*Polyarthra*	*dolichoptera*	Tovel	06/08/2012	water column	46.257211776	10.944786072	50µm	KC619134
171PU1TerlagoGR	*Polyarthra*	*dolichoptera*	Terlago	08/08/2012	water column	46.097102622	11.054906845	50µm	KC619298
171PU2TerlagoGR	*Polyarthra*	*dolichoptera*	Terlago	08/08/2012	water column	46.097102622	11.054906845	50µm	KC619299
171PU3TerlagoGR	*Polyarthra*	*dolichoptera*	Terlago	08/08/2012	water column	46.097102622	11.054906845	50µm	KC619300
171PU4TerlagoGR	*Polyarthra*	*dolichoptera*	Terlago	08/08/2012	water column	46.097102622	11.054906845	50µm	KC619301
171PU5TerlagoGR	*Polyarthra*	*dolichoptera*	Terlago	08/08/2012	water column	46.097102622	11.054906845	50µm	KC619302
171PU6TerlagoGR	*Polyarthra*	*dolichoptera*	Terlago	08/08/2012	water column	46.097102622	11.054906845	50µm	KC619303
172PU1TerlagoKL	*Polyarthra*	*dolichoptera*	Terlago	04/08/2010	water column	46.097102622	11.054906845	50µm	KC619304
172PU2TerlagoKL	*Polyarthra*	*dolichoptera*	Terlago	04/08/2010	water column	46.097102622	11.054906845	50µm	KC619305
172PU3TerlagoKL	*Polyarthra*	*dolichoptera*	Terlago	04/08/2010	water column	46.097102622	11.054906845	50µm	KC619306
172PU4TerlagoKL	*Polyarthra*	*dolichoptera*	Terlago	04/08/2010	water column	46.097102622	11.054906845	50µm	KC619307
172PU5TerlagoKL	*Polyarthra*	*dolichoptera*	Terlago	04/08/2010	water column	46.097102622	11.054906845	50µm	KC619308
173PD1TerlagoGR	*Polyarthra*	*dolichoptera*	Terlago	08/08/2012	water column	46.097102622	11.054906845	50µm	KC619280
173PD2TerlagoGR	*Polyarthra*	*dolichoptera*	Terlago	08/08/2012	water column	46.097102622	11.054906845	50µm	KC619281
173PD4TerlagoGR	*Polyarthra*	*dolichoptera*	Terlago	08/08/2012	water column	46.097102622	11.054906845	50µm	KC619282
173PD5TerlagoGR	*Polyarthra*	*dolichoptera*	Terlago	08/08/2012	water column	46.097102622	11.054906845	50µm	KC619283
173PD7TerlagoGR	*Polyarthra*	*dolichoptera*	Terlago	08/08/2012	water column	46.097102622	11.054906845	50µm	KC619284
174PD1TerlagoMI	*Polyarthra*	*dolichoptera*	Terlago	08/08/2012	water column	46.097102622	11.054906845	50µm	KC619285
174PD2TerlagoMI	*Polyarthra*	*dolichoptera*	Terlago	08/08/2012	water column	46.097102622	11.054906845	50µm	KC619286
174PD3TerlagoMI	*Polyarthra*	*vulgaris*	Terlago	08/08/2012	water column	46.097102622	11.054906845	50µm	KC619287
174PD4TerlagoMI	*Polyarthra*	*dolichoptera*	Terlago	08/08/2012	water column	46.097102622	11.054906845	50µm	KC619288
174PD5TerlagoMI	*Polyarthra*	*vulgaris*	Terlago	08/08/2012	water column	46.097102622	11.054906845	50µm	KC619289
175PD1TerlagoKL	*Polyarthra*	*dolichoptera*	Terlago	08/08/2012	water column	46.097102622	11.054906845	50µm	KC619290
175PD2TerlagoKL	*Polyarthra*	*dolichoptera*	Terlago	08/08/2012	water column	46.097102622	11.054906845	50µm	KC619291
175PD3TerlagoKL	*Polyarthra*	*dolichoptera*	Terlago	04/08/2010	water column	46.097102622	11.054906845	50µm	KC619292
175PD4TerlagoKL	*Polyarthra*	*dolichoptera*	Terlago	04/08/2010	water column	46.097102622	11.054906845	50µm	KC619293
175PD5TerlagoKL	*Polyarthra*	*dolichoptera*	Terlago	04/08/2010	water column	46.097102622	11.054906845	50µm	KC619294
175PD6TerlagoKL	*Polyarthra*	*dolichoptera*	Terlago	04/08/2010	water column	46.097102622	11.054906845	50µm	KC619295
175PD7TerlagoKL	*Polyarthra*	*dolichoptera*	Terlago	04/08/2010	water column	46.097102622	11.054906845	50µm	KC619296
175PD8TerlagoKL	*Polyarthra*	*dolichoptera*	Terlago	04/08/2010	water column	46.097102622	11.054906845	50µm	KC619297

178PD1Lamar	*Polyarthra*	*dolichoptera*	Lamar	08/08/2012	water column	46.12953055	11.06241703	50µm	KC619135
178PD2Lamar	*Polyarthra*	*dolichoptera*	Lamar	08/08/2012	water column	46.12953055	11.06241703	50µm	KC619136
178PD3Lamar	*Polyarthra*	*dolichoptera*	Lamar	08/08/2012	water column	46.12953055	11.06241703	50µm	KC619137
178PD5Lamar	*Polyarthra*	*dolichoptera*	Lamar	08/08/2012	water column	46.12953055	11.06241703	50µm	KC619138
178PD6Lamar	*Polyarthra*	*dolichoptera*	Lamar	08/08/2012	water column	46.12953055	11.06241703	50µm	KC619139
178PD7Lamar	*Polyarthra*	*dolichoptera*	Lamar	08/08/2012	water column	46.12953055	11.06241703	50µm	KC619140
178PD8Lamar	*Polyarthra*	*dolichoptera*	Lamar	08/08/2012	water column	46.12953055	11.06241703	50µm	KC619141
17PD1Caldonazzo	*Polyarthra*	*dolichoptera*	Caldonazzo	18/01/2011	water column	46.013296807	11.251716614	50µm	JN936503
17PD3Caldonazzo	*Polyarthra*	*dolichoptera*	Caldonazzo	18/01/2011	water column	46.013296807	11.251716614	50µm	JN936504
17PD4Caldonazzo	*Polyarthra*	*dolichoptera*	Caldonazzo	18/01/2011	water column	46.013296807	11.251716614	50µm	JN936505
17PD6Caldonazzo	*Polyarthra*	*dolichoptera*	Caldonazzo	18/01/2011	water column	46.013296807	11.251716614	50µm	JN936506
181PD1Tenno	*Polyarthra*	*dolichoptera*	Tenno	20/08/2012	water column	45.93688538	10.81466675	50µm	KC619142
181PD2Tenno	*Polyarthra*	*dolichoptera*	Tenno	20/08/2012	water column	45.93688538	10.81466675	50µm	KC619143
181PD3Tenno	*Polyarthra*	*dolichoptera*	Tenno	20/08/2012	water column	45.93688538	10.81466675	50µm	KC619144
181PD4Tenno	*Polyarthra*	*dolichoptera*	Tenno	20/08/2012	water column	45.93688538	10.81466675	50µm	KC619145
181PD5Tenno	*Polyarthra*	*dolichoptera*	Tenno	20/08/2012	water column	45.93688538	10.81466675	50µm	KC619146
181PD6Tenno	*Polyarthra*	*dolichoptera*	Tenno	20/08/2012	water column	45.93688538	10.81466675	50µm	KC619147
181PD7Tenno	*Polyarthra*	*dolichoptera*	Tenno	20/08/2012	water column	45.93688538	10.81466675	50µm	KC619148
181PD8Tenno	*Polyarthra*	*dolichoptera*	Tenno	20/08/2012	water column	45.93688538	10.81466675	50µm	KC619149
183PD1Valle	*Polyarthra*	*dolichoptera*	Valle	20/08/2012	water column	46.12328445	11.21652603	50µm	KC619150
183PD3Valle	*Polyarthra*	*dolichoptera*	Valle	20/08/2012	water column	46.12328445	11.21652603	50µm	KC619151
183PD6Valle	*Polyarthra*	*dolichoptera*	Valle	20/08/2012	water column	46.12328445	11.21652603	50µm	KC619152
183PD7Valle	*Polyarthra*	*dolichoptera*	Valle	20/08/2012	water column	46.12328445	11.21652603	50µm	KC619153
183PD8Valle	*Polyarthra*	*dolichoptera*	Valle	20/08/2012	water column	46.12328445	11.21652603	50µm	KC619154
184PD1Lases	*Polyarthra*	*dolichoptera*	Lases	20/08/2012	water column	46.141782738	11.219186783	50µm	KC619155
184PD2Lases	*Polyarthra*	*dolichoptera*	Lases	20/08/2012	water column	46.141782738	11.219186783	50µm	KC619156
184PD3Lases	*Polyarthra*	*dolichoptera*	Lases	20/08/2012	water column	46.141782738	11.219186783	50µm	KC619157
184PD4Lases	*Polyarthra*	*dolichoptera*	Lases	20/08/2012	water column	46.141782738	11.219186783	50µm	KC619158
184PD5Lases	*Polyarthra*	*dolichoptera*	Lases	20/08/2012	water column	46.141782738	11.219186783	50µm	KC619159
184PD7Lases	*Polyarthra*	*dolichoptera*	Lases	20/08/2012	water column	46.141782738	11.219186783	50µm	KC619160
184PD8Lases	*Polyarthra*	*dolichoptera*	Lases	20/08/2012	water column	46.141782738	11.219186783	50µm	KC619161
186PD1Ampola	*Polyarthra*	*vulgaris*	Ampola	20/08/2012	water column	45.87064857	10.65244675	50µm	KC619194
186PD3Ampola	*Polyarthra*	*vulgaris*	Ampola	20/08/2012	water column	45.87064857	10.65244675	50µm	KC619195
186PD4Ampola	*Polyarthra*	*vulgaris*	Ampola	20/08/2012	water column	45.87064857	10.65244675	50µm	KC619196
186PD5Ampola	*Polyarthra*	*vulgaris*	Ampola	20/08/2012	water column	45.87064857	10.65244675	50µm	KC619197
186PD8Ampola	*Polyarthra*	*vulgaris*	Ampola	20/08/2012	water column	45.87064857	10.65244675	50µm	KC619199
188PD1Madrano	*Polyarthra*	*vulgaris*	Madrano	20/08/2012	water column	46.08582275	11.21888638	50µm	KC619200
188PD2Madrano	*Polyarthra*	*vulgaris*	Madrano	20/08/2012	water column	46.08582275	11.21888638	50µm	KC619201
188PD3Madrano	*Polyarthra*	*vulgaris*	Madrano	20/08/2012	water column	46.08582275	11.21888638	50µm	KC619202
188PD4Madrano	*Polyarthra*	*vulgaris*	Madrano	20/08/2012	water column	46.08582275	11.21888638	50µm	KC619203
188PD7Madrano	*Polyarthra*	*vulgaris*	Madrano	20/08/2012	water column	46.08582275	11.21888638	50µm	KC619204
188PD8Madrano	*Polyarthra*	*vulgaris*	Madrano	20/08/2012	water column	46.08582275	11.21888638	50µm	KC619205
190PD2Serraia	*Polyarthra*	*dolichoptera*	Serraia	20/08/2012	water column	46.136370698	11.251115799	50µm	KC619162
190PD3Serraia	*Polyarthra*	*dolichoptera*	Serraia	20/08/2012	water column	46.136370698	11.251115799	50µm	KC619163
190PD4Serraia	*Polyarthra*	*dolichoptera*	Serraia	20/08/2012	water column	46.136370698	11.251115799	50µm	KC619164
190PD5Serraia	*Polyarthra*	*dolichoptera*	Serraia	20/08/2012	water column	46.136370698	11.251115799	50µm	KC619165
190PD6Serraia	*Polyarthra*	*dolichoptera*	Serraia	20/08/2012	water column	46.136370698	11.251115799	50µm	KC619166
190PD7Serraia	*Polyarthra*	*dolichoptera*	Serraia	20/08/2012	water column	46.136370698	11.251115799	50µm	KC619167
190PD8Serraia	*Polyarthra*	*dolichoptera*	Serraia	20/08/2012	water column	46.136370698	11.251115799	50µm	KC619168
191PD1Lavarone	*Polyarthra*	*dolichoptera*	Lavarone	20/08/2012	water column	45.937541974	11.250040741	50µm	KC619169
191PD2Lavarone	*Polyarthra*	*dolichoptera*	Lavarone	20/08/2012	water column	45.937541974	11.250040741	50µm	KC619170
191PD5Lavarone	*Polyarthra*	*dolichoptera*	Lavarone	20/08/2012	water column	45.937541974	11.250040741	50µm	KC619171
191PD6Lavarone	*Polyarthra*	*dolichoptera*	Lavarone	20/08/2012	water column	45.937541974	11.250040741	50µm	KC619172
191PD7Lavarone	*Polyarthra*	*dolichoptera*	Lavarone	20/08/2012	water column	45.937541974	11.250040741	50µm	KC619173
191PD8Lavarone	*Polyarthra*	*dolichoptera*	Lavarone	20/08/2012	water column	45.937541974	11.250040741	50µm	KC619174
192PD1Cei	*Polyarthra*	*dolichoptera*	Cei	20/08/2012	water column	45.952373024	11.019973755	50µm	KC619175
192PD2Cei	*Polyarthra*	*dolichoptera*	Cei	20/08/2012	water column	45.952373024	11.019973755	50µm	KC619176
192PD3Cei	*Polyarthra*	*dolichoptera*	Cei	20/08/2012	water column	45.952373024	11.019973755	50µm	KC619177
192PD4Cei	*Polyarthra*	*dolichoptera*	Cei	20/08/2012	water column	45.952373024	11.019973755	50µm	KC619178
192PD5Cei	*Polyarthra*	*dolichoptera*	Cei	20/08/2012	water column	45.952373024	11.019973755	50µm	KC619179
192PD6Cei	*Polyarthra*	*dolichoptera*	Cei	20/08/2012	water column	45.952373024	11.019973755	50µm	KC619180
192PD7Cei	*Polyarthra*	*dolichoptera*	Cei	20/08/2012	water column	45.952373024	11.019973755	50µm	KC619181
192PD8Cei	*Polyarthra*	*dolichoptera*	Cei	20/08/2012	water column	45.952373024	11.019973755	50µm	KC619182
194PD1Caldonazzo	*Polyarthra*	*dolichoptera*	Caldonazzo	20/08/2012	water column	46.013296807	11.251716614	50µm	KC619183
194PD3Caldonazzo	*Polyarthra*	*dolichoptera*	Caldonazzo	20/08/2012	water column	46.013296807	11.251716614	50µm	KC619184
194PD5Caldonazzo	*Polyarthra*	*dolichoptera*	Caldonazzo	20/08/2012	water column	46.013296807	11.251716614	50µm	KC619185
194PD7Caldonazzo	*Polyarthra*	*dolichoptera*	Caldonazzo	20/08/2012	water column	46.013296807	11.251716614	50µm	KC619186
194PD8Caldonazzo	*Polyarthra*	*dolichoptera*	Caldonazzo	20/08/2012	water column	46.013296807	11.251716614	50µm	KC619187
196PD1Canzolino	*Polyarthra*	*dolichoptera*	Canzolino	20/08/2012	water column	46.082875918	11.225795746	50µm	KC619188
196PD2Canzolino	*Polyarthra*	*dolichoptera*	Canzolino	20/08/2012	water column	46.082875918	11.225795746	50µm	KC619189
196PD3Canzolino	*Polyarthra*	*dolichoptera*	Canzolino	20/08/2012	water column	46.082875918	11.225795746	50µm	KC619190
196PD5Canzolino	*Polyarthra*	*dolichoptera*	Canzolino	20/08/2012	water column	46.082875918	11.225795746	50µm	KC619191
196PD6Canzolino	*Polyarthra*	*dolichoptera*	Canzolino	20/08/2012	water column	46.082875918	11.225795746	50µm	KC619192
196PD7Canzolino	*Polyarthra*	*dolichoptera*	Canzolino	20/08/2012	water column	46.082875918	11.225795746	50µm	KC619193
199PD1Vahrn	*Polyarthra*	*dolichoptera*	VahrnerSee	02/09/2012	water column	46.766029498	11.635036485	50µm	KC619206
199PD2Vahrn	*Polyarthra*	*dolichoptera*	VahrnerSee	02/09/2012	water column	46.766029498	11.635036485	50µm	KC619207
199PD3Vahrn	*Polyarthra*	*dolichoptera*	VahrnerSee	02/09/2012	water column	46.766029498	11.635036485	50µm	KC619208
199PD4Vahrn	*Polyarthra*	*dolichoptera*	VahrnerSee	02/09/2012	water column	46.766029498	11.635036485	50µm	KC619209
199PD5Vahrn	*Polyarthra*	*dolichoptera*	VahrnerSee	02/09/2012	water column	46.766029498	11.635036485	50µm	KC619210
199PD6Vahrn	*Polyarthra*	*dolichoptera*	VahrnerSee	02/09/2012	water column	46.766029498	11.635036485	50µm	KC619211
199PD7Vahrn	*Polyarthra*	*dolichoptera*	VahrnerSee	02/09/2012	water column	46.766029498	11.635036485	50µm	KC619212
199PD8Vahrn	*Polyarthra*	*dolichoptera*	VahrnerSee	02/09/2012	water column	46.766029498	11.635036485	50µm	KC619213
200PD1Raier	*Polyarthra*	*dolichoptera*	Raiermoos	02/09/2012	water column	46.746227574	11.664562225	50µm	KC619214
200PD2Raier	*Polyarthra*	*dolichoptera*	Raiermoos	02/09/2012	water column	46.746227574	11.664562225	50µm	KC619215
200PD3Raier	*Polyarthra*	*dolichoptera*	Raiermoos	02/09/2012	water column	46.746227574	11.664562225	50µm	KC619216
200PD4Raier	*Polyarthra*	*dolichoptera*	Raiermoos	02/09/2012	water column	46.746227574	11.664562225	50µm	KC619217
200PD5Raier	*Polyarthra*	*dolichoptera*	Raiermoos	02/09/2012	water column	46.746227574	11.664562225	50µm	KC619218

200PD6Raier	*Polyarthra*	*dolichoptera*	Raiermoos	02/09/2012	water column	46.746227574	11.664562225	50µm	KC619219
200PD7Raier	*Polyarthra*	*dolichoptera*	Raiermoos	02/09/2012	water column	46.746227574	11.664562225	50µm	KC619220
200PD8Raier	*Polyarthra*	*dolichoptera*	Raiermoos	02/09/2012	water column	46.746227574	11.664562225	50µm	KC619221
202PD1Eisbrugg	*Polyarthra*	*dolichoptera*	EisbruggSee	09/09/2012	water column	46.940125064	11.729793549	50µm	KC619222
202PD2Eisbrugg	*Polyarthra*	*dolichoptera*	EisbruggSee	09/09/2012	water column	46.940125064	11.729793549	50µm	KC619223
202PD3Eisbrugg	*Polyarthra*	*dolichoptera*	EisbruggSee	09/09/2012	water column	46.940125064	11.729793549	50µm	KC619224
202PD4Eisbrugg	*Polyarthra*	*dolichoptera*	EisbruggSee	09/09/2012	water column	46.940125064	11.729793549	50µm	KC619225
202PD5Eisbrugg	*Polyarthra*	*dolichoptera*	EisbruggSee	09/09/2012	water column	46.940125064	11.729793549	50µm	KC619226
202PD7Eisbrugg	*Polyarthra*	*dolichoptera*	EisbruggSee	09/09/2012	water column	46.940125064	11.729793549	50µm	KC619227
203PD1Tovel4m	*Polyarthra*	*dolichoptera*	Tovel	10/09/2012	5m	46.261402873	10.951116085	50µm	KC619228
203PD2Tovel4m	*Polyarthra*	*dolichoptera*	Tovel	10/09/2012	5m	46.261402873	10.951116085	50µm	KC619229
203PD3Tovel4m	*Polyarthra*	*dolichoptera*	Tovel	10/09/2012	5m	46.261402873	10.951116085	50µm	KC619230
203PD4Tovel4m	*Polyarthra*	*dolichoptera*	Tovel	10/09/2012	5m	46.261402873	10.951116085	50µm	KC619231
203PD5Tovel4m	*Polyarthra*	*dolichoptera*	Tovel	10/09/2012	5m	46.261402873	10.951116085	50µm	KC619232
203PD6Tovel4m	*Polyarthra*	*dolichoptera*	Tovel	10/09/2012	5m	46.261402873	10.951116085	50µm	KC619233
203PD7Tovel4m	*Polyarthra*	*dolichoptera*	Tovel	10/09/2012	5m	46.261402873	10.951116085	50µm	KC619234
204PD1Tovel15m	*Polyarthra*	*dolichoptera*	Tovel	10/09/2012	15m	46.261402873	10.951116085	50µm	KC619235
204PD2Tovel15m	*Polyarthra*	*dolichoptera*	Tovel	10/09/2012	15m	46.261402873	10.951116085	50µm	KC619236
204PD4Tovel15m	*Polyarthra*	*dolichoptera*	Tovel	10/09/2012	15m	46.261402873	10.951116085	50µm	KC619237
204PD5Tovel15m	*Polyarthra*	*dolichoptera*	Tovel	10/09/2012	15m	46.261402873	10.951116085	50µm	KC619238
204PD6Tovel15m	*Polyarthra*	*dolichoptera*	Tovel	10/09/2012	15m	46.257211776	10.944786072	50µm	KC619239
206PD1TovelB	*Polyarthra*	*dolichoptera*	Tovel	10/09/2012	water column	46.257211776	10.944786072	50µm	KC619240
206PD3TovelB	*Polyarthra*	*dolichoptera*	Tovel	10/09/2012	water column	46.257211776	10.944786072	50µm	KC619241
206PD4TovelB	*Polyarthra*	*dolichoptera*	Tovel	10/09/2012	water column	46.257211776	10.944786072	50µm	KC619242
206PD5TovelB	*Polyarthra*	*dolichoptera*	Tovel	10/09/2012	water column	46.257211776	10.944786072	50µm	KC619243
206PD6TovelB	*Polyarthra*	*dolichoptera*	Tovel	10/09/2012	water column	46.257211776	10.944786072	50µm	KC619244
206PD7TovelB	*Polyarthra*	*dolichoptera*	Tovel	10/09/2012	water column	46.257211776	10.944786072	50µm	KC619245
206PD8TovelB	*Polyarthra*	*dolichoptera*	Tovel	10/09/2012	water column	46.257211776	10.944786072	50µm	KC619246
207PD1WilderSee	*Polyarthra*	*dolichoptera*	WilderSee	16/09/2012	water column	46.898208270	11.588859500	50µm	KC619247
207PD2WilderSee	*Polyarthra*	*dolichoptera*	WilderSee	16/09/2012	water column	46.898208270	11.588859500	50µm	KC619248
207PD4WilderSee	*Polyarthra*	*dolichoptera*	WilderSee	16/09/2012	water column	46.898208270	11.588859500	50µm	KC619249
207PD5WilderSee	*Polyarthra*	*dolichoptera*	WilderSee	16/09/2012	water column	46.898208270	11.588859500	50µm	KC619250
207PD7WilderSee	*Polyarthra*	*dolichoptera*	WilderSee	16/09/2012	water column	46.898208270	11.588859500	50µm	KC619251
207PD8WilderSee	*Polyarthra*	*dolichoptera*	WilderSee	16/09/2012	water column	46.898208270	11.588859500	50µm	KC619252
209PD1Durst	*Polyarthra*	*dolichoptera*	Durst	16/09/2012	water column	46.715488581	11.665484905	50µm	KC619253
209PD2Durst	*Polyarthra*	*dolichoptera*	Durst	16/09/2012	water column	46.715488581	11.665484905	50µm	KC619254
209PD3Durst	*Polyarthra*	*dolichoptera*	Durst	16/09/2012	water column	46.715488581	11.665484905	50µm	KC619255
209PD4Durst	*Polyarthra*	*dolichoptera*	Durst	16/09/2012	water column	46.715488581	11.665484905	50µm	KC619256
209PD5Durst	*Polyarthra*	*dolichoptera*	Durst	16/09/2012	water column	46.715488581	11.665484905	50µm	KC619257
209PD7Durst	*Polyarthra*	*dolichoptera*	Durst	16/09/2012	water column	46.715488581	11.665484905	50µm	KC619258
209PD8Durst	*Polyarthra*	*dolichoptera*	Durst	16/09/2012	water column	46.715488581	11.665484905	50µm	KC619259
213PD1ColBriconInf	*Polyarthra*	*dolichoptera*	ColBricon, Inf	27/09/2012	water column	46.2838958	11.76605701	50µm	KC619260
213PD2ColBriconInf	*Polyarthra*	*dolichoptera*	ColBricon, Inf	27/09/2012	water column	46.2838958	11.76605701	50µm	KC619261
213PD3ColBriconInf	*Polyarthra*	*dolichoptera*	ColBricon, Inf	27/09/2012	water column	46.2838958	11.76605701	50µm	KC619262
213PD5ColBriconInf	*Polyarthra*	*dolichoptera*	ColBricon, Inf	27/09/2012	water column	46.2838958	11.76605701	50µm	KC619263
213PD6ColBriconInf	*Polyarthra*	*dolichoptera*	ColBricon, Inf	27/09/2012	water column	46.2838958	11.76605701	50µm	KC619264
213PD7ColBriconInf	*Polyarthra*	*dolichoptera*	ColBricon, Inf	27/09/2012	water column	46.2838958	11.76605701	50µm	KC619265
213PD8ColBriconInf	*Polyarthra*	*dolichoptera*	ColBricon, Inf	27/09/2012	water column	46.2838958	11.76605701	50µm	KC619266
214PD1Paneveggio	*Polyarthra*	*dolichoptera*	Paneveggio	27/09/2012	water column	46.30022019	11.73090935	50µm	KC619267
214PD2Paneveggio	*Polyarthra*	*dolichoptera*	Paneveggio	27/09/2012	water column	46.30022019	11.73090935	50µm	KC619268
214PD3Paneveggio	*Polyarthra*	*dolichoptera*	Paneveggio	27/09/2012	water column	46.30022019	11.73090935	50µm	KC619269
214PD4Paneveggio	*Polyarthra*	*dolichoptera*	Paneveggio	27/09/2012	water column	46.30022019	11.73090935	50µm	KC619270
214PD5Paneveggio	*Polyarthra*	*dolichoptera*	Paneveggio	27/09/2012	water column	46.30022019	11.73090935	50µm	KC619271
214PD6Paneveggio	*Polyarthra*	*dolichoptera*	Paneveggio	27/09/2012	water column	46.30022019	11.73090935	50µm	KC619272
214PD7Paneveggio	*Polyarthra*	*dolichoptera*	Paneveggio	27/09/2012	water column	46.30022019	11.73090935	50µm	KC619273
217PD1TovelB	*Polyarthra*	*dolichoptera*	Tovel	02/10/2012	water column	46.257211776	10.944786072	50µm	KC619274
217PD2TovelB	*Polyarthra*	*dolichoptera*	Tovel	02/10/2012	water column	46.257211776	10.944786072	50µm	KC619275
217PD3TovelB	*Polyarthra*	*dolichoptera*	Tovel	02/10/2012	water column	46.257211776	10.944786072	50µm	KC619276
217PD6TovelB	*Polyarthra*	*dolichoptera*	Tovel	02/10/2012	water column	46.257211776	10.944786072	50µm	KC619277
217PD7TovelB	*Polyarthra*	*dolichoptera*	Tovel	02/10/2012	water column	46.257211776	10.944786072	50µm	KC619278
217PD8TovelB	*Polyarthra*	*dolichoptera*	Tovel	02/10/2012	water column	46.257211776	10.944786072	50µm	KC619279
253PD1Canzolino	*Polyarthra*	*dolichoptera*	Canzolino	01/07/2009	water column	46.082875918	11.225795746	50µm	KC618942
283PD4Canzolino	*Polyarthra*	*dolichoptera*	Canzolino	01/07/2009	water column	46.082875918	11.225795746	50µm	KC618943
2PD1Lases	*Polyarthra*	*dolichoptera*	Lases	30/11/2010	water column	46.141782738	11.219186783	50µm	JN936494
2PD2Lases	*Polyarthra*	*dolichoptera*	Lases	30/11/2010	water column	46.141782738	11.219186783	50µm	JN936495
2PD3Lases	*Polyarthra*	*dolichoptera*	Lases	30/11/2010	water column	46.141782738	11.219186783	50µm	JN936496
2PD4Lases	*Polyarthra*	*dolichoptera*	Lases	30/11/2010	water column	46.141782738	11.219186783	50µm	JN936497
2PD5Lases	*Polyarthra*	*dolichoptera*	Lases	30/11/2010	water column	46.141782738	11.219186783	50µm	JN936498
2PD6Lases	*Polyarthra*	*dolichoptera*	Lases	30/11/2010	water column	46.141782738	11.219186783	50µm	JN936508
2PD7Lases	*Polyarthra*	*dolichoptera*	Lases	30/11/2010	water column	46.141782738	11.219186783	50µm	JN936507
2PD8Lases	*Polyarthra*	*dolichoptera*	Lases	30/11/2010	water column	46.141782738	11.219186783	50µm	JN936499
45PD5Kofelrast	*Polyarthra*	*dolichoptera*	Kofelrast	10/07/2011	water column	46.578155743	10.937662125	50µm	JN936500
48.1.PolyPresena	*Polyarthra*	*dolichoptera*	Presena	30/08/2011	water column	46.236377641	10.581808090	50µm	KC618866
48.10.PolyPresena	*Polyarthra*	*dolichoptera*	Presena	30/08/2011	water column	46.236377641	10.581808090	50µm	KC618873
48.11.PolyPresena	*Polyarthra*	*dolichoptera*	Presena	30/08/2011	water column	46.236377641	10.581808090	50µm	KC618874
48.12.PolyPresena	*Polyarthra*	*dolichoptera*	Presena	30/08/2011	water column	46.236377641	10.581808090	50µm	KC618875
48.3.PolyPresena	*Polyarthra*	*dolichoptera*	Presena	30/08/2011	water column	46.236377641	10.581808090	50µm	KC618867
48.4.PolyPresena	*Polyarthra*	*dolichoptera*	Presena	30/08/2011	water column	46.236377641	10.581808090	50µm	KC618868
48.5.PolyPresena	*Polyarthra*	*dolichoptera*	Presena	30/08/2011	water column	46.236377641	10.581808090	50µm	KC618869
48.6.PolyPresena	*Polyarthra*	*dolichoptera*	Presena	30/08/2011	water column	46.236377641	10.581808090	50µm	KC618870
48.7.PolyPresena	*Polyarthra*	*dolichoptera*	Presena	30/08/2011	water column	46.236377641	10.581808090	50µm	KC618876
48.8.PolyPresena	*Polyarthra*	*dolichoptera*	Presena	30/08/2011	water column	46.236377641	10.581808090	50µm	KC618871
48.9.PolyPresena	*Polyarthra*	*dolichoptera*	Presena	30/08/2011	water column	46.236377641	10.581808090	50µm	KC618872
49.10.PolyLagazuo	*Polyarthra*	*dolichoptera*	Lagazuoi	03/09/2011	water column	46.551453044	12.005728483	50µm	KC618901
49.1HPolyLagazuoi	*Polyarthra*	*dolichoptera*	Lagazuoi	03/09/2011	water column	46.551453044	12.005728483	50µm	KC618923
49.2.PolyLagazuoi	*Polyarthra*	*dolichoptera*	Lagazuoi	03/09/2011	water column	46.551453044	12.005728483	50µm	KC618899
49.3.PolyLagazuoi	*Polyarthra*	*dolichoptera*	Lagazuoi	03/09/2011	water column	46.551453044	12.005728483	50µm	KC618897

49.4.PolyLagazuoi	*Polyarthra*	*dolichoptera*	Lagazuoi	03/09/2011	water column	46.551453044	12.005728483	50µm	KC618905
49.5.PolyLagazuoi	*Polyarthra*	*dolichoptera*	Lagazuoi	03/09/2011	water column	46.551453044	12.005728483	50µm	KC618898
49.6.PolyLagazuoi	*Polyarthra*	*dolichoptera*	Lagazuoi	03/09/2011	water column	46.551453044	12.005728483	50µm	KC618904
49.7.PolyLagazuoi	*Polyarthra*	*dolichoptera*	Lagazuoi	03/09/2011	water column	46.551453044	12.005728483	50µm	KC618903
49.8.PolyLagazuoi	*Polyarthra*	*dolichoptera*	Lagazuoi	03/09/2011	water column	46.551453044	12.005728483	50µm	KC618902
49.9.PolyLagazuoi	*Polyarthra*	*dolichoptera*	Lagazuoi	03/09/2011	water column	46.551453044	12.005728483	50µm	KC618900
50.1.PolyVahrn	*Polyarthra*	*dolichoptera*	VahrnerSee	04/09/2011	water column	46.766029498	11.635036485	50µm	KC618932
50.10.PolyVahrn	*Polyarthra*	*dolichoptera*	VahrnerSee	04/09/2011	water column	46.766029498	11.635036485	50µm	KC618924
50.2.PolyVahrn	*Polyarthra*	*dolichoptera*	VahrnerSee	04/09/2011	water column	46.766029498	11.635036485	50µm	KC618931
50.3.PolyVahrn	*Polyarthra*	*dolichoptera*	VahrnerSee	04/09/2011	water column	46.766029498	11.635036485	50µm	KC618930
50.4.PolyVahrn	*Polyarthra*	*dolichoptera*	VahrnerSee	04/09/2011	water column	46.766029498	11.635036485	50µm	KC618929
50.5.PolyVahrn	*Polyarthra*	*dolichoptera*	VahrnerSee	04/09/2011	water column	46.766029498	11.635036485	50µm	KC618933
50.6.PolyVahrn	*Polyarthra*	*dolichoptera*	VahrnerSee	04/09/2011	water column	46.766029498	11.635036485	50µm	KC618928
50.7.PolyVahrn	*Polyarthra*	*dolichoptera*	VahrnerSee	04/09/2011	water column	46.766029498	11.635036485	50µm	KC618927
50.8.PolyVahrn	*Polyarthra*	*dolichoptera*	VahrnerSee	04/09/2011	water column	46.766029498	11.635036485	50µm	KC618926
50.9.PolyVahrn	*Polyarthra*	*dolichoptera*	VahrnerSee	04/09/2011	water column	46.766029498	11.635036485	50µm	KC618934
52.1.PolyTovel	*Polyarthra*	*dolichoptera*	Tovel	06/09/2011	water column	46.261402873	10.951116085	50µm	KC618921
52.10.PolyTovel	*Polyarthra*	*dolichoptera*	Tovel	06/09/2011	water column	46.257211776	10.944786072	50µm	KC618919
52.11.PolyTovel	*Polyarthra*	*dolichoptera*	Tovel	06/09/2011	water column	46.257211776	10.944786072	50µm	KC618918
52.13.PolyTovel	*Polyarthra*	*dolichoptera*	Tovel	06/09/2011	water column	46.257211776	10.944786072	50µm	KC618917
52.14.PolyTovel	*Polyarthra*	*dolichoptera*	Tovel	06/09/2011	water column	46.261402873	10.951116085	50µm	KC618922
52.15.PolyTovel	*Polyarthra*	*dolichoptera*	Tovel	06/09/2011	water column	46.257211776	10.944786072	50µm	KC618916
52.16.PolyTovel	*Polyarthra*	*dolichoptera*	Tovel	06/09/2011	water column	46.257211776	10.944786072	50µm	KC618915
52.2.PolyTovel	*Polyarthra*	*dolichoptera*	Tovel	06/09/2011	water column	46.261402873	10.951116085	50µm	KC619001
52.3.PolyTovel	*Polyarthra*	*dolichoptera*	Tovel	06/09/2011	water column	46.261402873	10.951116085	50µm	KC619002
52.4.PolyTovel	*Polyarthra*	*dolichoptera*	VahrnerSee	04/09/2011	water column	46.766029498	11.635036485	50µm	KC618925
52.5.PolyTovel	*Polyarthra*	*dolichoptera*	Tovel	06/09/2011	water column	46.261402873	10.951116085	50µm	KC619003
52.6.PolyTovel	*Polyarthra*	*dolichoptera*	Tovel	06/09/2011	water column	46.261402873	10.951116085	50µm	KC619004
52.7.PolyTovel	*Polyarthra*	*dolichoptera*	Tovel	06/09/2011	water column	46.261402873	10.951116085	50µm	KC619005
52.8.PolyTovel	*Polyarthra*	*dolichoptera*	Tovel	06/09/2011	water column	46.261402873	10.951116085	50µm	KC619006
52.9.PolyTovel	*Polyarthra*	*dolichoptera*	Tovel	06/09/2011	water column	46.257211776	10.944786072	50µm	KC618920
53.1.PolySerraia	*Polyarthra*	*dolichoptera*	Serraia	22/09/2011	water column	46.136370698	11.251115799	50µm	KC618939
53.2.PolySerraia	*Polyarthra*	*dolichoptera*	Serraia	22/09/2011	water column	46.136370698	11.251115799	50µm	KC618940
53.3.PolySerraia	*Polyarthra*	*dolichoptera*	Serraia	22/09/2011	water column	46.136370698	11.251115799	50µm	KC618935
53.4.PolySerraia	*Polyarthra*	*dolichoptera*	Serraia	22/09/2011	water column	46.136370698	11.251115799	50µm	KC618936
53.5.PolySerraia	*Polyarthra*	*dolichoptera*	Serraia	22/09/2011	water column	46.136370698	11.251115799	50µm	KC618941
53.6.PolySerraia	*Polyarthra*	*dolichoptera*	Serraia	22/09/2011	water column	46.136370698	11.251115799	50µm	KC618937
53.7.PolySerraia	*Polyarthra*	*dolichoptera*	Serraia	22/09/2011	water column	46.136370698	11.251115799	50µm	KC618938
53.8.PolySerraia	*Polyarthra*	*dolichoptera*	Serraia	22/09/2011	water column	46.136370698	11.251115799	50µm	KC618944
58.4.PolyCei	*Polyarthra*	*dolichoptera*	Cei	28/09/2011	water column	45.952373024	11.019973755	50µm	KC619028
58.5.PolyCei	*Polyarthra*	*dolichoptera*	Cei	28/09/2011	water column	45.952373024	11.019973755	50µm	KC619030
58.6.PolyCei	*Polyarthra*	*dolichoptera*	Cei	28/09/2011	water column	45.952373024	11.019973755	50µm	KC618896
58.7.PolyCei	*Polyarthra*	*dolichoptera*	Cei	28/09/2011	water column	45.952373024	11.019973755	50µm	KC619029
59PD2TovelA	*Polyarthra*	*dolichoptera*	Tovel	04/10/2011	water column	46.261402873	10.951116085	50µm	KC618914
59PD3TovelA	*Polyarthra*	*dolichoptera*	Tovel	04/10/2011	water column	46.261402873	10.951116085	50µm	KC618913
59PD4TovelA	*Polyarthra*	*dolichoptera*	Tovel	04/10/2011	water column	46.261402873	10.951116085	50µm	KC618912
59PD5TovelA	*Polyarthra*	*dolichoptera*	Tovel	04/10/2011	water column	46.261402873	10.951116085	50µm	KC618911
59PD6TovelA	*Polyarthra*	*dolichoptera*	Tovel	04/10/2011	water column	46.261402873	10.951116085	50µm	KC618910
59PD7TovelA	*Polyarthra*	*dolichoptera*	Tovel	04/10/2011	water column	46.261402873	10.951116085	50µm	KC618909
59PD8TovelA	*Polyarthra*	*dolichoptera*	Tovel	04/10/2011	water column	46.261402873	10.951116085	50µm	KC618908
60PD11TovelA	*Polyarthra*	*dolichoptera*	Tovel	05/04/2010	water column	46.261402873	10.951116085	50µm	KC618877
61PD1TovelA	*Polyarthra*	*dolichoptera*	Tovel	18/05/2010	water column	46.261402873	10.951116085	50µm	KC618878
62PD2TovelA	*Polyarthra*	*dolichoptera*	Tovel	27/02/2012	water column	46.261402873	10.951116085	50µm	KC619007
62PD3TovelA	*Polyarthra*	*dolichoptera*	Tovel	27/02/2012	water column	46.261402873	10.951116085	50µm	KC619008
62PD4TovelA	*Polyarthra*	*dolichoptera*	Tovel	27/02/2012	water column	46.261402873	10.951116085	50µm	KC619009
62PD6TovelA	*Polyarthra*	*dolichoptera*	Tovel	27/02/2012	water column	46.261402873	10.951116085	50µm	KC619025
62PD7TovelA	*Polyarthra*	*dolichoptera*	Tovel	27/02/2012	water column	46.261402873	10.951116085	50µm	KC619024
65PD1TovelA	*Polyarthra*	*dolichoptera*	Tovel	02/08/2010	water column	46.261402873	10.951116085	50µm	KC619023
65PD2TovelA	*Polyarthra*	*dolichoptera*	Tovel	02/08/2010	water column	46.261402873	10.951116085	50µm	KC619022
65PD3TovelA	*Polyarthra*	*dolichoptera*	Tovel	02/08/2010	water column	46.261402873	10.951116085	50µm	KC619020
65PD4TovelA	*Polyarthra*	*dolichoptera*	Tovel	02/08/2010	water column	46.261402873	10.951116085	50µm	KC619021
65PD5TovelA	*Polyarthra*	*dolichoptera*	Tovel	02/08/2010	water column	46.261402873	10.951116085	50µm	KC618974
65PD6TovelA	*Polyarthra*	*dolichoptera*	Tovel	02/08/2010	water column	46.261402873	10.951116085	50µm	KC619019
65PD7TovelA	*Polyarthra*	*dolichoptera*	Tovel	02/08/2010	water column	46.261402873	10.951116085	50µm	KC619018
65PD8TovelA	*Polyarthra*	*dolichoptera*	Tovel	02/08/2010	water column	46.261402873	10.951116085	50µm	KC619017
66PD2TovelA	*Polyarthra*	*dolichoptera*	Tovel	08/10/2011	water column	46.261402873	10.951116085	50µm	KC619016
66PD4TovelA	*Polyarthra*	*dolichoptera*	Tovel	08/10/2011	water column	46.261402873	10.951116085	50µm	KC619015
66PD5TovelA	*Polyarthra*	*dolichoptera*	Tovel	08/10/2011	water column	46.261402873	10.951116085	50µm	KC618976
66PD6TovelA	*Polyarthra*	*dolichoptera*	Tovel	08/10/2011	water column	46.261402873	10.951116085	50µm	KC618906
66PD7TovelA	*Polyarthra*	*dolichoptera*	Tovel	08/10/2011	water column	46.261402873	10.951116085	50µm	KC619014
66PD8TovelA	*Polyarthra*	*dolichoptera*	Tovel	08/10/2011	water column	46.261402873	10.951116085	50µm	KC619013
67PD3TovelB	*Polyarthra*	*dolichoptera*	Tovel	08/10/2011	water column	46.257211776	10.944786072	50µm	KC619012
67PD4TovelB	*Polyarthra*	*dolichoptera*	Tovel	08/10/2011	water column	46.257211776	10.944786072	50µm	KC619010
67PD5TovelB	*Polyarthra*	*dolichoptera*	Tovel	08/10/2011	water column	46.257211776	10.944786072	50µm	KC619011
67PD6TovelB	*Polyarthra*	*dolichoptera*	Tovel	08/10/2011	water column	46.257211776	10.944786072	50µm	KC618907
82PD1Corredo	*Polyarthra*	*dolichoptera*	Corredo	04/04/2012	water column	46.35486628	11.10434532	50µm	KC618879
82PD2Corredo	*Polyarthra*	*dolichoptera*	Corredo	04/04/2012	water column	46.35486628	11.10434532	50µm	KC618880
82PD3Corredo	*Polyarthra*	*dolichoptera*	Corredo	04/04/2012	water column	46.236377641	10.581808090	50µm	KC618882
82PD4Corredo	*Polyarthra*	*dolichoptera*	Corredo	04/04/2012	water column	46.35486628	11.10434532	50µm	KC618881
82PD5Corredo	*Polyarthra*	*dolichoptera*	Corredo	04/04/2012	water column	46.35486628	11.10434532	50µm	KC618883
82PD6Corredo	*Polyarthra*	*dolichoptera*	Corredo	04/04/2012	water column	46.236377641	10.581808090	50µm	KC618885
82PD8Corredo	*Polyarthra*	*dolichoptera*	Corredo	04/04/2012	water column	46.35486628	11.10434532	50µm	KC618884
85PD3Crescino2	*Polyarthra*	*dolichoptera*	Crescino	04/04/2012	water column	46.244391706	11.059412956	50µm	KC618886
85PD4Crescino2	*Polyarthra*	*dolichoptera*	Crescino	04/04/2012	water column	46.244391706	11.059412956	50µm	KC618887
85PD6Crescino2	*Polyarthra*	*dolichoptera*	Crescino	04/04/2012	water column	46.244391706	11.059412956	50µm	KC618889
85PD7Crescino2	*Polyarthra*	*dolichoptera*	Crescino	04/04/2012	water column	46.244391706	11.059412956	50µm	KC618888
88PD1Tovel	*Polyarthra*	*dolichoptera*	Tovel	04/04/2012	water column	46.261402873	10.951116085	50µm	KC618951

88PD2Tovel	*Polyarthra*	*dolichoptera*	Tovel	04/04/2012	water column	46.261402873	10.951116085	50µm	KC618955
88PD3Tovel	*Polyarthra*	*dolichoptera*	Tovel	04/04/2012	water column	46.261402873	10.951116085	50µm	KC618954
88PD4Tovel	*Polyarthra*	*dolichoptera*	Tovel	04/04/2012	water column	46.261402873	10.951116085	50µm	KC618957
88PD5Tovel	*Polyarthra*	*dolichoptera*	Tovel	04/04/2012	water column	46.261402873	10.951116085	50µm	KC618952
88PD6Tovel	*Polyarthra*	*dolichoptera*	Tovel	04/04/2012	water column	46.261402873	10.951116085	50µm	KC618953
88PD7Tovel	*Polyarthra*	*dolichoptera*	Tovel	04/04/2012	water column	46.261402873	10.951116085	50µm	KC618959
88PD8Tovel	*Polyarthra*	*dolichoptera*	Tovel	04/04/2012	water column	46.261402873	10.951116085	50µm	KC618958
90PD1TovelA5	*Polyarthra*	*dolichoptera*	Tovel	17/04/2012	5m	46.261402873	10.951116085	50µm	KC618890
90PD3TovelA5	*Polyarthra*	*dolichoptera*	Tovel	17/04/2012	5m	46.261402873	10.951116085	50µm	KC618891
90PD6TovelA5	*Polyarthra*	*dolichoptera*	Tovel	17/04/2012	5m	46.261402873	10.951116085	50µm	KC618965
90PD7TovelA5	*Polyarthra*	*dolichoptera*	Tovel	17/04/2012	5m	46.261402873	10.951116085	50µm	KC618892
90PD8TovelA5	*Polyarthra*	*dolichoptera*	Tovel	17/04/2012	5m	46.261402873	10.951116085	50µm	KC618893
91PD1TovelA10	*Polyarthra*	*dolichoptera*	Tovel	17/04/2012	10m	46.261402873	10.951116085	50µm	KC618967
91PD2TovelA10	*Polyarthra*	*dolichoptera*	Tovel	17/04/2012	10m	46.261402873	10.951116085	50µm	KC618968
91PD3TovelA10	*Polyarthra*	*dolichoptera*	Tovel	17/04/2012	10m	46.261402873	10.951116085	50µm	KC618962
91PD4TovelA10	*Polyarthra*	*dolichoptera*	Tovel	17/04/2012	10m	46.261402873	10.951116085	50µm	KC618966
91PD5TovelA10	*Polyarthra*	*dolichoptera*	Tovel	17/04/2012	10m	46.261402873	10.951116085	50µm	KC618960
91PD6TovelA10	*Polyarthra*	*dolichoptera*	Tovel	17/04/2012	10m	46.261402873	10.951116085	50µm	KC618961
91PD7TovelA10	*Polyarthra*	*dolichoptera*	Tovel	17/04/2012	10m	46.261402873	10.951116085	50µm	KC618998
91PD8TovelA10	*Polyarthra*	*dolichoptera*	Tovel	17/04/2012	10m	46.261402873	10.951116085	50µm	KC618969
95PD1TovelA20	*Polyarthra*	*dolichoptera*	Tovel	17/04/2012	20m	46.261402873	10.951116085	50µm	KC618996
95PD3TovelA20	*Polyarthra*	*dolichoptera*	Tovel	17/04/2012	20m	46.261402873	10.951116085	50µm	KC618988
95PD4TovelA20	*Polyarthra*	*dolichoptera*	Tovel	17/04/2012	20m	46.261402873	10.951116085	50µm	KC618948
95PD5TovelA20	*Polyarthra*	*dolichoptera*	Tovel	17/04/2012	20m	46.261402873	10.951116085	50µm	KC618956
95PD6TovelA20	*Polyarthra*	*dolichoptera*	Tovel	17/04/2012	20m	46.261402873	10.951116085	50µm	KC618970
95PD7TovelA20	*Polyarthra*	*dolichoptera*	Tovel	17/04/2012	20m	46.261402873	10.951116085	50µm	KC618971
95PD8TovelA20	*Polyarthra*	*dolichoptera*	Tovel	17/04/2012	20m	46.261402873	10.951116085	50µm	KC618997
96PD1TovelA4	*Polyarthra*	*dolichoptera*	Tovel	17/04/2012	5m	46.261402873	10.951116085	50µm	KC618894
96PD4TovelA4	*Polyarthra*	*dolichoptera*	Tovel	17/04/2012	5m	46.261402873	10.951116085	50µm	KC619000
96PD5TovelA4	*Polyarthra*	*dolichoptera*	Tovel	17/04/2012	5m	46.261402873	10.951116085	50µm	KC618999
96PD7TovelA4	*Polyarthra*	*dolichoptera*	Tovel	17/04/2012	5m	46.261402873	10.951116085	50µm	KC618963
96PD8TovelA4	*Polyarthra*	*dolichoptera*	Tovel	17/04/2012	5m	46.261402873	10.951116085	50µm	KC618964

1 accession missing
