# Supplementary material for: SEXUAL SPECIES ARE SEPARATED BY LARGER GENETIC GAPS THAN ASEXUAL SPECIES IN ROTIFERS
Source: Evolution. 2014 Jul 25;68(10):2901–16. doi: 10.1111/evo.12483 (PMC4262011; doi:10.1111/evo.12483)
Supplement: Table S1 — . Specimen collection information and accessions numbers for the sequences generated for this study. Table S2. Summary information for each of the 13 datasets, including number of sequences, number of unique haplotypes, estimated diversity, constancy of diversification statistics, estimated ages, and accessions. Table S3. Intra- and interspecific diversity measures (genetic and phylogenetic distances) for each of the delimited GMYC entities. Table S4. Datasets were split up by sister clades as determined using the backbone phylogeny (Fig.2) and outgroup taxa were added to balance the sequence numbers for each alignment. Table S5. Output from GLMM analysis of GMYC model fit (P value) differences between bdelloid and monogonont rotifers and varying degrees of jackknifing. Figure S1. Phylogenetic methods workflow. Figure S2. Minimum interspecific genetic distance (raw pDistance) against minimum phylogenetic distance to the nearest neighbor (Myr). Figure S3. Species richness of each of the 13 rotifer datasets analyzed by GMYC but with different input ultrametric trees. Figure S4. Significance of the GMYC model fit when bdelloid (red) and monogonont (blue) trees are jackknifed by 20%, 25%, 33%, and 50%. File S1. Are the ultrametric trees robust to rate heterogeneity? File S2. How does phylogenetic reconstruction method affect species delimitation? File S3. Is sampling effort differentially affecting bdelloid and monogonont diversity estimates? [file evo0068-2901-SD1.zip › evo12483-sup-0007-tableS2.pdf]

Table S2. Summary information for each of the 13 data sets, including number of sequences, number of unique haplotypes, estimated diversity, constancy of diversification statistics, estimated ages, and accessions.

Taxon	Group	Taxonomic scale	bp	N seq.	N hapl.	GMYC analyses		# GMYC entities				N singl.	N seq./ ESU	N hapl./ ESU	Estimated richness (chao)	Diversification rate					Age (MYR)	Backbone GenBank		TreeBASE
						LR	*P* value	Bayesian								γ	MCCR crit. val.	*P* value	CorSiM γ	*P* value		18S rDNA	COI mtDNA	
								1°	relaxed	strict	ML													
Adineta spp.	Bdelloidea	Genus	661	229	115	29.21	2.02E-06	70	71	64	70	44	3.3	1.6	119.8	-6.21	-2.7	0.0001	-3.95	0.00004	27.58	KF561095	KF582480	66181
Ascomorpha spp.	Monogononta	Genus	661	38	13	8.28	0.04	5	6	5	5	2	7.6	2.6	6	-1.01	-1.15	0.06	-0.7	0.24	56.13	DQ297691	KC618773	66185
Brachionus calyciflorus	Monogononta	Species complex	712	460	173	96.85	0	17	19	21	8	4	27.1	10.2	20	1.56	-1.37	0.92	2.45	0.99	25.61	KF561096	HQ444170	66189
Brachionus plicatilis	Monogononta	Species complex	729	460	129	32.61	3.89E-07	20	33	23	26	6	23.0	6.5	27.5	-3.15	-1.71	0.003	-3.68	0.00012	25.84	KF561097	HQ444171	66208
Dissotrocha spp.	Bdelloidea	Genus	661	38	27	10.21	0.017	19	18	11	19	12	2.0	1.4	31	-2.6	-1.97	0.011	-2.89	0.0019	20.99	KF561098-KF561099	KF582497, KJ913822	66212
Keratella cochlearis	Monogononta	Species complex	661	59	23	12.38	0.0062	6	6	3	6	2	9.8	3.8	7	-1.72	-1.01	0.011	-2.21	0.014	24.30	DQ297697	KC618852	66216
Macrotrachela spp.	Bdelloidea	Genus	661	194	80	17.19	0.00065	46	50	44	44	29	4.2	1.7	92.7	-5.28	-2.68	0.0001	-2.96	0.0015	25.39	N/A	N/A	66220
Philodina spp.	Bdelloidea	Genus	661	386	139	57.96	1.61E-12	71	74	72	72	43	5.4	2.0	128.8	-4.35	-2.84	0.0002	-2.71	0.0034	53.95	KF561100-KF561102	JQ309165, JQ309181, JQ309183	66224
Pleuretra lineata	Bdelloidea	Species complex	661	48	20	7.29	0.063	8	5	5	18	4	6.0	2.5	14	1.96	-1.76	0.98	1.96	0.97	22.98	JX494746	FJ426426	66228
Polyarthra spp.	Monogononta	Genus	661	462	53	37.49	3.63E-08	18	18	18	18	10	25.7	2.9	40.5	-1.93	-2.22	0.084	-1.69	0.046	45.85	DQ297716	DQ297789	66233
Rotaria spp.	Bdelloidea	Genus	698	1541	147	14.17	0.0027	120	119	116	117	97	12.8	1.2	344	-5.32	-4.68	0.009	-2.74	0.003	32.62	KF561103-KF561105	EU076828, JQ309460, JQ309578	66237
Synchaeta spp.	Monogononta	Genus	678	210	32	11.40	0.0097	14	14	14	14	6	15.0	2.3	18.5	-1.22	-1.46	0.082	-0.97	0.17	28.57	KF561106-KF561107	JN936673, JN936516	66241
Testudinella spp.	Monogononta	Genus	661	86	34	38.01	2.82E-08	13	11	11	12	8	6.6	2.6	27	-0.4	-2.14	0.57	-0.25	0.4	42.89	KF561108-KF561109	HQ444166, HQ444168	66245

Abbreviations: bp - length of alignment; N seq. - Number of sequences; N hap. - Number of haplotypes; LR - Likelihood ratio; ML - Maximum likelihood; BI - Bayesian inference; N singl. - Number of singletons; MCCR crit. val. - Monte Carlo constant rates critical value.
