# Supplementary material for: SEXUAL SPECIES ARE SEPARATED BY LARGER GENETIC GAPS THAN ASEXUAL SPECIES IN ROTIFERS
Source: Evolution. 2014 Jul 25;68(10):2901–16. doi: 10.1111/evo.12483 (PMC4262011; doi:10.1111/evo.12483)
Supplement: Table S1 — . Specimen collection information and accessions numbers for the sequences generated for this study. Table S2. Summary information for each of the 13 datasets, including number of sequences, number of unique haplotypes, estimated diversity, constancy of diversification statistics, estimated ages, and accessions. Table S3. Intra- and interspecific diversity measures (genetic and phylogenetic distances) for each of the delimited GMYC entities. Table S4. Datasets were split up by sister clades as determined using the backbone phylogeny (Fig.2) and outgroup taxa were added to balance the sequence numbers for each alignment. Table S5. Output from GLMM analysis of GMYC model fit (P value) differences between bdelloid and monogonont rotifers and varying degrees of jackknifing. Figure S1. Phylogenetic methods workflow. Figure S2. Minimum interspecific genetic distance (raw pDistance) against minimum phylogenetic distance to the nearest neighbor (Myr). Figure S3. Species richness of each of the 13 rotifer datasets analyzed by GMYC but with different input ultrametric trees. Figure S4. Significance of the GMYC model fit when bdelloid (red) and monogonont (blue) trees are jackknifed by 20%, 25%, 33%, and 50%. File S1. Are the ultrametric trees robust to rate heterogeneity? File S2. How does phylogenetic reconstruction method affect species delimitation? File S3. Is sampling effort differentially affecting bdelloid and monogonont diversity estimates? [file evo0068-2901-SD1.zip › evo12483-sup-0008-tableS3.pdf]

Table S3. Intra- and interspecific diversity measures (genetic and phylogenetic distances) for each of the delimited GMYC entities. The number of haplotypes per GMYC entity, morphospecies, habitat type, and population genetic measures are also shown. A minimum of four sequences was requried for D^*, F^*, F_S, and D, and a minumum of two sequences was required for R_2.

Group	Taxon	GMYC ent.	N hapl.	Morphospecies	Hab.	Intraspecific		Min. interspecific		Population genetics				
						pDist.	TMRCA (Myr)	pDist.	Phylogenetic dist. (Myr)	D^*	F^*	F_S	D	R_2
Bdelloidea	*Adineta* spp.	1	3	sp.	L	0.0140	1.542	0.082	13.453	-	-	-	-	0.20
		2	2	*vaga*	L	0.0211	0.797	0.078	23.462	-	-	-	-	0.50
		3	2	sp.	L	0.0053	1.393	0.037	3.608	-	-	-	-	0.50
		4	2	*vaga*	L	0.0053	0.584	0.037	3.608	-	-	-	-	0.50
		5	2	*vaga*	L	0.0053	1.350	0.091	9.471	-	-	-	-	0.50
		6	3	*vaga*	L	0.0070	0.729	0.082	8.108	-	-	-	-	0.31
		7	2	*vaga*	L	0.0053	0.157	0.066	9.728	-	-	-	-	0.50
		8	2	*vaga*	L	0.0053	0.591	0.021	3.864	-	-	-	-	0.50
		9	3	*vaga*	L	0.0105	0.296	0.066	14.173	-	-	-	-	0.09
		10	2	*vaga*	L	0.0263	1.377	0.053	3.758	-	-	-	-	0.50
		11	2	*vaga*	L	0.0053	1.359	0.07	19.883	-	-	-	-	0.50
		12	2	*grandis*	L	0.0316	0.701	0.082	10.918	-	-	-	-	0.50
		13	5	*vaga*	L	0.0158	2.366	0.008	3.433	0.31	0.33	0.00	0.31	0.16
		14	3	*vaga*	L	0.1368	2.176	0.012	3.511	-	-	-	-	0.37
		15	2	*vaga*	L	0.0105	0.890	0.074	16.802	-	-	-	-	0.50
		16	2	*vaga*	L	0.0053	0.415	0.091	5.744	-	-	-	-	0.50
		17	6	*vaga*	L	0.0179	2.155	0.058	4.666	-0.77	-0.83	-1.66	-0.76	0.12
		18	5	sp.	L	0.0137	1.291	0.082	12.353	-1.03	-1.10	-0.92	-1.03	0.23
		19	2	*vaga*	L	0.0105	0.662	0.095	9.629	-	-	-	-	0.50
		20	3	*vaga*	L	0.0211	1.738	0.07	13.025	-	-	-	-	0.21
		21	3	*gracilis*	L	0.0105	0.865	0.115	6.457	-	-	-	-	0.23
		22	2	*gracilis*	L	0.0053	0.305	0.07	4.886	-	-	-	-	0.50
		23	5	*steineri*	L	0.0232	2.474	0.012	3.518	-0.67	-0.72	-0.12	-0.67	0.11
		24	2	sp.	L	0.0053	1.792	0.086	6.531	-	-	-	-	0.50
		25	2	*vaga*	L	0.0053	0.638	0.074	10.924	-	-	-	-	0.50
		26	2	sp.	L	0.0053	0.116	0.045	4.817	-	-	-	-	0.50
		27	1	*gracilis*	L	-	-	0.066	6.531	-	-	-	-	-
		28	1	sp.	L	-	-	0.082	13.612	-	-	-	-	-
		29	1	sp.	L	-	-	0.111	13.718	-	-	-	-	-
		30	1	sp.	L	-	-	0.123	13.718	-	-	-	-	-
		31	1	sp.	L	-	-	0.082	9.471	-	-	-	-	-
		32	1	sp.	L	-	-	0.049	12.605	-	-	-	-	-
		33	1	sp.	L	-	-	0.045	4.115	-	-	-	-	-
		34	1	sp.	L	-	-	0.045	4.115	-	-	-	-	-
		35	1	*vaga*	L	-	-	0.062	11.681	-	-	-	-	-
		36	1	*vaga*	L	-	-	0.066	6.700	-	-	-	-	-
		37	1	*vaga*	L	-	-	0.091	8.637	-	-	-	-	-
		38	1	*vaga*	L	-	-	0.053	3.758	-	-	-	-	-
		39	1	*vaga*	L	-	-	0.058	4.666	-	-	-	-	-
		40	1	*vaga*	L	-	-	0.091	8.108	-	-	-	-	-
		41	1	*vaga*	L	-	-	0.033	3.022	-	-	-	-	-
		42	1	*vaga*	L	-	-	0.033	3.022	-	-	-	-	-
		43	1	*vaga*	L	-	-	0.021	3.864	-	-	-	-	-
		44	1	*vaga*	L	-	-	0.086	6.573	-	-	-	-	-
		45	1	*barbata*	L	-	-	0.074	12.605	-	-	-	-	-
		46	1	*vaga*	L	-	-	0.008	3.433	-	-	-	-	-
		47	1	*vaga*	L	-	-	0.078	8.849	-	-	-	-	-
		48	1	*vaga*	L	-	-	0.012	3.511	-	-	-	-	-
		49	1	sp.	L	-	-	0.045	4.817	-	-	-	-	-
		50	1	sp.	L	-	-	0.062	11.681	-	-	-	-	-
		51	1	sp.	L	-	-	0.078	7.655	-	-	-	-	-
		52	1	*ricciae*	L	-	-	0.099	17.537	-	-	-	-	-
		53	1	sp.	L	-	-	0.123	5.744	-	-	-	-	-
		54	1	*steineri*	L	-	-	0.012	4.363	-	-	-	-	-
		55	1	*steineri*	L	-	-	0.025	3.518	-	-	-	-	-
		56	1	*vaga*	L	-	-	0.078	9.929	-	-	-	-	-
		57	1	*vaga*	L	-	-	0.078	11.943	-	-	-	-	-
		58	1	*vaga*	L	-	-	0.062	6.573	-	-	-	-	-
		59	1	*vaga*	L	-	-	0.091	9.108	-	-	-	-	-
		60	1	*vaga*	L	-	-	0.062	9.929	-	-	-	-	-
		61	1	*vaga*	L	-	-	0.086	17.602	-	-	-	-	-
		62	1	*gracilis*	L	-	-	0.07	4.886	-	-	-	-	-
		63	1	*steineri*	L	-	-	0.082	13.241	-	-	-	-	-
		64	1	*steineri*	L	-	-	0.078	8.479	-	-	-	-	-
		65	1	*tuberculosa*	L	-	-	0.115	10.924	-	-	-	-	-
		66	1	*vaga*	L	-	-	0.082	7.655	-	-	-	-	-
		67	1	*vaga*	L	-	-	0.082	13.241	-	-	-	-	-
		68	1	*vaga*	L	-	-	0.095	8.637	-	-	-	-	-
		69	1	*vaga*	L	-	-	0.086	9.629	-	-	-	-	-
		70	1	*oculata*	L	-	-	0.095	17.537	-	-	-	-	-
	Dissotrocha spp.	1	2	*macrostyla*	A	0.0058	0.378	0.12	9.783	-	-	-	-	0.50

	2	2	*macrostyla*	A	0.0058	0.287	0.137	12.795	-	-	-	-	0.50
	3	2	*aculeata*	A	0.0058	0.801	0.076	3.905	-	-	-	-	0.50
	4	2	*aculeata*	A	0.0117	0.346	0.155	9.501	-	-	-	-	0.50
	5	3	*aculeata*	A	0.0078	0.676	0.078	5.023	-	-	-	-	0.28
	6	2	*aculeata*	A	0.0175	0.998	0.128	5.305	-	-	-	-	0.50
	7	2	*macrostyla*	A	0.0058	0.407	0.173	3.678	-	-	-	-	0.50
	8	1	*aculeata*	A	-	-	0.128	5.305	-	-	-	-	-
	9	1	*aculeata*	A	-	-	0.078	5.023	-	-	-	-	-
	10	1	*macrostyla*	A	-	-	0.12	9.783	-	-	-	-	-
	11	1	*macrostyla*	A	-	-	0.154	7.534	-	-	-	-	-
	12	1	*aculeata*	A	-	-	0.184	9.419	-	-	-	-	-
	13	1	*aculeata*	A	-	-	0.129	13.688	-	-	-	-	-
	14	1	*aculeata*	A	-	-	0.076	3.905	-	-	-	-	-
	15	1	*macrostyla*	A	-	-	0.147	7.534	-	-	-	-	-
	16	1	*macrostyla*	A	-	-	0.147	3.678	-	-	-	-	-
	17	1	*macrostyla*	A	-	-	0.02	2.511	-	-	-	-	-
	18	1	*macrostyla*	A	-	-	0.02	2.511	-	-	-	-	-
	19	1	*aculeata*	A	-	-	0.124	9.501	-	-	-	-	-
Macrotrachela spp.	1	3	*quadricornifera*	L	0.0100	1.157	0.105	10.602	-	-	-	-	0.03
	2	2	*latior*	L	0.0199	1.086	0.094	10.062	-	-	-	-	0.50
	3	3	*multispinosa*	L	0.0133	1.115	0.082	11.184	-	-	-	-	0.25
	4	2	*quadricornifera*	L	0.0050	2.820	0.061	4.808	-	-	-	-	0.50
	5	5	*quadricornifera*	L	0.0159	0.676	0.034	3.705	0.41	0.43	-0.71	0.41	0.17
	6	2	*quadricornifera*	L	0.0100	0.363	0.072	7.753	-	-	-	-	0.50
	7	2	*quadricornifera*	L	0.0348	1.752	0.054	7.053	-	-	-	-	0.50
	8	2	*quadricornifera*	L	0.0199	2.746	0.011	4.213	-	-	-	-	0.50
	9	2	*quadricornifera*	L	0.0050	0.648	0.064	9.595	-	-	-	-	0.50
	10	2	*quadricornifera*	L	0.0100	1.933	0.064	7.494	-	-	-	-	0.50
	11	7	*quadricornifera*	L	0.0171	2.579	0.076	15.177	-0.17	-0.14	-2.03	0.04	0.16
	12	3	*quadricornifera*	L	0.0133	2.130	0.082	13.146	-	-	-	-	0.35
	13	2	*quadricornifera*	L	0.0050	0.942	0.095	7.535	-	-	-	-	0.50
	14	3	sp.	L	0.0232	2.346	0.085	11.762	-	-	-	-	0.20
	15	2	*latior*	L	0.0050	0.818	0.112	8.723	-	-	-	-	0.50
	16	5	*latior*	L	0.0199	1.401	0.112	8.723	0.03	0.00	-0.29	-0.11	0.16
	17	4	sp.	L	0.0100	2.755	0.057	11.542	-0.84	-0.90	-0.22	-1.08	0.19
	18	1	sp.	L	-	-	0.117	11.680	-	-	-	-	-
	19	1	sp.	L	-	-	0.082	4.162	-	-	-	-	-
	20	1	sp.	L	-	-	0.089	4.162	-	-	-	-	-
	21	1	sp.	L	-	-	0.098	7.939	-	-	-	-	-
	22	1	sp.	L	-	-	0.105	8.469	-	-	-	-	-
	23	1	sp.	L	-	-	0.108	8.469	-	-	-	-	-
	24	1	*musculosa*	L	-	-	0.104	10.602	-	-	-	-	-
	25	1	sp.	L	-	-	0.13	22.979	-	-	-	-	-
	26	1	*latior*	L	-	-	0.094	10.062	-	-	-	-	-
	27	1	sp.	L	-	-	0.098	12.832	-	-	-	-	-
	28	1	*plicata*	A	-	-	0.086	12.100	-	-	-	-	-
	29	1	*plicata*	A	-	-	0.04	3.475	-	-	-	-	-
	30	1	*plicata*	A	-	-	0.04	3.475	-	-	-	-	-
	31	1	*ehrenbergi*	L	-	-	0.128	11.116	-	-	-	-	-
	32	1	*multispinosa*	L	-	-	0.121	15.980	-	-	-	-	-
	33	1	*papillosa*	L	-	-	0.082	11.184	-	-	-	-	-
	34	1	*multispinosa*	L	-	-	0.087	13.086	-	-	-	-	-
	35	1	*quadricornifera*	L	-	-	0.088	13.823	-	-	-	-	-
	36	1	*quadricornifera*	L	-	-	0.061	4.808	-	-	-	-	-
	37	1	*quadricornifera*	L	-	-	0.034	3.705	-	-	-	-	-
	38	1	*quadricornifera*	L	-	-	0.054	7.053	-	-	-	-	-
	39	1	*quadricornifera*	L	-	-	0.011	4.213	-	-	-	-	-
	40	1	*quadricornifera*	L	-	-	0.069	7.494	-	-	-	-	-
	41	1	*quadricornifera*	L	-	-	0.098	7.535	-	-	-	-	-
	42	1	*bullata*	L	-	-	0.085	9.225	-	-	-	-	-
	43	1	sp.	L	-	-	0.102	8.368	-	-	-	-	-
	44	1	sp.	L	-	-	0.091	8.368	-	-	-	-	-
	45	1	*habita*	L	-	-	0.057	11.542	-	-	-	-	-
	46	1	*ehrenbergi*	L	-	-	0.098	25.392	-	-	-	-	-
Philodina spp.	1	2	sp.	A	0.0032	0.155	0.060	4.382	-	-	-	-	0.50
	2	2	sp.	A	0.0032	0.208	0.060	4.382	-	-	-	-	0.50
	3	3	sp.	-	0.0130	2.255	0.076	13.427	-	-	-	-	0.42
	4	7	sp.	A	0.0155	2.113	0.076	10.082	-0.66	-0.75	-1.14	-0.74	0.12
	5	2	sp.	A	0.0097	0.668	0.063	6.608	-	-	-	-	0.50
	6	2	sp.	A	0.0195	1.204	0.063	6.608	-	-	-	-	0.50
	7	2	sp.	-	0.0032	0.472	0.066	6.438	-	-	-	-	0.50
	8	2	sp.	-	0.0097	1.316	0.028	3.219	-	-	-	-	0.50
	9	3	sp.	L	0.0195	2.124	0.028	3.219	-	-	-	-	0.19
	10	5	sp.	-	0.0234	1.759	0.085	9.373	0.09	0.09	-0.31	0.09	0.15
	11	2	sp.	L	0.0065	0.478	0.107	14.003	-	-	-	-	0.50
	12	13	*plena*	-	0.0208	2.226	0.120	22.742	-0.28	-0.43	-5.40	-0.63	0.12
	13	5	*plena*	L	0.0091	2.048	0.088	27.575	-1.07	-1.18	-0.71	-1.23	0.16

	14	2	*rugosa*	A	0.0065	0.256	0.108	12.157	-	-	-	-	0.50
	15	2	sp.	A	0.0065	1.900	0.073	13.511	-	-	-	-	0.50
	16	3	*duplicalcar*	A	0.0216	1.977	0.098	28.268	-	-	-	-	0.25
	17	2	*citrina*	A	0.0032	0.714	0.101	8.131	-	-	-	-	0.50
	18	2	*citrina*	A	0.0260	1.185	0.093	7.308	-	-	-	-	0.50
	19	2	*citrina*	-	0.0065	0.290	0.046	6.630	-	-	-	-	0.50
	20	2	*flaviceps*	A	0.0032	0.872	0.101	9.075	-	-	-	-	0.50
	21	3	*flaviceps*	A	0.0087	1.028	0.126	10.291	-	-	-	-	0.27
	22	5	*flaviceps*	A	0.0084	0.751	0.129	10.291	0.06	0.07	-0.45	0.06	0.18
	23	2	*flaviceps*	A	0.0032	0.152	0.130	9.562	-	-	-	-	0.50
	24	3	*flaviceps*	A	0.0152	0.964	0.019	3.663	-	-	-	-	0.34
	25	2	*flaviceps*	A	0.0097	0.262	0.120	9.857	-	-	-	-	0.50
	26	2	sp.	A	0.0065	0.147	0.088	13.756	-	-	-	-	0.50
	27	9	sp.	A	0.0159	1.429	0.082	9.283	-0.60	-0.61	-2.27	-0.37	0.15
	28	5	sp.	A	0.0175	2.292	0.085	14.871	-0.42	-0.45	0.06	-0.42	0.12
	29	1	*flaviceps*	A	-	-	0.019	3.663	-	-	-	-	-
	30	1	*flaviceps*	A	-	-	0.045	7.463	-	-	-	-	-
	31	1	*flaviceps*	A	-	-	0.101	9.562	-	-	-	-	-
	32	1	*flaviceps*	A	-	-	0.098	20.668	-	-	-	-	-
	33	1	*citrina*	A	-	-	0.104	10.542	-	-	-	-	-
	34	1	*citrina*	A	-	-	0.103	8.131	-	-	-	-	-
	35	1	*citrina*	A	-	-	0.114	18.013	-	-	-	-	-
	36	1	sp.	A	-	-	0.103	9.075	-	-	-	-	-
	37	1	*plena*	A	-	-	0.091	22.742	-	-	-	-	-
	38	1	*plena*	A	-	-	0.111	13.479	-	-	-	-	-
	39	1	*plena*	A	-	-	0.069	3.018	-	-	-	-	-
	40	1	*plena*	A	-	-	0.095	24.145	-	-	-	-	-
	41	1	*plena*	A	-	-	0.107	18.159	-	-	-	-	-
	42	1	*acuticornis*	A	-	-	0.089	9.283	-	-	-	-	-
	43	1	*vorax*	A	-	-	0.069	3.018	-	-	-	-	-
	44	1	*citrina*	A	-	-	0.046	6.630	-	-	-	-	-
	45	1	*roseola*	A	-	-	0.061	13.427	-	-	-	-	-
	46	1	sp.	-	-	-	0.076	9.494	-	-	-	-	-
	47	1	sp.	-	-	-	0.088	9.494	-	-	-	-	-
	48	1	sp.	A	-	-	0.066	6.438	-	-	-	-	-
	49	1	sp.	A	-	-	0.082	9.373	-	-	-	-	-
	50	1	sp.	-	-	-	0.079	14.003	-	-	-	-	-
	51	1	sp.	A	-	-	0.108	18.509	-	-	-	-	-
	52	1	sp.	A	-	-	0.082	16.509	-	-	-	-	-
	53	1	sp.	A	-	-	0.104	12.157	-	-	-	-	-
	54	1	sp.	A	-	-	0.082	13.756	-	-	-	-	-
	55	1	sp.	A	-	-	0.153	4.566	-	-	-	-	-
	56	1	sp.	A	-	-	0.130	4.566	-	-	-	-	-
	57	1	sp.	A	-	-	0.085	42.194	-	-	-	-	-
	58	1	sp.	-	-	-	0.161	10.597	-	-	-	-	-
	59	1	sp.	-	-	-	0.076	9.857	-	-	-	-	-
	60	1	*rugosa*	A	-	-	0.088	24.859	-	-	-	-	-
	61	1	*citrina*	A	-	-	0.110	8.802	-	-	-	-	-
	62	1	*megalotrocha*	L	-	-	0.110	5.160	-	-	-	-	-
	63	1	*megalotrocha*	L	-	-	0.063	11.546	-	-	-	-	-
	64	1	*megalotrocha*	A	-	-	0.134	26.114	-	-	-	-	-
	65	1	*megalotrocha*	A	-	-	0.134	26.114	-	-	-	-	-
	66	1	*megalotrocha*	A	-	-	0.115	5.160	-	-	-	-	-
	67	1	*citrina*	A	-	-	0.093	7.308	-	-	-	-	-
	68	1	*megalotrocha*	A	-	-	0.063	11.546	-	-	-	-	-
	69	1	sp.	A	-	-	0.076	10.082	-	-	-	-	-
	70	1	sp.	A	-	-	0.070	8.812	-	-	-	-	-
	71	1	sp.	A	-	-	0.073	13.511	-	-	-	-	-
Pleuretra lineata	1	4	*lineata*	L	0.0000	0.161	0.005	0.849	-0.61	-0.48	0.17	-0.61	0.43
	2	3	*lineata*	L	0.0000	0.170	0.005	0.849	-	-	-	-	0.47
	3	4	*lineata*	L	0.0013	0.207	0.003	0.406	-0.71	-0.60	1.10	-0.71	0.43
	4	5	*lineata*	L	0.0010	0.167	0.039	2.349	-0.97	-0.95	-0.83	-0.97	0.24
	5	1	*lineata*	L	-	-	0.008	0.855	-	-	-	-	-
	6	1	*lineata*	L	-	-	0.008	0.855	-	-	-	-	-
	7	1	*lineata*	L	-	-	0.003	0.406	-	-	-	-	-
	8	1	*lineata*	L	-	-	0.13	22.979	-	-	-	-	-
Rotaria spp.	1	2	*sordida*	L	0.0123	0.793	0.091	23.270	-	-	-	-	0.50
	2	2	*rotatoria*	A	0.0061	0.236	0.09	6.159	-	-	-	-	0.50
	3	2	*rotatoria*	A	0.0061	0.409	0.077	11.123	-	-	-	-	0.50
	4	2	*rotatoria*	A	0.0123	0.868	0.091	6.901	-	-	-	-	5.00
	5	2	*rotatoria*	A	0.0061	0.258	0.12	17.586	-	-	-	-	0.50
	6	2	*rotatoria*	A	0.0184	0.406	0.034	4.481	-	-	-	-	0.50
	7	2	*rotatoria*	A	0.0061	0.700	0.065	13.448	-	-	-	-	0.50
	8	2	*rotatoria*	A	0.0123	0.338	0.046	5.672	-	-	-	-	0.50
	9	2	*rotatoria*	A	0.0061	0.127	0.072	3.354	-	-	-	-	0.50
	10	2	*rotatoria*	A	0.0123	0.711	0.085	13.645	-	-	-	-	0.50
	11	2	*magnacalcarata*	A	0.0184	0.671	0.089	7.778	-	-	-	-	0.50

12	2	*rotatoria*	A	0.0307	0.383	0.04	3.937	-	-	-	-	0.50
13	4	*rotatoria*	A	0.0164	0.797	0.064	7.555	-0.53	-0.53	-0.48	-0.53	0.05
14	2	*rotatoria*	A	0.0123	0.317	0.059	8.298	-	-	-	-	0.50
15	2	*macrura*	A	0.0184	0.573	0.065	15.737	-	-	-	-	0.50
16	4	*rotatoria*	A	0.0266	1.061	0.076	8.821	1.96	2.04	0.64	1.96	0.30
17	2	*rotatoria*	A	0.0061	0.754	0.071	4.073	-	-	-	-	0.50
18	2	*rotatoria*	A	0.0061	0.594	0.076	4.073	-	-	-	-	0.50
19	2	*rotatoria*	A	0.0123	0.288	0.131	13.002	-	-	-	-	0.50
20	2	*neptunia*	A	0.0184	0.970	0.089	7.694	-	-	-	-	0.50
21	2	*tardigrada*	A	0.0368	0.684	0.046	1.930	-	-	-	-	0.50
22	2	*tardigrada*	A	0.0061	0.102	0.059	6.036	-	-	-	-	0.50
23	2	*sordida*	L	0.0061	0.824	0.052	5.817	-	-	-	-	0.50
24	1	*socialis*	L	-	-	0.085	23.270	-	-	-	-	-
25	1	*rotatoria*	A	-	-	0.072	8.004	-	-	-	-	-
26	1	*rotatoria*	A	-	-	0.085	8.004	-	-	-	-	-
27	1	*rotatoria*	A	-	-	0.077	9.894	-	-	-	-	-
28	1	*rotatoria*	A	-	-	0.077	6.159	-	-	-	-	-
29	1	*rotatoria*	A	-	-	0.065	6.901	-	-	-	-	-
30	1	*rotatoria*	A	-	-	0.084	17.586	-	-	-	-	-
31	1	*rotatoria*	A	-	-	0.099	21.579	-	-	-	-	-
32	1	*rotatoria*	A	-	-	0.07	10.361	-	-	-	-	-
33	1	*rotatoria*	A	-	-	0.034	4.481	-	-	-	-	-
34	1	*magnacalcarata*	A	-	-	0.076	16.163	-	-	-	-	-
35	1	*rotatoria*	A	-	-	0.046	5.672	-	-	-	-	-
36	1	*rotatoria*	A	-	-	0.064	7.604	-	-	-	-	-
37	1	*rotatoria*	A	-	-	0.071	5.682	-	-	-	-	-
38	1	*rotatoria*	A	-	-	0.083	4.507	-	-	-	-	-
39	1	*rotatoria*	A	-	-	0.083	4.507	-	-	-	-	-
40	1	*rotatoria*	A	-	-	0.071	3.354	-	-	-	-	-
41	1	*rotatoria*	A	-	-	0.085	16.155	-	-	-	-	-
42	1	*citrina*	A	-	-	0.092	16.155	-	-	-	-	-
43	1	*rotatoria*	A	-	-	0.089	8.732	-	-	-	-	-
44	1	*rotatoria*	A	-	-	0.105	8.732	-	-	-	-	-
45	1	*rotatoria*	A	-	-	0.058	3.127	-	-	-	-	-
46	1	*rotatoria*	A	-	-	0.063	3.127	-	-	-	-	-
47	1	*rotatoria*	A	-	-	0.058	7.494	-	-	-	-	-
48	1	*rotatoria*	A	-	-	0.066	9.635	-	-	-	-	-
49	1	*rotatoria*	A	-	-	0.111	6.822	-	-	-	-	-
50	1	*rotatoria*	A	-	-	0.077	6.822	-	-	-	-	-
51	1	*rotatoria*	A	-	-	0.064	9.589	-	-	-	-	-
52	1	*rotatoria*	A	-	-	0.071	16.508	-	-	-	-	-
53	1	*rotatoria*	A	-	-	0.058	7.213	-	-	-	-	-
54	1	*rotatoria*	A	-	-	0.058	7.213	-	-	-	-	-
55	1	*rotatoria*	A	-	-	0.083	12.481	-	-	-	-	-
56	1	*rotatoria*	A	-	-	0.087	12.481	-	-	-	-	-
57	1	*rotatoria*	A	-	-	0.072	13.645	-	-	-	-	-
58	1	*magnacalcarata*	A	-	-	0.017	1.601	-	-	-	-	-
59	1	*magnacalcarata*	A	-	-	0.017	1.601	-	-	-	-	-
60	1	*rotatoria*	A	-	-	0.017	3.365	-	-	-	-	-
61	1	*rotatoria*	A	-	-	0.017	3.365	-	-	-	-	-
62	1	*rotatoria*	A	-	-	0.059	2.749	-	-	-	-	-
63	1	*rotatoria*	A	-	-	0.04	2.749	-	-	-	-	-
64	1	*rotatoria*	A	-	-	0.04	3.937	-	-	-	-	-
65	1	*rotatoria*	A	-	-	0.053	8.363	-	-	-	-	-
66	1	*socialis*	A	-	-	0.078	8.210	-	-	-	-	-
67	1	sp.	-	-	-	0.066	8.210	-	-	-	-	-
68	1	*rotatoria*	A	-	-	0.064	7.555	-	-	-	-	-
69	1	*rotatoria*	A	-	-	0.034	5.246	-	-	-	-	-
70	1	*rotatoria*	A	-	-	0.023	3.883	-	-	-	-	-
71	1	*rotatoria*	A	-	-	0.023	2.576	-	-	-	-	-
72	1	*rotatoria*	A	-	-	0.023	2.576	-	-	-	-	-
73	1	*rotatoria*	A	-	-	0.059	10.566	-	-	-	-	-
74	1	*rotatoria*	A	-	-	0.065	10.566	-	-	-	-	-
75	1	*rotatoria*	A	-	-	0.083	8.298	-	-	-	-	-
76	1	*rotatoria*	A	-	-	0.097	8.821	-	-	-	-	-
77	1	*rotatoria*	A	-	-	0.082	15.474	-	-	-	-	-
78	1	*rotatoria*	A	-	-	0.017	2.281	-	-	-	-	-
79	1	*rotatoria*	A	-	-	0.017	1.459	-	-	-	-	-
80	1	*rotatoria*	A	-	-	0.017	1.459	-	-	-	-	-
81	1	*rotatoria*	A	-	-	0.07	20.587	-	-	-	-	-
82	1	*rotatoria*	A	-	-	0.073	23.233	-	-	-	-	-
83	1	*macrura*	A	-	-	0.099	23.233	-	-	-	-	-
84	1	*rotatoria*	A	-	-	0.077	23.578	-	-	-	-	-
85	1	*macrura*	A	-	-	0.083	17.161	-	-	-	-	-
86	1	*macrura*	A	-	-	0.104	5.020	-	-	-	-	-
87	1	*macrura*	A	-	-	0.083	5.020	-	-	-	-	-
88	1	*macrura*	A	-	-	0.09	10.500	-	-	-	-	-

		89	1	*macrura*	A	-	-	0.09	18.775	-	-	-	-	-
		90	1	sp.	-	-	-	0.106	25.435	-	-	-	-	-
		91	1	*neptunia*	A	-	-	0.089	5.301	-	-	-	-	-
		92	1	*neptunia*	A	-	-	0.097	5.301	-	-	-	-	-
		93	1	*tardigrada*	A	-	-	0.04	3.665	-	-	-	-	-
		94	1	*tardigrada*	A	-	-	0.04	1.930	-	-	-	-	-
		95	1	sp.	-	-	-	0.058	5.123	-	-	-	-	-
		96	1	*tardigrada*	A	-	-	0.071	4.114	-	-	-	-	-
		97	1	*tardigrada*	A	-	-	0.076	4.114	-	-	-	-	-
		98	1	sp.	-	-	-	0.072	7.533	-	-	-	-	-
		99	1	*tardigrada*	A	-	-	0.09	7.533	-	-	-	-	-
		100	1	*tardigrada*	A	-	-	0.059	8.782	-	-	-	-	-
		101	1	*tardigrada*	A	-	-	0.053	2.238	-	-	-	-	-
		102	1	*tardigrada*	L	-	-	0.053	2.238	-	-	-	-	-
		103	1	*tardigrada*	A	-	-	0.065	6.036	-	-	-	-	-
		104	1	*tardigrada*	A	-	-	0.065	10.732	-	-	-	-	-
		105	1	*tardigrada*	A	-	-	0.023	1.368	-	-	-	-	-
		106	1	*tardigrada*	A	-	-	0.023	1.368	-	-	-	-	-
		107	1	*tardigrada*	A	-	-	0.04	4.949	-	-	-	-	-
		108	1	*tardigrada*	A	-	-	0.089	8.469	-	-	-	-	-
		109	1	*tardigrada*	A	-	-	0.071	14.896	-	-	-	-	-
		110	1	*sordida*	L	-	-	0.059	10.342	-	-	-	-	-
		111	1	*sordida*	L	-	-	0.052	10.028	-	-	-	-	-
		112	1	*sordida*	L	-	-	0.058	2.738	-	-	-	-	-
		113	1	*sordida*	L	-	-	0.059	2.738	-	-	-	-	-
		114	1	*sordida*	L	-	-	0.052	4.948	-	-	-	-	-
		115	1	*sordida*	L	-	-	0.077	4.948	-	-	-	-	-
		116	1	*sordida*	L	-	-	0.057	4.369	-	-	-	-	-
		117	1	*sordida*	L	-	-	0.052	4.369	-	-	-	-	-
		118	1	*sordida*	L	-	-	0.052	6.478	-	-	-	-	-
		119	1	*sordida*	L	-	-	0.057	6.478	-	-	-	-	-
		120	1	*sordida*	L	-	-	0.052	12.723	-	-	-	-	-
Monogononta	*Ascomorpha* spp.	1	5	*ecaudis*	A	0.0221	2.948	0.346	37.895	-0.51	-0.58	0.18	-0.61	0.24
		2	3	*ecaudis*	A	0.0070	1.338	0.162	19.133	-	-	-	-	0.29
		3	3	*ovalis*	A	0.0129	1.345	0.162	30.750	-	-	-	-	0.41
		4	1	*ovalis*	A	-	-	0.19	19.133	-	-	-	-	-
		5	1	*ovalis*	A	-	-	0.253	56.130	-	-	-	-	-
	Brachionus calyciflorus	1	6	*calyciflorus*	A	0.0053	0.724	0.009	1.173	0.72	0.78	-2.94	0.76	0.21
		2	26	*calyciflorus*	A	0.0081	0.675	0.009	1.094	-4.01	-4.17	-31.76	-2.56	0.05
		3	29	*calyciflorus*	A	0.0092	0.919	0.013	1.094	-4.17	-4.18	-32.27	-2.27	0.03
		4	2	*calyciflorus*	A	0.0018	0.159	0.011	3.282	-	-	-	-	0.50
		5	9	*calyciflorus*	A	0.0089	0.737	0.072	8.043	-1.31	-1.41	-5.00	-1.11	0.09
		6	8	*calyciflorus*	A	0.0046	0.502	0.002	1.492	-1.92	-2.09	-5.95	-1.76	0.04
		7	15	*calyciflorus*	A	0.0067	0.745	0.028	2.911	-2.50	-2.63	-12.49	-1.72	0.06
		8	6	*calyciflorus*	A	0.0055	0.509	0.011	1.753	-1.25	-1.36	-2.36	-1.25	0.10
		9	7	*calyciflorus*	A	0.0067	0.550	0.017	1.857	-1.18	-1.28	-3.65	-1.10	0.12
		10	33	*calyciflorus*	A	0.0078	0.842	0.006	1.059	-4.55	-4.57	-41.91	-2.51	0.02
		11	21	*calyciflorus*	A	0.0085	0.912	0.006	1.059	-2.87	-3.16	-19.50	-2.32	0.04
		12	3	*calyciflorus*	A	0.0037	0.605	0.201	11.947	-	-	-	-	0.00
		13	4	*calyciflorus*	A	0.0028	0.531	0.21	25.607	-0.80	-0.75	-1.51	-0.80	0.17
		14	1	*calyciflorus*	A	-	-	0.002	1.492	-	-	-	-	-
		15	1	*calyciflorus*	A	-	-	0.013	1.368	-	-	-	-	-
		16	1	*calyciflorus*	A	-	-	0.011	3.282	-	-	-	-	-
		17	1	*calyciflorus*	A	-	-	0.011	1.753	-	-	-	-	-
	Brachionus plicatilis	1	4	*rotundiformis*	A	0.0047	0.218	0.155	6.181	-0.80	-0.75	-1.51	-0.80	0.17
		2	4	*rotundiformis*	A	0.0907	4.013	0.193	11.613	-0.20	-0.25	2.19	-0.38	0.15
		3	3	*plicatilis*	A	0.0268	1.595	0.238	17.231	-	-	-	-	0.36
		4	8	*Austria*	A	0.0286	2.870	0.146	12.838	0.10	0.09	-1.11	0.00	0.16
		5	3	*Nevada*	A	0.0710	2.142	0.198	5.496	-	-	-	-	0.38
		6	2	*plicatilis*	A	0.0187	0.758	0.097	5.496	-	-	-	-	0.50
		7	8	*manjavacas*	A	0.0307	3.678	0.131	21.064	-1.04	-1.18	-1.14	-1.12	0.19
		8	63	*plicatilis*	A	0.0376	2.624	0.209	23.380	-1.23	-1.28	-34.29	-0.82	0.08
		9	5	*Towerinniensis*	A	0.0213	1.526	0.097	8.513	-0.47	-0.50	-0.04	-0.47	0.14
		10	6	*ibericus*	A	0.0278	1.573	0.131	11.088	0.73	0.85	-0.24	0.94	0.21
		11	7	*Almenara*	A	0.0212	2.107	0.171	11.088	0.49	0.50	-1.30	0.31	0.17
		12	4	*Tiscar*	A	0.0047	0.463	0.126	9.810	-0.81	-0.78	-1.24	-0.81	0.25
		13	3	*Cayman*	A	0.0062	0.315	0.146	7.092	-	-	-	-	0.36
		14	3	*plicatilis*	A	0.0100	0.285	0.204	7.092	-	-	-	-	0.18
		15	1	*rotundiformis*	A	-	-	0.13	6.181	-	-	-	-	-
		16	1	*plicatilis*	A	-	-	0.116	8.513	-	-	-	-	-
		17	1	*Harvey*	A	-	-	0.155	18.554	-	-	-	-	-
		18	1	*Tiscar*	A	-	-	0.116	6.229	-	-	-	-	-
		19	1	*Tiscar*	A	-	-	0.126	6.229	-	-	-	-	-
		20	1	*quadridentatus*	A	-	-	0.133	25.841	-	-	-	-	-
	Keratella cochlearis	1	6	*cochlearis*	A	0.0222	2.013	0.13	9.084	-0.76	-0.85	-0.62	-0.84	0.13
		2	4	*cochlearis*	A	0.0073	0.402	0.144	15.808	1.00	1.00	-0.29	1.00	0.21
		3	6	*cochlearis*	A	0.0180	2.035	0.144	15.808	0.25	0.28	-0.55	0.25	0.20

	4	5	*cochlearis*	A	0.0274	1.655	0.219	14.804	1.04	1.12	0.29	1.04	0.22
	5	1	*cochlearis*	A	-	-	0.13	9.084	-	-	-	-	-
	6	1	*cochlearis*	A	-	-	0.219	14.804	-	-	-	-	-
Polyarthra spp.	1	5	*dolichoptera*	A	0.0061	0.375	0.121	12.861	0.76	0.79	-2.12	0.76	0.22
	2	2	*dolichoptera*	A	0.0133	0.652	0.094	4.305	-	-	-	-	0.50
	3	4	*dolichoptera*	A	0.0174	1.803	0.179	23.721	0.71	0.73	0.39	0.71	0.21
	4	3	*dolichoptera*	A	0.0114	1.326	0.142	19.706	-	-	-	-	0.25
	5	4	*dolichoptera*	A	0.0124	0.617	0.065	4.409	-0.85	-0.86	-0.10	-0.85	0.13
	6	6	*dolichoptera*	A	0.0108	0.942	0.065	4.409	-1.05	-1.13	-1.46	-1.02	0.19
	7	13	*dolichoptera*	A	0.0259	2.100	0.127	8.445	-0.80	-0.74	-3.95	-0.23	0.12
	8	6	*dolichoptera*	A	0.0091	1.835	0.127	8.445	-0.27	-0.32	-1.44	-0.38	0.12
	9	1	*dolichoptera*	A	-	-	0.172	20.591	-	-	-	-	-
	10	1	sp.	A	-	-	0.152	22.219	-	-	-	-	-
	11	1	*dolichoptera*	A	-	-	0.152	10.017	-	-	-	-	-
	12	1	*dolichoptera*	A	-	-	0.142	19.706	-	-	-	-	-
	13	1	*dolichoptera*	A	-	-	0.172	23.721	-	-	-	-	-
	14	1	*dolichoptera*	A	-	-	0.129	8.697	-	-	-	-	-
	15	1	*dolichoptera*	A	-	-	0.094	4.305	-	-	-	-	-
	16	1	*dolichoptera*	A	-	-	0.129	8.697	-	-	-	-	-
	17	1	*remata*	A	-	-	0.152	22.219	-	-	-	-	-
	18	1	*remata*	A	-	-	0.19	29.907	-	-	-	-	-
Synchaeta spp.	1	2	*lakowitziana*	A	0.0036	0.218	0.243	25.517	-	-	-	-	0.50
	2	2	*kitina*	A	0.0145	1.730	0.112	14.557	-	-	-	-	0.50
	3	7	*pectinata*	A	0.0199	2.201	0.074	4.536	-0.19	-0.25	-1.21	-0.34	0.16
	4	2	*pectinata*	A	0.0091	0.282	0.059	3.635	-	-	-	-	0.50
	5	4	*tremula-oblonga*	A	0.0076	0.993	0.078	7.054	-0.56	-0.56	-0.36	-0.56	0.11
	6	3	*tremula-oblonga*	A	0.0109	1.051	0.078	7.054	-	-	-	-	0.33
	7	2	*tremula-oblonga*	A	0.0036	0.673	0.065	5.394	-	-	-	-	0.50
	8	4	*cecilia*	A	0.0060	0.621	0.192	20.548	-0.15	-0.15	-0.57	-0.15	0.12
	9	1	*pectinata*	A	-	-	0.069	4.536	-	-	-	-	-
	10	1	*pectinata*	A	-	-	0.059	3.635	-	-	-	-	-
	11	1	*pectinata*	A	-	-	0.11	8.991	-	-	-	-	-
	12	1	*tremula-oblonga*	A	-	-	0.133	12.712	-	-	-	-	-
	13	1	*tremula-oblonga*	A	-	-	0.065	5.394	-	-	-	-	-
	14	1	*grandis*	A	-	-	0.251	28.565	-	-	-	-	-
Testudinella spp.	1	2	*clypeata*	A	0.0000	0.063	0.18	14.524	-	-	-	-	0.50
	2	4	*clypeata*	A	0.0106	0.365	0.188	8.442	-0.56	-0.56	-0.36	-0.56	0.21
	3	4	*clypeata*	A	0.0138	0.533	0.038	2.427	-0.21	-0.20	-1.41	-0.21	0.25
	4	3	*clypeata*	A	0.0085	0.445	0.017	0.853	-	-	-	-	0.25
	5	13	*clypeata*	A	0.0038	0.472	0.017	0.853	-1.40	-1.59	-2.05	-1.38	0.12
	6	1	*patina*	A	-	-	0.244	11.821	-	-	-	-	-
	7	1	*patina*	A	-	-	0.245	11.821	-	-	-	-	-
	8	1	*patina*	A	-	-	0.244	19.579	-	-	-	-	-
	9	1	*patina*	A	-	-	0.256	20.157	-	-	-	-	-
	10	1	*caeca*	A	-	-	0.277	37.600	-	-	-	-	-
	11	1	*clypeata*	A	-	-	0.18	18.283	-	-	-	-	-
	12	1	*clypeata*	A	-	-	0.213	7.832	-	-	-	-	-
	13	1	*clypeata*	A	-	-	0.281	26.498	-	-	-	-	-
