# Supplementary material for: SEXUAL SPECIES ARE SEPARATED BY LARGER GENETIC GAPS THAN ASEXUAL SPECIES IN ROTIFERS
Source: Evolution. 2014 Jul 25;68(10):2901–16. doi: 10.1111/evo.12483 (PMC4262011; doi:10.1111/evo.12483)
Supplement: Table S1 — . Specimen collection information and accessions numbers for the sequences generated for this study. Table S2. Summary information for each of the 13 datasets, including number of sequences, number of unique haplotypes, estimated diversity, constancy of diversification statistics, estimated ages, and accessions. Table S3. Intra- and interspecific diversity measures (genetic and phylogenetic distances) for each of the delimited GMYC entities. Table S4. Datasets were split up by sister clades as determined using the backbone phylogeny (Fig.2) and outgroup taxa were added to balance the sequence numbers for each alignment. Table S5. Output from GLMM analysis of GMYC model fit (P value) differences between bdelloid and monogonont rotifers and varying degrees of jackknifing. Figure S1. Phylogenetic methods workflow. Figure S2. Minimum interspecific genetic distance (raw pDistance) against minimum phylogenetic distance to the nearest neighbor (Myr). Figure S3. Species richness of each of the 13 rotifer datasets analyzed by GMYC but with different input ultrametric trees. Figure S4. Significance of the GMYC model fit when bdelloid (red) and monogonont (blue) trees are jackknifed by 20%, 25%, 33%, and 50%. File S1. Are the ultrametric trees robust to rate heterogeneity? File S2. How does phylogenetic reconstruction method affect species delimitation? File S3. Is sampling effort differentially affecting bdelloid and monogonont diversity estimates? [file evo0068-2901-SD1.zip › evo12483-sup-0009-tableS4.pdf]

Table S4. Data sets were split up by sister clades as determined using the backbone phylogeny (Fig. 2) and outgroup taxa were added to balance the sequence numbers for each alignment.

Sister clade	Group	Alignment 1	# Seq.	Group	Alignment 2	# Seq.
1	Monogononta	*Ascomorpha*	13	Monogononta	*Keratella cochlearis*	23
2	Monogononta	*Polyarthra*	53	Monogononta	*Synchaeta*	32
3	Monogononta	*Brachionus calyciflorus*	173	Monogononta	*Brachionus plicatilis*	129
4	Bdelloidea	*Philodina*	139	Bdelloidea	*Pleuretra lineata*	20
5	Bdelloidea	*Dissotrocha*	27	Bdelloidea	*Rotaria*	147
	Monogononta	*Testudinella*	34	Bdelloidea	*Adineta*	115
	Bdelloidea	*Macrotrachela*	80			
		Total	519			466
